# Stochastic quasi-gradient methods: variance reduction via Jacobian sketching

**DOI:** 10.1007/s10107-020-01506-0

**Published:** 2020-05-12

**Authors:** Robert M. Gower, Peter Richtárik, Francis Bach

**Affiliations:** 1 LTCI, Telécom Paris, Institut Polytechnique de Paris, Palaiseau, France; 2 King Abdullah University of Science and Technology (KAUST), Thuwal, Saudi Arabia; 3 University of Edinburgh, Edinburgh, UK; 4 Moscow Institute of Physics and Technology (MIPT), Dolgoprudny, Russia; 5 INRIA - ENS - PSL Research University, Paris, France

**Keywords:** Stochastic gradient descent, Sketching, Variance reduction, Covariates, 65Kxx, 90C15, 90C25

## Abstract

We develop a new family of variance reduced stochastic gradient descent methods for minimizing the average of a very large number of smooth functions. Our method—JacSketch—is motivated by novel developments in randomized numerical linear algebra, and operates by maintaining a stochastic estimate of a Jacobian matrix composed of the gradients of individual functions. In each iteration, JacSketch efficiently updates the Jacobian matrix by first obtaining a random linear measurement of the true Jacobian through (cheap) sketching, and then projecting the previous estimate onto the solution space of a linear matrix equation whose solutions are consistent with the measurement. The Jacobian estimate is then used to compute a variance-reduced unbiased estimator of the gradient. Our strategy is analogous to the way quasi-Newton methods maintain an estimate of the Hessian, and hence our method can be seen as a *stochastic quasi-gradient method*. Our method can also be seen as stochastic gradient descent applied to a *controlled stochastic optimization reformulation* of the original problem, where the control comes from the Jacobian estimates. We prove that for smooth and strongly convex functions, JacSketch converges linearly with a meaningful rate dictated by a single convergence theorem which applies to general sketches. We also provide a refined convergence theorem which applies to a smaller class of sketches, featuring a novel proof technique based on a *stochastic Lyapunov function*. This enables us to obtain sharper complexity results for variants of JacSketch with importance sampling. By specializing our general approach to specific sketching strategies, JacSketch reduces to the celebrated stochastic average gradient (SAGA) method, and its several existing and many new minibatch, reduced memory, and importance sampling variants. Our rate for SAGA with importance sampling is the current best-known rate for this method, resolving a conjecture by Schmidt et al. (Proceedings of the eighteenth international conference on artificial intelligence and statistics, AISTATS 2015, San Diego, California, 2015). The rates we obtain for minibatch SAGA are also superior to existing rates and are sufficiently tight as to show a decrease in total complexity as the minibatch size increases. Moreover, we obtain the first minibatch SAGA method with importance sampling.

## Introduction

We consider the problem of minimizing the average of a large number of differentiable functions

1 $$\begin{aligned} x^*=\arg \min _{x \in {\mathbb {R}}^d} \left[ f(x) \overset{\text {def}}{=}\frac{1}{n}\sum _{i=1}^n f_i(x) \right] , \end{aligned}$$

where *f* is $\mu$—strongly convex and *L*—smooth. In solving (1), we restrict our attention to first-order methods that use a (variance-reduced) stochastic estimate of the gradient $g^k \approx \nabla f(x^k)$ to take a step towards minimizing (1) by iterating

2 $$\begin{aligned} x^{k+1} = x^k - \alpha g^{k}, \end{aligned}$$

where $\alpha >0$ is a stepsize.

In the context of machine learning, (1) is an abstraction of the *empirical risk minimization* problem; *x* encodes the parameters/features of a (statistical) model, and $f_i$ is the loss of example/data point *i* incurred by model *x*. The goal is to find the model *x* which minimizes the average loss on the *n* observations.

Typically, *n* is so large that algorithms which rely on scanning through all *n* functions in each iteration are too costly. The need for incremental methods for the training phase of machine learning models has revived the interest in the stochastic gradient descent (SGD) method [27]. SGD sets $g^k=\nabla f_i(x^k)$, where *i* is an index chosen from $[n]\overset{\text {def}}{=}\{1,2,\ldots ,n\}$ uniformly at random. SGD therefore requires only a single data sample to complete a step and make progress towards the solution. Thus SGD scales well in the number of data samples, which is important in several machine learning applications since there many be a large number of data samples. On the downside, the variance of the stochastic estimates of the gradient produced by SGD does not vanish during the iterative process, which suggests that a decreasing stepsize regime needs to be put into place if SGD is to converge. Furthermore, for SGD to work efficiently, this decreasing stepsize regime needs to be tuned for each application area, which is costly.

### Variance-reduced methods

Stochastic variance-reduced versions of SGD offer a solution to this high variance issue, which improves the theoretical convergence rate and solves the issue with ad hoc stepsize regimes. The first variance reduced method for empirical risk minimization is the stochastic average gradient (SAG) method of Schmidt, Le Roux and Bach [29], closely followed by Finito [7] and Miso [18]. The analysis of SAG is notoriously difficult, which is perhaps due to the estimator of gradient being biased. Soon afterwards, the SAG gradient estimator was modified into an unbiased one, which resulted in the SAGA method [6]. The analysis of SAGA is dramatically simpler than that of SAG. Another popular method is SVRG of Johnson and Zhang [15] (see also S2GD [16]). SVRG enjoys the same theoretical complexity bound as SAGA, but has a much smaller memory footprint. It is based on an inner–outer loop procedure. In the outer loop, a full pass over data is performed to compute the gradient of *f* at the current point. In the inner loop, this gradient is modified with the use of cheap stochastic gradients, and steps are taken in the direction of the modified gradients. A notable recent addition to the family of variance reduced methods, developed by Nguyen et al. [20], is known as SARAH. Unlike other methods, SARAH does not use an estimator that is unbiased in the last step. Instead, it is unbiased over a long history of the method.

A fundamentally different way of designing variance reduced methods is to use coordinate descent [24, 25] to solve the dual. This is what the SDCA method [33] and its various extensions [32] do. The key advantage of this approach is that the dual often has a seperable structure in the coordinate space, which in turn means that each iteration of coordinate descent is cheap. Furthermore, SDCA is a variance-reduced method by design since the coordinates of the gradient tend to zero as one approaches the solution. One of the downsides of SDCA is that it requires calculating Fenchel duals and their derivatives. This issue was later solved by introducing approximations and mapping the dual iterates to the primal space as pointed out in [6]. This resulted in primal variants of SDCA such as dual-free SDCA [31]. A primal-dual variant which enables the use of arbitrary minibatch strategies was developed by Qu et al. [23], and is known as QUARTZ.

Finally, variance reduced methods can also be accelerated, as has been shown for the loop based methods such as Katyusha [3] or using the Universal catalyst [17].

### Gaps in our understanding of SAGA

Despite significant research into variance-reduced stochastic gradient descent methods for solving (1), there are still big gaps in our understanding of variance reduction. For instance, the current theory supporting the SAGA algorithm is far from complete.

SAGA with uniform probabilities enjoys the iteration complexity ${{\mathcal {O}}}( (n+ \tfrac{L_{\max }}{\mu } ) \log \tfrac{1}{\epsilon })$, where $L_{\max } \overset{\text {def}}{=}\max _i L_i$ and $L_i$ is the smoothness constant of $f_i$. While importance sampling versions of SAGA have proved in practice to produce a speed-up over uniform SAGA [30], a proof of this speed-up has been elusive. It was conjectured by Schmidt et al. [30] that a properly designed importance sampling strategy for SAGA should lead to the rate ${{\mathcal {O}}}\left( \left( n+ \tfrac{\bar{L}}{\mu } \right) \log \tfrac{1}{\epsilon }\right)$, where $\bar{L}=\tfrac{1}{n}\sum _i L_i$. However, no such result was proved. This rate is achieved by, for instance, importance sampling variants of SDCA, QUARTZ [23] and SVRG [36]. However, the analysis only applies to a more specialized version of problem (1) (e.g., one needs an explicit strongly convex regularizer).

Second, existing minibatch variants of SAGA do not enjoy the same rate as that offered by methods such as SDCA and QUARTZ. Are the above issues with SAGA unavoidable, or is it the case that our understanding of the method is far from complete? Lastly, no minibatch variant of SAGA with importance sampling is known.

One of the contributions of this paper is giving positive answers to all of the above questions.

### Jacobian sketching: a new approach to variance reduction

Our key contribution in this paper is the introduction of a novel approach—which we call *Jacobian sketching*—to designing and understanding variance-reduced stochastic gradient descent methods for solving (1). We refer to our method by the name *JacSketch*. We shall now briefly introduce some of the key insights motivating our approach. Let $F:{\mathbb {R}}^d\rightarrow {\mathbb {R}}^n$ be defined by

3 $$\begin{aligned} F(x) \overset{\text {def}}{=}(f_1(x), \ldots , f_n(x)) \in {\mathbb {R}}^n, \end{aligned}$$

and further let

4 $$\begin{aligned} {\varvec{\nabla }}{} \mathbf{F}(x) \overset{\text {def}}{=}[\nabla f_1(x), \ldots , \nabla f_n(x)] \in {\mathbb {R}}^{d\times n}, \end{aligned}$$

be the Jacobian of *F* at *x*.

The starting point of our new approach is the following trivial observation: the gradient of *f* at *x* can be computed from the Jacobian ${\varvec{\nabla }}{} \mathbf{F}(x)$ by a simple *linear transformation:*

5 $$\begin{aligned} \frac{1}{n}{\varvec{\nabla }}{} \mathbf{F}(x) e= \nabla f(x), \end{aligned}$$

where $e$ is the vector of all ones in ${\mathbb {R}}^n$. This alone is not useful to come up with a better way of estimating the gradient. Indeed, formula (5) has two issues. First, the Jacobian is *not* available. If we wanted to compute it, we would need to pay the cost of one pass through the data. Second, even if the Jacobian was available, merely multiplying it by the vector of all ones would cost ${{\mathcal {O}}}(nd)$ operations, which is again a cost equivalent to one pass over data.

Now, let us replace the vector of all ones in (5) by $e_i \in {\mathbb {R}}^n$, the unit coordinate/basis vector in ${\mathbb {R}}^n$. If the index *i* is chosen randomly from [*n*], then

6 $$\begin{aligned} {\varvec{\nabla }}{} \mathbf{F}(x)e_i = \nabla f_i(x), \end{aligned}$$

which is a stochastic gradient of *f* at *x*. In other words, by performing a *random linear transformation* of the Jacobian, we have arrived at the classical stochastic estimate of the gradient. This approach does not suffer from the first issue mentioned above as the Jacobian is *not needed* at all in order to compute $\nabla f_i(x)$. Likewise, it does not suffer from the second issue; namely, the cost of computing the stochastic gradient is merely ${{\mathcal {O}}}(d)$, and we can avoid a costly pass through the data.^1^

However, this approach suffers from a new issue: by constructing the estimate this way, we *do not learn* from the (random) information collected about the Jacobian in prior iterations, through having access to random linear transformations thereof. In this paper we take the point of view that this is the reason why SGD suffers from large variance. Our approach towards alleviating this problem is to maintain and update an estimate $\mathbf{J}\in {\mathbb {R}}^{d \times n}$ of the Jacobian ${\varvec{\nabla }}{} \mathbf{F}(x).$

Given $x^k \in {\mathbb {R}}^d$, ideally we would like $\mathbf{J}$ to satisfy

7 $$\begin{aligned} \mathbf{J}= {\varvec{\nabla }}{} \mathbf{F}(x^k), \end{aligned}$$

that is, we would like it to be equal to the true Jacobian. However, at the same time we do not wish to pay the price of computing it. Hence, assuming we have an estimate $\mathbf{J}^k\in {\mathbb {R}}^{d\times n}$ of the Jacobian available, we instead pick a random matrix ${\mathbf {S}}_k\in {\mathbb {R}}^{n\times \tau }$ from some distribution ${{\mathcal {D}}}$ of matrices^2^ and consider the following *sketched* version of the linear system (7), with unknown $\mathbf{J}$:

8 $$\begin{aligned} \mathbf{J}{\mathbf {S}}_k = {\varvec{\nabla }}{} \mathbf{F}(x^k) {\mathbf {S}}_k \in {\mathbb {R}}^{d\times \tau }. \end{aligned}$$

This equation generalizes both (5) and (6). The left hand side contains the sketched system matrix ${\mathbf {S}}_k$ and the unknown matrix $\mathbf{J}$, and the right hand side contains a quantity we can measure (through a random linear measurement of the Jacobian, which we assume is cheap). Of course, the true Jacobian solves (8). However, in general, and in particular when $\tau \ll n$ which is the regime we want to be in for practical reasons, the system (8) will have infinite $\mathbf{J}$ solutions.

We pick a unique solution $\mathbf{J}^{k+1}$ as the closest solution of (8) to our previous estimate $\mathbf{J}^k$, with respect to a weighted Frobenius norm with a positive definite weight matrix $\mathbf{W}\in {\mathbb {R}}^{n\times n}$:

9 $$\begin{aligned} \mathbf{J}^{k+1}= & {} \arg \min _{\mathbf{J}\in {\mathbb {R}}^{d\times n}} \Vert \mathbf{J}- \mathbf{J}^k\Vert _{\mathbf{W}^{-1}} \nonumber \\&\text {subject to} \quad \mathbf{J}{\mathbf {S}}_k = {\varvec{\nabla }}{} \mathbf{F}(x^k) {\mathbf {S}}_k, \end{aligned}$$

where

10 $$\begin{aligned} \left\| \mathbf{X} \right\| _{\mathbf{W}^{-1}} \overset{\text {def}}{=}\sqrt{\text{ Tr }\left( \mathbf{X}\mathbf{W}^{-1} \mathbf{X}^\top \right) }. \end{aligned}$$

In doing so, we have built a learning mechanism whose goal is to maintain good estimates of the Jacobian throughout the run of method (2). These estimates can be used to efficiently estimate the gradient by performing a linear transformation similar to (5), but with ${\varvec{\nabla }}{} \mathbf{F}(x)$ replaced by the latest estimate of the Jacobian. In practice, it is important to design sketching matrices so that the Jacobian sketch ${\varvec{\nabla }}{} \mathbf{F}(x){\mathbf {S}}_k$ can be calculated efficiently.

The “sketch-and-project” strategy (9) for updating our Jacobian estimate is analogous to the way quasi-Newton methods update the estimate of the Hessian (or inverse Hessian) [8, 9, 12]. From this perspective, our method can be viewed as a *stochastic quasi-gradient method*.^3^

Problem (9) admits the explicit closed-form solution (see Lemma 14):

11 $$\begin{aligned} \mathbf{J}^{k+1} = \mathbf{J}^{k} +({\varvec{\nabla }}{} \mathbf{F}(x^k)-\mathbf{J}^{k}) {\varvec{\Pi }}_{{\mathbf {S}}_k}, \end{aligned}$$

where

12 $$\begin{aligned} {\varvec{\Pi }}_{\mathbf {S}}\overset{\text {def}}{=}{\mathbf {S}}({\mathbf {S}}^\top \mathbf{W}{\mathbf {S}})^{\dagger } {\mathbf {S}}^\top \mathbf{W}, \end{aligned}$$

is a projection matrix, and $\dagger$ denotes the Moore–Penrose pseudoinverse.

The key insight of our work is to propose an efficient Jacobian learning mechanism based on ideas borrowed from recent results in randomized numerical linear algebra.

Having established our update of the Jacobian estimate, we now need to use this to form an estimate of the gradient. Unfortunately, using $\mathbf{J}^{k+1}$ in place of ${\varvec{\nabla }}{} \mathbf{F}(x^k)$ in (5) leads to a biased gradient estimate (something we explore later in Sect. 2.5). To obtain an unbiased estimator of the gradient, we introduce a *stochastic relaxation parameter* $\theta _{{\mathbf {S}}_k}\ge 0$ and use

13 $$\begin{aligned} g^k \overset{\text {def}}{=}\frac{1-\theta _{{\mathbf {S}}_k}}{n} \mathbf{J}^k e+ \frac{\theta _{{\mathbf {S}}_k}}{n} \mathbf{J}^{k+1} e= \frac{1}{n}\mathbf{J}^k e+ \frac{1}{n}\left( {\varvec{\nabla }}{} \mathbf{F}(x^k) - \mathbf{J}^k\right) \theta _{{\mathbf {S}}_k} {\varvec{\Pi }}_{{\mathbf {S}}_k} e, \end{aligned}$$

as an approximation of the gradient. Taking expectations in (13) over ${\mathbf {S}}^k\sim {{\mathcal {D}}}$ (for this we use the notation ${\mathbb {E}}_{{{\mathcal {D}}}}\left[ \cdot \right] \equiv {\mathbb {E}}_{{\mathbf {S}}_k\sim {{\mathcal {D}}}}\left[ \cdot \right]$), we get

14 $$\begin{aligned} {\mathbb {E}}_{{{\mathcal {D}}}}\left[ g^k\right] = \frac{1}{n}\mathbf{J}^k e+ \frac{1}{n}({\varvec{\nabla }}{} \mathbf{F}(x^k) - \mathbf{J}^k) {\mathbb {E}}_{{{\mathcal {D}}}}\left[ \theta _{{\mathbf {S}}_k} {\varvec{\Pi }}_{{\mathbf {S}}_k} e\right] . \end{aligned}$$

Thus provided that

15 $$\begin{aligned} {\mathbb {E}}_{{{\mathcal {D}}}}\left[ \theta _{{\mathbf {S}}_k} {\varvec{\Pi }}_{{\mathbf {S}}_k} e\right] = e, \end{aligned}$$

we have ${\mathbb {E}}_{{{\mathcal {D}}}}\left[ g^k\right] \overset{(14)}{=} \frac{1}{n}{\varvec{\nabla }}{} \mathbf{F}(x^k) e\overset{(5)}{=} \nabla f(x^k)$, and hence, $g^k$ is an unbiased estimate of the gradient. If (15) holds, we say that $\theta _{{\mathbf {S}}_k}$ is a *bias-correcting random variable* and ${\mathbf {S}}^k$ is an *unbiased sketch.* Our new *JacSketch* method is method (2) with $g^k$ computed via (13) and the Jacobian estimate updated via (11). This method is formalized in Sect. 2 as Algorithm 1.

This strategy indeed works, as we show in detail in this paper. Under appropriate conditions (on the stepsize $\alpha$, properties of *f* and randomness behind the sketch matrices ${\mathbf {S}}_k$ and so on), the variance of $g^k$ diminishes to zero (e.g., see Lemma 6), which means that JacSketch is a variance-reduced method. We perform an analysis for smooth and strongly convex functions *f*, and obtain a linear convergence result (Theorem 1). We summarize our complexity results in detail in Sect. 1.5.

### SAGA as a special case of JacSketch

Of particular importance in this paper are *minibatch sketches*, which are sketches of the form ${\mathbf {S}}_k = \mathbf{I}_{S_k}$, where $S_k$ is a random subset of [*n*], and $\mathbf{I}_{S_k}$ is a random column submatrix of the $n\times n$ identity matrix with columns indexed by $S_k$. For minibatch sketches, JacSketch corresponds to minibatch variants of SAGA. Indeed, in this case, and if $\mathbf{W}=\mathrm{Diag}(w_1,\ldots ,w_n)$, we have ${\varvec{\Pi }}_{{\mathbf {S}}_k} e= e_{S_k}$, where $e_{S} = \sum _{i\in S} e_i$ (see Lemma 7). Therefore,

16 $$\begin{aligned} g^{k} = \frac{1}{n} \mathbf{J}^k e+ \frac{\theta _{{\mathbf {S}}_k}}{n} \sum _{i\in S_k} \left( \nabla f_i(x^k)- \mathbf{J}^k_{:i}\right) . \end{aligned}$$

In view of (11), and since ${\varvec{\Pi }}_{{\mathbf {S}}_k} = \mathbf{I}_{S_k} \mathbf{I}_{S_k}^\top$ (see Lemma 7), the Jacobian estimate gets updated as follows

17 $$\begin{aligned} \mathbf{J}^{k+1}_{:i} = {\left\{ \begin{array}{ll} \mathbf{J}^k_{:i} &{} \quad i\notin S_k,\\ \nabla f_i(x^k)&{} \quad i\in S_k. \end{array}\right. } \end{aligned}$$

Standard uniform SAGA is obtained by setting $S_k = \{i\}$ with probability 1/*n* for each $i\in [n]$, and letting $\theta _{{\mathbf {S}}_k} \equiv n$. SAGA with arbitrary probabilities is obtained by instead choosing $S_k = \{i\}$ with probability $p_i>0$ for each $i\in [n]$, and letting $\theta _{{\mathbf {S}}_k} \equiv \tfrac{1}{p_i}$. However, virtually all minibatching and importance sampling strategies can be treated as special cases of our general approach.

The theory we develop answers the open questions raised earlier. In particular, we answer the conjecture of Schmidt et al. [30] about the rate of SAGA with importance sampling in the affirmative. In particular, we establish the iteration complexity $(n+ \frac{4\bar{L}}{ \mu } ) \log \tfrac{1}{\epsilon }.$ This complexity is obtained for *different* importance sampling distributions that have not been proposed in the current literature for SAGA. In order to achieve this, we develop a new analysis technique which makes use of a *stochastic Lyapunov function* (see Sect. 5). That is, our Lyapunov function has a random element which is independent of the randomness inherited from the iterates of the method. This is unlike any other Lyapunov function used in the analysis of stochastic methods we are aware of. Further, we prove that SAGA converges with any initial matrix $\mathbf{J}^0$ in place of the matrix of gradients of functions $f_i$ at the starting point. We also show that our results give better rates for minibatch SAGA than are currently known, even for uniform minibatch strategies. We also allow for a family of completely new uniform minibatching strategies which were not considered in connection with SAGA before, and consider also SAGA with importance sampling for minibatches^4^ (based on a partition of [*n*]). Lastly, as a special case, our method recovers standard gradient descent, together with the sharp iteration complexity of $\frac{4 L}{\mu }\log \tfrac{1}{\epsilon }$.

Our general approach also enables a novel *reduced memory* variant of SAGA as a special case. Let ${\mathbf {S}}_k=e_{S_k}$, and choose $\mathbf{W}= \mathbf{I}.$ Since ${\varvec{\Pi }}_{{\mathbf {S}}_k} e= e_{S_k}$, the formula for $g^k$ is the same as in the case of SAGA, and is given by (16). What is notably different about this sketch (compared to $\mathbf{I}_{S_k}$) is that, since ${\varvec{\Pi }}_{e_{S_k}} = \frac{1}{|S_k|}e_{S_k} e_{S_k}^\top ,$ the update of the Jacobian estimate is given by

$$\begin{aligned} \mathbf{J}^{k+1} \overset{(11)}{=} \mathbf{J}^{k} -\frac{1}{|S_k|} \sum _{i \in S_k}\left( \mathbf{J}^{k}_{:i} -\nabla f_i(x^k)\right) e_{S_k}^\top . \end{aligned}$$

Thus, *the same update is applied to all the columns of* $\mathbf{J}^k$ *that belong to* $S_k$. Equivalently, this update can be written as

18 $$\begin{aligned} \mathbf{J}^{k+1}_{:j} = {\left\{ \begin{array}{ll} \frac{1}{|S_k|} \sum \nolimits _{i\in S_k} \nabla f_{i}(x^k) &{} \text{ if }\quad j \in S_k,\\ \mathbf{J}^{k}_{:j} &{} \text{ if }\quad j \notin S_k. \end{array}\right. } \end{aligned}$$

In particular, if $S_k$ only ever picks sets which correspond to a partition of [*n*], and we initialize $\mathbf{J}^0$ so that all the columns belonging to the same partition are the same, then they will be the same within in each partition for all *k*. In such a case, we do not need to maintain all the identical copies. Instead, we can update and use a condensed/compressed version of the Jacobian, with one column per partition set only, to reduce the total memory usage. This method, with non-uniform probabilities, is analyzed in our framework in Sect. 5.6.

**Table 1:** Special cases of our JacSketch method, and the associated iteration complexity

ID	Method	Sketch ${\mathbf {S}}\in {\mathbb {R}}^{n\times \tau }$	Iteration complexity ($\times \log \tfrac{1}{\epsilon }$)	Reference
		$$\mathbf{W}\succ 0$$		
1	JacSketch	Any unbiased	$\max \left\{ \frac{4{{\mathcal {L}}}_1}{\mu }, \, \frac{1}{\kappa } + \frac{4\rho {{\mathcal {L}}}_2}{\kappa \mu n^2 } \right\}$	Theorem 1
		Any		
2	JacSketch	$\mathbf{I}_S$	$\max _{C \in \mathrm{supp}(S)} \left( \frac{1}{p_C}+ \frac{\tau }{n p_C }\frac{ 4 L_{C}}{\mu } \right)$	Theorem 6
	(Any probabilities for $\tau$—partition)	$\mathbf{I}$		
3	Gradient descent	$\mathbf{I}$	$\tfrac{4 L}{\mu }$	Theorems 1 and 6
		$\mathbf{I}$		Sections 4.6 and 5.6
4	SAGA	$\mathbf{I}_S$	$n+ \frac{4 L_{\max }}{\mu }$	Theorems 1 and 6
	(Uniform sampling)	$\mathbf{I}$		Sections 4.6 and 5.6
5	SAGA	$\mathbf{I}_S$	$n+ \frac{4 \bar{L}}{\mu }$	Theorem 6
	(Importance sampling)	$\mathbf{I}$		(129)
6	Minibatch SAGA	$\mathbf{I}_S$	$\max \left\{ \frac{4 L^{{{\mathcal {G}}}}_{\max }}{\mu }, \,\frac{n}{\tau } + \frac{4 \rho }{\mu n}\max _{i} \left( \frac{L_i}{w_i}\right) \right\}$	Theorem 1
	($\tau$—uniform sampling)	$\mathrm{Diag}(w_i)$		(100)
7	Minibatch SAGA	$\mathbf{I}_S$	$\max \left\{ \frac{4 L^{{{\mathcal {G}}}}_{\max }}{\mu }, \,\frac{n}{\tau } + \frac{n-\tau }{(n-1)\tau } \frac{4 L_{\max }}{\mu } \right\}$	Theorem 1
	($\tau$—nice sampling)	$\mathbf{I}$		(101)
8	Minibatch SAGA	$\mathbf{I}_S$	$\max \left\{ \frac{4 L^{{{\mathcal {G}}}}_{\max }}{\mu }, \, \frac{n}{\tau } + \frac{n-\tau }{n \tau } \frac{4 (\bar{L}+L_{\max })}{\mu } \right\}$	Theorem 1
	($\tau$—nice sampling)	$\mathrm{Diag}(L_i)$		(102)
9	Minibatch SAGA	$\mathbf{I}_S$	$\frac{n}{\tau } + \frac{4 L_{\max }}{\mu }$	Theorem 1
	($\tau$—partition sampling)	$\mathbf{I}$		(103)
10	Minibatch SAGA	$\mathrm{Diag}(L_i)$	$\frac{n}{\tau } + \frac{4 \max _{C\in \mathrm{supp}(S)} \frac{1}{ \tau }\sum _{i\in C} L_i}{\mu }$	Theorem 1
	($\tau$—partition sampling)	$\mathbf{I}_S$		(104)
11	Minibatch SAGA	$\mathbf{I}_S$	$\frac{n}{\tau }+ \frac{4 \frac{1}{|\mathrm{supp}(S)|}\sum _{C\in \mathrm{supp}(S)} L_C}{\mu }$	Theorem 6
	(Importance $\tau$—partition sampling)	$\mathbf{I}$		(131)

All methods converge linearly. In the iteration complexity column we list the number of iterations sufficient to obtain an $\epsilon$ accurate solution, ignoring a $\log \tfrac{1}{\epsilon }$ factor

### Summary of complexity results

All convergence results obtained in this paper are summarized in Table 1.

Our convergence results depend on several constants which we will now briefly introduce. The precise definitions can be found in the main text. For $\emptyset \ne C\subseteq [n]=\{1,2,\ldots ,n\}$, define $f_C(x) \overset{\text {def}}{=}\frac{1}{|C|}\sum _{i\in C} f_i(x)$. We assume $f_C$ is $L_C$—smooth.^5^ We let $L_i=L_{\{i\}}$, $L = L_{[n]}$, $L_{\max } = \max _i L_i$ and $\bar{L}=\tfrac{1}{n}\sum _i L_i$. Note that $L_i \le L_{\max }$, $\bar{L} \le L_{\max } \le n \bar{L}$, $L_C \le \tfrac{1}{|C|}\sum _{i\in C} L_i$ and $L \le \bar{L}$. For a sampling^6^$S\subseteq [n]$, we let $\mathrm{supp}(S) = \{C \subseteq [n] \;:\; {\mathbb {P}}\left[ S = C\right] >0\}$. That is, the support of a sampling are all the sets which are selected by this sampling with positive probability. Finally, $L^{{{\mathcal {G}}}}_{\max }= \max _i \tfrac{1}{c_1}\sum _{C \in \mathrm{supp}(S), i \in C} L_C$, where $c_1$ is the cardinality of the set $\{ C \;:\; C \in \mathrm{supp}(S), i \in C\}$ (which is assumed to be the same for all *i*). So, $L^{{{\mathcal {G}}}}_{\max }$ is the maximum over *i* of averages of values $L_C$ for those sets *C* which are picked by *S* with positive probability and which contain *i*. Clearly, $L^{{{\mathcal {G}}}}_{\max }\le L_{\max }$ (see Theorem 3).

*General theorem.* Theorem 1 is our most general result, allowing for any(unbiased) sketch ${\mathbf {S}}$ (see (15)), and any weight matrix $\mathbf{W}\succ 0$. The resulting iteration complexity given by this theorem is

$$\begin{aligned} \max \left\{ \frac{4{{\mathcal {L}}}_1}{\mu }, \, \frac{1}{\kappa } + \frac{4\rho {{\mathcal {L}}}_2}{\kappa \mu n^2 } \right\} \times \log \left( \frac{1}{\epsilon }\right) , \end{aligned}$$

and is also presented in the first row of Table 1. This result depends on two *expected smoothness* constants ${{\mathcal {L}}}_1$ (measuring the expected smoothness of the stochastic gradient of our stochastic reformulation; see Assumption 3.1) and ${{\mathcal {L}}}_2$ (measuring the expected smoothness of the Jacobian; see Assumption 3.2). The complexity also depends on the *stochastic contraction number* $\kappa$ (see (48)) and the *sketch residual* $\rho$ (see (37) and (55)). We devote considerable effort to give simple formulas for these constants under some specialized settings (for special combinations of sketches ${\mathbf {S}}$ and weight matrices $\mathbf{W}$). In fact, the entire Sect. 4 is devoted to this. In particular, all rows of Table 1 where the last column mentions Theorem 1 arise as special cases of the general iteration complexity in the first row.

- *Gradient descent* As a starting point, in row 3 we highlight that one can recover gradient descent as a special case of JacSketch with the choice ${\mathbf {S}}= \mathbf{I}$ (with probability 1) and $\mathbf{W}=\mathbf{I}$. We get the rate $\tfrac{4L}{\mu } \log \tfrac{1}{\epsilon }$, which is tight.
- *SAGA with uniform sampling* Let us now focus on a slightly more interesting special case: row 4. We see that SAGA with uniform probabilities appears as a special case, and enjoys the rate $\left( n+ \tfrac{4L_{\max }}{\mu }\right) \log \tfrac{1}{\epsilon }$, recovering an existing result.
- *SAGA with importance sampling* Unfortunately, the generality of Theorem 1 comes at a cost: we are not able to obtain an importance sampling version of SAGA as a special case which would have a better iteration complexity than uniform SAGA. This will be remedied by our second complexity theorem, which we shall discuss later below.
- *Minibatch SAGA* Rows 6–11 correspond to minibatch versions of SAGA. In particular, row 6 contains a general statement (albeit still a special case of the statement in row 1), covering virtually all minibatch strategies. Rows 7–11 specialize this result to two particular minibatch sketches (i.e., ${\mathbf {S}}=\mathbf{I}_S$), each with two choices of $\mathbf{W}$. The first sketch corresponds to samplings *S* which choose from among all subsets of [*n*] uniformly at random. This sampling is known in the literature as $\tau$-nice sampling [22, 25]. The second sketch corresponds to *S* being a $\tau$—partition sampling. This sampling picks uniformly at random subsets of [*n*] which form a partition of [*n*], and are all of cardinality $\tau$. The complexities in rows 7 and 8 are comparable (each can be slightly better than the other, depending on the values of the smoothness constants $\{L_i\}$). On the other hand, in the case of $\tau$—partition, the choice $\mathbf{W}=\mathrm{Diag}(L_i)$ is better than $\mathbf{W}= \mathbf{I}$: the complexity in row 10 is better than that in row 9 because $\max _{C\in \mathrm{supp}(S)} \frac{1}{\tau } \sum _{i\in C} L_i \le L_{\max }.$
- *Optimal minibatch size for SAGA* Our analysis for mini-batch SAGA also gives the first iteration complexities that interpolate between the $(n+\frac{4L_{\max }}{\mu })\log \tfrac{1}{\epsilon }$ complexity of SAGA and the $\frac{4L}{\mu }\log \tfrac{1}{\epsilon }$ complexity of gradient descent, as $\tau$ increases from 1 to *n*. Indeed, consider the complexity in rows 7 and 8 for $\tau =1$ and $\tau =n.$ Our iteration complexity of mini-batch SAGA is the first result that is precise enough to inform an optimal mini-batch size (see Sect. 6.2). In contrast, the previous best complexity result for mini-batch SAGA [14] interpolates between $(n+\frac{4L_{\max }}{\mu }) \log \tfrac{1}{\epsilon }$ and $\frac{4L_{\max }}{\mu } \log \tfrac{1}{\epsilon }$ as $\tau$ increases from 1 to *n*, and thus is not precise enough as to inform the best minibatch size. We make a more detailed comparison between our results and [14] in Sect. 4.7.

*Specialized theorem* We now move to the second main complexity result of our paper: Theorem 6. The general complexity statement is listed in row 2 of Table 1:

19 $$\begin{aligned} \max _{C\in \mathrm{supp}(S)} \left( \frac{1}{p_C} + \frac{\tau }{np_C} \frac{4 L_C}{\mu }\right) \times \log \left( \frac{1}{\epsilon }\right) , \end{aligned}$$

where $p_C = {\mathbb {P}}\left[ S=C\right]$. This theorem is a refined result specialized to minibatch sketches (${\mathbf {S}}=\mathbf{I}_S$) with $\tau$—partition samplings *S*. This is a sampling which picks subsets of [*n*] of size $\tau$ forming a partition of [*n*], uniformly at random. This theorem also includes gradient descent as special case since when $S=[n]$ with probability 1 (hence, $p_{[n]}=1$) we have that $\tau =n$ and $L_{[n]} = L$. Hence, (19) specializes to $\frac{4 L}{\mu } \log \frac{1}{\epsilon }$. But more importantly, our focus on $\tau$—partition samplings enables us to provide stronger iteration complexity guarantees for non-uniform probabilities.

- *SAGA with importance sampling* The first remarkable special case of (19) is summarized in row 5, and corresponds to SAGA with importance sampling. The complexity obtained, $(n+ \tfrac{4\bar{L}}{\mu }) \log \tfrac{1}{\epsilon }$, answers a conjecture of Schmidt et al. [30] in the affirmative. In this case, the support of *S* are the singletons $\{1\}$, $\{2\}, \ldots , \{n\}$, $p_{\{i\}} = p_i$ for all *i*, $\tau =1$ and $L_{\{i\}} = L_i$. Optimizing the complexity bound over the probabilities $p_1,\ldots ,p_n$, we obtain the importance sampling $p_i = \frac{\mu n + 4\tau L_i}{\sum _j \mu n + 4\tau L_j}.$
- *Minibatch SAGA with importance sampling* In row 11 we state the complexity for a minibatch SAGA method with importance sampling. This is the first result for this method in the literature. Note that by comparing rows 4 and 10, we can conclude that the complexity of minibatch SAGA with importance sampling is better than for minibatch SAGA with uniform probabilities. Indeed, this is because^7^
  20 $$\begin{aligned} \frac{1}{|\mathrm{supp}(S)|}\sum _{C\in \mathrm{supp}(S)} L_C \le \bar{L} \le \max _{C\in \mathrm{supp}(S)} \frac{1}{\tau } \sum _{i\in C} L_i. \end{aligned}$$

### Outline of the paper

We present an alternative narrative motivating the development of JacSketch in Sect. 2. This narrative is based on a novel technical tool which we call *controlled stochastic optimization reformulations* of problem (1). We then develop a general convergence theory of JacSketch in Sect. 3. This theory admits practically any sketches ${\mathbf {S}}$ (including minibatch sketches mentioned in the introduction) and weight matrices $\mathbf{W}$. The main result in this section is Theorem 1. In Sect. 4 we specialize the general results to minibatch sketches. Here we also compute the various constants appearing in the general complexity result for JacSketch for specific classes of minibatch samplings. In Sect. 5 we develop an alternative theory for JacSketch, one based on a novel *stochastic Lyapunov function*. The main result in this section is Theorem 6. Computational experiments are included in Sect. 6.

### Notation

We will introduce notation when and as needed. If the reader would like to recall any notation, for ease of reference we have a notation glossary in Sect. 1. As a general rule, all matrices are written in upper-case bold letters. By $\log t$ we refer to the natural logarithm of *t*.

## Controlled stochastic reformulations

In this section we provide an alternative narrative behind the development of JacSketch; one through the lens of what we call *controlled stochastic reformulations*.

We design our family of methods so that two keys properties are satisfied, namely *unbiasedness*, ${\mathbb {E}}\left[ g^k\right] = \nabla f(x^k),$ and *diminishing variance:* ${\mathbb {E}}\left[ \left\| g^k - \nabla f(x^k) \right\| _2^2\right] \longrightarrow 0$ as $x^k \rightarrow x^*$. These are both favoured statistical properties. Moreover, currently only methods that have diminishing variance exhibt fast linear convergence (exponential decay of the error) on strongly convex problems. On the other hand, unbiasedness is not necessary for a fast method in practice since several biased stochastic gradient methods such as SAG [29] perform well in practice. Still, the absence of bias greatly facilitates the analysis of JacSketch.

### Stochastic reformulation using sketching

It will be useful to formalize the condition mentioned in Sect. 1.3 which leads to $g^k$ being an unbiased estimator of the gradient.

#### Assumption 2.1

(*Unbiased sketch*) Let $\mathbf{W}\succ 0$ be a weighting matrix and let ${{\mathcal {D}}}$ be the distribution from which the sketch matrices ${\mathbf {S}}$ are drawn. There exists a random variable $\theta _{{\mathbf {S}}}$ such that

21 $$\begin{aligned} {\mathbb {E}}_{{{\mathcal {D}}}}\left[ \theta _{{\mathbf {S}}} {\varvec{\Pi }}_{\mathbf {S}}\right] e= e. \end{aligned}$$

When this assumption is satisfied, we say that $({\mathbf {S}}, \theta _{\mathbf {S}}, \mathbf{W})$ constitutes an “unbiased sketch”, and we call $\theta _{{\mathbf {S}}}$ the bias-correcting random variable. When the triple is obvious from the context, sometimes we shall simply say that ${\mathbf {S}}$ is an unbiased sketch.

The first key insight of this section is that besides producing unbiased estimators of the gradient, unbiased sketches produce *unbiased estimators of the loss function* as well. Indeed, by simply observing that $f(x) = \frac{1}{n}\left\langle F(x),e\right\rangle$, we get

$$\begin{aligned} f(x) \overset{(1)}{=} \frac{1}{n}\sum _{i=1}^n f_i(x)= & {} \frac{1}{n}\left\langle F(x),e\right\rangle \overset{(21)}{=} \frac{1}{n}\left\langle F(x),{\mathbb {E}}_{{{\mathcal {D}}}}\left[ \theta _{{\mathbf {S}}} {\varvec{\Pi }}_{\mathbf {S}}e\right] \right\rangle \\= & {} {\mathbb {E}}_{{{\mathcal {D}}}}\left[ \frac{1}{n}\left\langle F(x),\theta _{{\mathbf {S}}} {\varvec{\Pi }}_{\mathbf {S}}e\right\rangle \right] . \end{aligned}$$

In other words, we can rewrite the finite-sum optimization problem (1) as an equivalent stochastic optimization problem where the randomness comes from ${{\mathcal {D}}}$ rather than from the representation-specific uniform distribution over the *n* loss functions:

22 $$\begin{aligned} \min _{x \in {\mathbb {R}}^d} f(x) = {\mathbb {E}}_{{{\mathcal {D}}}}\left[ f_{{\mathbf {S}}}(x)\right] , \qquad \text {where} \qquad f_{{\mathbf {S}}}(x) \overset{\text {def}}{=}\frac{\theta _{{\mathbf {S}}}}{n}\left\langle F(x), {\varvec{\Pi }}_{\mathbf {S}}e\right\rangle . \end{aligned}$$

The stochastic optimization problem (22) is a *stochastic reformulation* of the original problem (1). Further, the stochastic gradient of this reformulation is given by

23 $$\begin{aligned} \nabla f_{{\mathbf {S}}}(x) = \frac{\theta _{{\mathbf {S}}}}{n}{\varvec{\nabla }}{} \mathbf{F}(x) {\varvec{\Pi }}_{\mathbf {S}}e. \end{aligned}$$

With these simple observations, our options at designing stochastic gradient-type algorithms for (1) have suddenly broadened dramatically. Indeed, we can now solve the problem, at least in principle, by applying SGD to any stochastic reformulation:

24 $$\begin{aligned} x^{k+1} = x^k - \alpha \nabla f_{{\mathbf {S}}_k} (x^k). \end{aligned}$$

But now we have a parameter to play with, namely, the distribution of ${\mathbf {S}}$. The choice of this parameter will influence both the iteration complexity of the resulting method as well as the cost of each iteration. We now give a few examples of possible choices of ${{\mathcal {D}}}$ to illustrate this.

#### Example 1

(gradient descent) Let ${\mathbf {S}}$ be equal to $\mathbf{I}$ (or any other $n\times n$ invertible matrix) with probability 1 and let $\mathbf{W}\succ 0$ be chosen arbitrarily. Then $\theta _{{\mathbf {S}}} \equiv 1$ is bias-correcting since

$$\begin{aligned} {\mathbb {E}}_{{{\mathcal {D}}}}\left[ \theta _{\mathbf {S}}{\varvec{\Pi }}_{\mathbf {S}}e\right] ={\varvec{\Pi }}_{{\mathbf {S}}} e\overset{(12)}{=} {\mathbf {S}}({\mathbf {S}}^\top \mathbf{W}{\mathbf {S}})^{\dagger } {\mathbf {S}}^\top \mathbf{W}e= {\mathbf {S}}{\mathbf {S}}^{-1}\mathbf{W}^{-1} ({\mathbf {S}}^{\top })^{-1} {\mathbf {S}}^\top \mathbf{W}e= \mathbf{I}e= e. \end{aligned}$$

With this setup, the SGD method (24) becomes *gradient descent:*

25 $$\begin{aligned} x^{k+1} = x^k - \alpha \nabla f_{{\mathbf {S}}_k} (x^k) \overset{(5)+(23)}{=} x^k - \alpha \nabla f (x^k). \end{aligned}$$

#### Example 2

(SGD with non-uniform sampling) Let ${\mathbf {S}}=e_i$ (unit basis vector in ${\mathbb {R}}^n$) with probability $p_i>0$ and let $\mathbf{W}= \mathbf{I}$. Then $\theta _{e_i} = 1/p_i$ is bias-correcting since

$$\begin{aligned} {\mathbb {E}}_{{{\mathcal {D}}}}\left[ \theta _{\mathbf {S}}{\varvec{\Pi }}_{\mathbf {S}}e\right] \overset{(12)}{=} \sum _{i=1}^n p_i \frac{1}{p_i} e_i(e_i^\top e_i)^{-1} e_i^\top e=\sum _{i=1}^n e_i e_i^\top e= \mathbf{I}e= e. \end{aligned}$$

Let $S_k= \{i_k\}$ be picked at iteration *k*. Then the SGD method (24) becomes *SGD with non-uniform sampling:*

26 $$\begin{aligned} x^{k+1} = x^k - \alpha \nabla f_{{\mathbf {S}}_k} (x^k) \overset{(23)}{=} x^k - \frac{\alpha }{n p_{i_k}} \nabla f_{i_k} (x^k). \end{aligned}$$

Note that with this setup, and when $p_i=1/n$ for all *i*, the stochastic reformulation is identical to the original finite-sum problem. This is the case because $f_{e_i}(x) = f_i(x)$.

#### Example 3

(minibatch SGD) Let ${\mathbf {S}}=e_{S} = \sum _{i\in S}e_i$, where $S=C\subseteq [n]$ with probability $p_C$. Let $\mathbf{W}= \mathbf{I}$. Assume that the cardinality of the set $\{C \subseteq [n]\;:\;C\in \mathrm{supp}(S), \; i\in C \}$ does not depend on *i* (and is equal to $c_1>0$). Then $\theta _{e_S} = 1/(c_1 p_S)$ is bias-correcting since

$$\begin{aligned} {\mathbb {E}}_{{{\mathcal {D}}}}\left[ \theta _{\mathbf {S}}{\varvec{\Pi }}_{\mathbf {S}}e\right] \overset{(12)}{=} \sum _{C\in \mathrm{supp}(S)} p_C \frac{1}{c_1 p_C} e_C(\underbrace{e_C^\top e_C}_{|C|})^{-1} \underbrace{e_C^\top e}_{|C|} = \sum _{C\in \mathrm{supp}(S)} \frac{1}{c_1} e_C = e. \end{aligned}$$

Note that ${\varvec{\Pi }}_{e_S}e= e_S$. Assume that set $S_k$ is picked in iteration *k*. Then the SGD method (24) becomes *minibatch SGD with non-uniform sampling:*

27 $$\begin{aligned} x^{k+1} = x^k - \alpha \nabla f_{{\mathbf {S}}_k} (x^k) \overset{(23)}{=} x^k - \frac{\alpha }{n c_1}\sum _{i\in S_k}\frac{1}{p_{S_k}} \nabla f_i (x^k). \end{aligned}$$

Finally, note that gradient descent (25) is a special case of (27) if we set $p_{[n]} = 1$ and $p_{C}=0$ for all other subsets *C* of [*n*]. Likewise, SGD with non-uniform probabilities (26) is a special case of (27) if we set $p_{ \{i\} } = p_i>0$ for all *i* and $p_{C}=0$ for all other subsets *C* of [*n*].

### The controlled stochastic reformulation

Though SGD applied to the stochastic reformulation can generate several known algorithms in special cases, there is no reason to believe that the gradient estimates $g^k$ will have diminishing variance (excluding the extreme case such as gradient descent). Here we handle this issue using *control variates*, a commonly used tool to reduce variance in Monte Carlo methods [13] and introduced in [35] for designing variance reduced stochastic gradient algorithm.

Given a random function $z_{{\mathbf {S}}}(x)$, we introduce the *controlled stochastic reformulation*:

28 $$\begin{aligned} \min _{x \in {\mathbb {R}}^d} f(x) = {\mathbb {E}}_{{{\mathcal {D}}}}\left[ f_{{\mathbf {S}},z}(x)\right] , \quad \text {where} \quad f_{{\mathbf {S}},z}(x) \overset{\text {def}}{=}f_{{\mathbf {S}}}(x) -z_{{\mathbf {S}}}(x) + {\mathbb {E}}_{{{\mathcal {D}}}}\left[ z_{{\mathbf {S}}}(x)\right] .\nonumber \\ \end{aligned}$$

Since

29 $$\begin{aligned} \nabla f_{{\mathbf {S}},z}(x) \overset{\text {def}}{=}\nabla f_{{\mathbf {S}}}(x) - \nabla z_{{\mathbf {S}}}(x) + {\mathbb {E}}_{{{\mathcal {D}}}}\left[ \nabla z_{{\mathbf {S}}}(x)\right] \end{aligned}$$

is an unbiased estimator of the gradient $\nabla f(x)$, we can apply SGD to the controlled stochastic reformulation instead, which leads to the method

$$\begin{aligned} x^{k+1} = x^k -\alpha ( \nabla f_{{\mathbf {S}}_k}(x) - \nabla z_{{\mathbf {S}}_k}(x) + {\mathbb {E}}_{{{\mathcal {D}}}}\left[ \nabla z_{{\mathbf {S}}}(x)\right] ). \end{aligned}$$

Reformulation (22) and method (24) is recovered as a special case with the choice $z_{\mathbf {S}}(x) \equiv 0$. However, we now have the extra freedom to choose $z_{{\mathbf {S}}}(x)$ so as to control the variance of this stochastic gradient. In particular, if $\nabla z_{{\mathbf {S}}}(x)$ and $\nabla f_{{\mathbf {S}}}(x)$ are sufficiently correlated, then (29) will have a smaller variance than $\nabla f_{{\mathbf {S}}}(x).$ For this reason, we choose a linear model for $z_{{\mathbf {S}}}(x)$ that mimicks the stochastic function $f_{{\mathbf {S}}}(x).$

Let $\mathbf{J}\in {\mathbb {R}}^{d \times n}$ be a matrix of parameters of the following linear model

30 $$\begin{aligned} z_{{\mathbf {S}}}(x) \overset{\text {def}}{=}\frac{\theta _{{\mathbf {S}}}}{n} \left\langle \mathbf{J}^\top x, {\varvec{\Pi }}_{\mathbf {S}}e\right\rangle , \quad \nabla z_{{\mathbf {S}}}(x) = \frac{\theta _{{\mathbf {S}}}}{n} \mathbf{J}\, {\varvec{\Pi }}_{\mathbf {S}}e. \end{aligned}$$

Note that this linear model has the same structure as $f_{{\mathbf {S}}}(x)$ in (22) except that *F*(*x*) has been replaced by the linear function $\mathbf{J}^\top x$.^8^ If ${\mathbf {S}}$ is an unbiased sketch (see (21)), we get ${\mathbb {E}}_{{{\mathcal {D}}}}\left[ \nabla z_{{\mathbf {S}}}(x)\right] = \frac{1}{n} \mathbf{J}e$, which plugged into (28) and (29) together with the definition (22) of $f_{\mathbf {S}}$ gives the following unbiased estimate of *f*(*x*) and $\nabla f(x)$:

31 $$\begin{aligned} f_{{\mathbf {S}},\mathbf{J}}(x) \overset{\text {def}}{=}f_{{\mathbf {S}},z}(x) = \frac{\theta _{{\mathbf {S}}}}{n} \left\langle F(x)- \mathbf{J}^\top x, {\varvec{\Pi }}_{\mathbf {S}}e\right\rangle + \frac{1}{n}\left\langle \mathbf{J}^\top x, e\right\rangle , \end{aligned}$$

and

32 $$\begin{aligned} \nabla f_{{\mathbf {S}},\mathbf{J}}(x) \overset{\text {def}}{=}\nabla f_{{\mathbf {S}},z}(x) = \frac{\theta _{{\mathbf {S}}}}{n} ({\varvec{\nabla }}{} \mathbf{F}(x)-\mathbf{J}) {\varvec{\Pi }}_{\mathbf {S}}e+ \frac{1}{n} \mathbf{J}e. \end{aligned}$$

We collect this observation that (32) is unbiased in the following lemma for future reference.

#### Lemma 1

If ${\mathbf {S}}$ is an unbiased sketch (see Definition 2.1), then

33 $$\begin{aligned} {\mathbb {E}}_{{{\mathcal {D}}}}\left[ \nabla f_{{\mathbf {S}},\mathbf{J}}(x)\right] = \nabla f(x), \end{aligned}$$

for every $\mathbf{J}\in {\mathbb {R}}^{d \times n}$ and $x\in {\mathbb {R}}^d$. That is, (32) is an unbiased estimate of the gradient (1).

Now it remains to choose the matrix $\mathbf{J}$, which we do by minimizing the variance of our gradient estimate.

### The Jacobian estimate, variance reduction and the sketch residual

Since (32) gives an unbiased estimator of $\nabla f(x)$ for all $\mathbf{J}\in {\mathbb {R}}^{d \times n}$, we can attempt to choose $\mathbf{J}$ that minimizes its variance. Minimizing the variance of (32) in terms of $\mathbf{J}$ will, for all sketching matrices of interest, lead to $\mathbf{J}= {\varvec{\nabla }}{} \mathbf{F}(x).$ This follows because

34 $$\begin{aligned}&{\mathbb {E}}_{{{\mathcal {D}}}}\left[ \left\| \nabla f_{{\mathbf {S}},\mathbf{J}}(x) - \nabla f(x) \right\| _2^2\right] \nonumber \\&\quad \overset{(32)}{=} {\mathbb {E}}_{{{\mathcal {D}}}}\left[ \left\| \frac{1}{n} \mathbf{J}( \mathbf{I}-\theta _{{\mathbf {S}}} {\varvec{\Pi }}_{\mathbf {S}})e-\frac{1}{n}{\varvec{\nabla }}{} \mathbf{F}(x)( \mathbf{I}-\theta _{{\mathbf {S}}} {\varvec{\Pi }}_{\mathbf {S}})e \right\| _2^2\right] \nonumber \\&\quad = \frac{1}{n^2}{\mathbb {E}}_{{{\mathcal {D}}}}\left[ \left\| (\mathbf{J}-{\varvec{\nabla }}{} \mathbf{F}(x))(\mathbf{I}-\theta _{{\mathbf {S}}} {\varvec{\Pi }}_{\mathbf {S}})e \right\| _2^2\right] \nonumber \\&\quad = \frac{1}{n^2}\text{ Tr }\left( (\mathbf{J}-{\varvec{\nabla }}{} \mathbf{F}(x))^\top (\mathbf{J}-{\varvec{\nabla }}{} \mathbf{F}(x)) \mathbf{B}\right) \nonumber \\&\quad = \frac{1}{n^2} \Vert \mathbf{J}-{\varvec{\nabla }}{} \mathbf{F}(x)\Vert _{\mathbf{B}}^2, \end{aligned}$$

where

35 $$\begin{aligned} \mathbf{B}&\overset{\text {def}}{=}&{\mathbb {E}}_{{{\mathcal {D}}}}\left[ ( \mathbf{I}-\theta _{{\mathbf {S}}} {\varvec{\Pi }}_{\mathbf {S}})ee^\top ( \mathbf{I}-\theta _{{\mathbf {S}}} {\varvec{\Pi }}_{\mathbf {S}}^\top )\right] \overset{(21)}{=} {\mathbb {E}}_{{{\mathcal {D}}}}\left[ \theta _{{\mathbf {S}}}^2 {\varvec{\Pi }}_{{\mathbf {S}}} ee^\top {\varvec{\Pi }}_{{\mathbf {S}}}^\top \right] - ee^\top \succeq 0,\nonumber \\ \end{aligned}$$

and we have used the weighted Frobenius norm with weight matrix $\mathbf{B}$ (see (10)).

For most distributions ${{\mathcal {D}}}$ of interest, the matrix $\mathbf{B}$ is positive definite.^9^ Letting $v_{\mathbf {S}}\overset{\text {def}}{=}( \mathbf{I}-\theta _{{\mathbf {S}}} {\varvec{\Pi }}_{\mathbf {S}})e$, we can bound the largest eigenvalue of matrix $\mathbf{B}$ via Jensen’s inequality as follows:

$$\begin{aligned} \lambda _{\max }(\mathbf{B}) \overset{(35)}{=} \lambda _{\max }({\mathbb {E}}_{{{\mathcal {D}}}}\left[ v_{\mathbf {S}}v_{\mathbf {S}}^\top \right] ) \le {\mathbb {E}}_{{{\mathcal {D}}}}\left[ \lambda _{\max }(v_{\mathbf {S}}v_{\mathbf {S}}^\top )\right] = {\mathbb {E}}_{{{\mathcal {D}}}}\left[ \Vert v_{\mathbf {S}}\Vert _2^2\right] . \end{aligned}$$

Combined with (34), we get the following bound on the variance of $\nabla f_{{\mathbf {S}},\mathbf{J}}$:

$$\begin{aligned} {\mathbb {E}}_{{{\mathcal {D}}}}\left[ \left\| \nabla f_{{\mathbf {S}},\mathbf{J}}(x) - \nabla f(x) \right\| _2^2\right] \le \frac{{\mathbb {E}}_{{{\mathcal {D}}}}\left[ \Vert v_{\mathbf {S}}\Vert _2^2\right] }{n^2} \Vert \mathbf{J}- {\varvec{\nabla }}{} \mathbf{F}(x)\Vert _\mathbf{I}^2. \end{aligned}$$

This suggests that the variance is low when $\mathbf{J}$ is close to the true Jacobian ${\varvec{\nabla }}{} \mathbf{F}(x)$, and when the second moment of $v_{\mathbf {S}}$ is small. If ${\mathbf {S}}$ is an unbiased sketch, then ${\mathbb {E}}_{{{\mathcal {D}}}}\left[ v_{\mathbf {S}}\right] =0$, and hence ${\mathbb {E}}_{{{\mathcal {D}}}}\left[ \Vert v_{\mathbf {S}}\Vert _2^2\right]$ is the variance of $v_{\mathbf {S}}$. So, the lower the variance of $\tfrac{1}{n}\theta _{\mathbf {S}}{\varvec{\Pi }}_{\mathbf {S}}e$ as an estimator of $\tfrac{1}{n}e$, the lower the variance of $\nabla f_{{\mathbf {S}},\mathbf{J}}(x)$ as an estimator of $\nabla f(x)$.

Let us now return to the identity (34) and its role in choosing $\mathbf{J}$. Minimizing the variance in a single step is overly ambitious, since it requires setting $\mathbf{J}= {\varvec{\nabla }}{} \mathbf{F}(x)$, which is costly. So instead, we propose to minimize (34) iteratively. But first, to make (34) more manageable, we upper-bound it using a norm defined by the weight matrix $\mathbf{W}$ as follows

36 $$\begin{aligned} \Vert \mathbf{J}-{\varvec{\nabla }}{} \mathbf{F}(x)\Vert _{\mathbf{B}}^2 \le \rho \,\Vert \mathbf{J}-{\varvec{\nabla }}{} \mathbf{F}(x) \Vert _{\mathbf{W}^{-1}}^2, \end{aligned}$$

where

37 $$\begin{aligned} \rho \overset{\text {def}}{=}\lambda _{\max }\left( \mathbf{W}^{1/2} \mathbf{B}\mathbf{W}^{1/2}\right) \ge 0 \end{aligned}$$

is the largest eigenvalue of $\mathbf{W}^{1/2} \mathbf{B}\mathbf{W}^{1/2}$. We refer to the constant $\rho$ as the *sketch residual*, and it is a key constant affecting the convergence rate of JacSketch as captured by Theorem 1. The sketch residual $\rho$ represents how much information is “lost” on average due to sketching and due to how well $\mathbf{W}^{-1}$ approximates $\mathbf{B}$. We develop formulae and estimates of the sketch residual for several specific sketches of interest in Sect. 4.5.

#### Example 4

(Zero sketch residual) Consider the setup from Example 1 (gradient descent). That is, let ${\mathbf {S}}$ be invertible with probability one and let $\theta _{{\mathbf {S}}}=1$ be the bias-reducing variable. Then ${\varvec{\Pi }}_{\mathbf {S}}e= e$ and hence $\mathbf{B}=0$, which means that $\rho =0$.

#### Example 5

(Large sketch residual) Consider the setup from Example 2 (SGD with non-uniform probabilities). That is, let ${\mathbf {S}}=e_i$ (unit basis vector in ${\mathbb {R}}^n$) with probability $p_i>0$ and let $\mathbf{W}= \mathbf{I}$. Then $\theta _{e_i} = 1/p_i$ is a bias-reducing variable, and it is easy to show that $\mathbf{B}= \mathrm{Diag}(1/p_1,\ldots ,1/p_n) - ee^\top$. If we choose $p_i=1/n$ for all *i*, then $\rho =n$.

We have switched from the $\mathbf{B}$ norm to a user-controlled $\mathbf{W}^{-1}$ norm because minimizing under the $\mathbf{B}$ norm will prove to be impractical because $\mathbf{B}$ is a dense matrix for most all practical sketches. With this norm change we now have the option to set $\mathbf{W}$ as a sparse matrix (e.g., the identity, or a diagonal matrix), as we explain in Remark 1 further down. However, the theory we develop allows for any symmetric positive definite matrix $\mathbf{W}$.

We can now minimize (36) iteratively by only using a single sketch of the true Jacobian at each iteration. Suppose we have a current estimate $\mathbf{J}^k$ of the true Jacobian and a sketch of the true Jacobian ${\varvec{\nabla }}{} \mathbf{F}(x^k) {\mathbf {S}}_k$. With this we can calculate an improved Jacobian estimate using a projection step

38 $$\begin{aligned} \mathbf{J}^{k+1}= \underset{\mathbf{J}\in {\mathbb {R}}^{d \times n}}{\arg } \underset{\mathbf{Y}\in {\mathbb {R}}^{ m \times \tau }}{\min \,} \frac{1}{2}\left\| \mathbf{J}- {\varvec{\nabla }}{} \mathbf{F}(x^k) \right\| _{\mathbf{W}^{-1}}^2 \quad \text{ subject } \text{ to } \quad \mathbf{J}= \mathbf{J}^{k} + \mathbf{Y}{\mathbf {S}}_k^\top \mathbf{W}, \end{aligned}$$

the solution of which, as it turns out, depends on ${\varvec{\nabla }}{} \mathbf{F}(x^k)$ through its sketch ${\varvec{\nabla }}{} \mathbf{F}(x^k) {\mathbf {S}}_k$ only. That is, we choose the next Jacobian estimate $\mathbf{J}^{k+1}$ as close as possible to the true Jacobian ${\varvec{\nabla }}{} \mathbf{F}(x^k)$ while restricted to a matrix subspace that passes through $\mathbf{J}^k$. Thus in light of (36), the variance is decreasing. The explicit solution to (38) is given by

39 $$\begin{aligned} \mathbf{J}^{k+1} = \mathbf{J}^{k} -(\mathbf{J}^{k}-{\varvec{\nabla }}{} \mathbf{F}(x^k)) {\varvec{\Pi }}_{{\mathbf {S}}_k}. \end{aligned}$$

See Lemma B.1 in the appendix of an extended preprint version of this paper [10] or Theorem 4.1 in [12] for the proof. Note that, as alluded to before, $\mathbf{J}^{k+1}$ depends on ${\varvec{\nabla }}{} \mathbf{F}(x^k)$ through its sketch only. Note that (39) updates the Jacobian estimate by re-using the sketch ${\varvec{\nabla }}{} \mathbf{F}(x^k) {\mathbf {S}}_k$ which we also use when calculating the stochastic gradient (32).

Note that (39) gives the same formula for $\mathbf{J}^{k+1}$ as (11) which we obtained by solving (9); i.e., by projecting $\mathbf{J}^k$ onto the solution set of (8). This is not a coincidence. In fact, the optimization problems (9) and (38) are mutually dual. This is also formally stated in Lemma B.1 in [10].

In the context of solving linear systems, this was observed in [11]. Therein, (9) is called the sketch-and-project method, whereas (38) is called the *constrain-and-approximate* problem. In this sense, the Jacobian sketching narrative we followed in Sect. 1.3 is dual to the Jacobian sketching narrative we are pursuing here.

#### Remark 1

(On the weight matrix and the cost) Loosely speaking, the denser the weighting matrix $\mathbf{W}$, the higher the computational cost for updating the Jacobian using (39). Indeed, the sparsity pattern of $\mathbf{W}$ controls how many elements of the previous Jacobian estimate $\mathbf{J}^k$ need to be updated. This can be seen by re-arranging (39) as

40 $$\begin{aligned} \mathbf{J}^{k+1} = \mathbf{J}^{k} + \mathbf{Y}_k {\mathbf {S}}_k^\top \mathbf{W}, \end{aligned}$$

where $\mathbf{Y}_k = ({\varvec{\nabla }}{} \mathbf{F}(x^k) {\mathbf {S}}_k-\mathbf{J}^{k} {\mathbf {S}}_k) ({\mathbf {S}}_k^\top \mathbf{W}{\mathbf {S}}_k)^{\dagger } \in {\mathbb {R}}^{d \times \tau }.$ Although we have no control over the sparsity of $\mathbf{Y}_k$, the matrix ${\mathbf {S}}_k^\top \mathbf{W}$ can be sparse when both ${\mathbf {S}}_k$ and $\mathbf{W}$ are sparse. This will be key in keeping the update (40) at a cost propotional to $d \times \tau$, as oppossed to $n\times d$ when $\mathbf{W}$ is dense. This is why we consider a diagonal matrix $\mathbf{W}= \mathrm{Diag}(w_1,\ldots , w_n)$ in all of the special complexity results in Table 1. While it is clear that some non-diagonal sparse matrices $\mathbf{W}$ could also be used, we leave such considerations to future work.

### JacSketch algorithm

Combining formula (32) for the stochastic gradient of the controlled stochastic reformulation with formula (39) for the update of the Jacobian estimate, we arrive at our JacSketch algorithm (Algorithm 1).
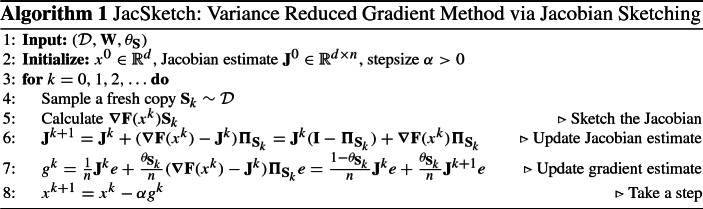


Typically, one should not implement the algorithm as presented above. The most efficient implementation of JacSketch will depend heavily on the structure of $\mathbf{W}$, distribution ${{\mathcal {D}}}$ and so on. For instance, in the special case of minibatch SAGA, as presented in Sect. 1.4, the update of the Jacobian (77) has a particularly simple form. That is, we maintain a single matrix $\mathbf{J}\in {\mathbb {R}}^{d\times n}$ and keep replacing its columns by the appropriate stochastic gradients, as computed. Moreover, in the case of linear predictors, as is well known, a much more memory-efficient implementation is possible. In particular, if $f_i(x) = \phi _i(a_i^\top x)$ for some loss function $\phi _i$ and a data vector $a_i\in {\mathbb {R}}^d$ and all *i*, then $\nabla f_i(x) = \phi _i'(a_i^\top x) a_i$, which means that the gradient always points in the same direction. In such a situation, it is sufficient to keep track of the scalar loss derivatives $\phi _i'(a_i^\top x)$ only. Similar comments can be made about the step (16) for computing the gradient estimate $g^k$.

### A window into biased estimates and SAG

We will now take a small detour from the main flow of the paper to develop an alternative viewpoint of Algorithm 1 and also make a bridge to biased methods such as SAG [29].

The simple observation that

41 $$\begin{aligned} \nabla f(x^k) = \frac{1}{n}{\varvec{\nabla }}{} \mathbf{F}(x^k)e, \end{aligned}$$

suggests that $\hat{g}^k = \frac{1}{n} \mathbf{J}^{k+1} e$, where $\mathbf{J}^{k+1} \approx {\varvec{\nabla }}{} \mathbf{F}(x^k)$ would give a good estimate of the gradient. To decrease the variance of $\hat{g}^k$, we can also use the same update of the Jacobian estimate (39) since

$$\begin{aligned} {\mathbb {E}}\left[ \left\| \hat{g}^k - \nabla f(x^k) \right\| _2^2\right]= & {} \frac{1}{n^2} {\mathbb {E}}\left[ \left\| ( \mathbf{J}^{k+1} -{\varvec{\nabla }}{} \mathbf{F}(x^k)) e \right\| _2^2\right] \\= & {} \frac{1}{n^2} {\mathbb {E}}\left[ \left\| ( \mathbf{J}^{k+1} -{\varvec{\nabla }}{} \mathbf{F}(x^{k})) \mathbf{W}^{-1/2} \mathbf{W}^{1/2} e \right\| _2^2\right] \\\le & {} \frac{e^\top \mathbf{W}e}{ n^2} {\mathbb {E}}\left[ \left\| \mathbf{J}^{k+1} -{\varvec{\nabla }}{} \mathbf{F}(x^{k}) \right\| _{\mathbf{W}^{-1}}^2\right] . \end{aligned}$$

Thus, if ${\mathbb {E}}\left[ \left\| \mathbf{J}^{k+1} -{\varvec{\nabla }}{} \mathbf{F}(x^{k}) \right\| _{\mathbf{W}^{-1}}^2\right]$ converges to zero, so will ${\mathbb {E}}\left[ \left\| \hat{g}^k - \nabla f(x^k) \right\| _2^2\right] .$ Though unfortunately, the combination of the gradient estimate $\hat{g}^k = \frac{1}{n} \mathbf{J}^{k+1} e$ and a Jacobian estimate updated via (39) will almost always give a biased estimator. For example, if we define ${{\mathcal {D}}}$ by setting ${\mathbf {S}}= e_i$ with probability $\frac{1}{n}$ and let $\mathbf{W}= \mathbf{I}$, then we recover the celebrated SAG method [29] and its biased estimator of the gradient.

The issue with using $\frac{1}{n} \mathbf{J}^{k+1} e$ as an estimator of the gradient is that it decreases the variance too aggressively, neglecting the bias. However, this can be fixed by trading off variance for bias. One way to do this is to introduce the random variable $\theta _{{\mathbf {S}}}$ as a *stochastic relaxation parameter*

42 $$\begin{aligned} \hat{g}^{k} = \frac{1-\theta _{{\mathbf {S}}_k}}{n}\mathbf{J}^{k}e+\frac{\theta _{{\mathbf {S}}_k}}{n} \mathbf{J}^{k+1} e. \end{aligned}$$

If $\theta _{{\mathbf {S}}}$ is bias correcting, we recover the unbiased SAGA estimator (13). By allowing $\theta _{{\mathbf {S}}}$ to be closer to one, however, we will get more bias and lower variance. We leave this strategy of building biased estimators for future work. It is conceivable that SAG could be analyzed using reasonably small modifications of the tools developed in this paper. Doing this would be important due to at least four reasons: (i) SAG was the first variance-reduced method for problem (1), (ii) the existing analysis of SAG is not satisfying, (iii) one may be able to obtain a better rate, (iv) one may be able to develop and analyze novel variants of SAG.

## Convergence analysis for general sketches

In this section we establish a convergence theorem (Theorem 1) which applies to general sketching matrices ${\mathbf {S}}$ (that is, arbitrary distributions ${{\mathcal {D}}}$ from which they are sampled). By design, we keep the setting in this section general, and only deal with specific instantiations and special cases in Sect. 4.

### Two expected smoothness constants

We first formulate two *expected smoothness* assumptions tying together *f*, its Jacobian ${\varvec{\nabla }}{} \mathbf{F}(x)$ and the distribution ${{\mathcal {D}}}$ from which we pick sketch matrices ${\mathbf {S}}$. These assumptions, and the associated expected smoothness constants, play a key role in the convergence result.

Our first assumption concerns the expected smoothness of the stochastic gradients $\nabla f_{{\mathbf {S}}}$ of the stochastic reformulation (22).^10^

#### Assumption 3.1

(*Expected smoothness of the stochastic gradient*) There is a constant ${{\mathcal {L}}}_1>0$ such that

43 $$\begin{aligned} {\mathbb {E}}_{{{\mathcal {D}}}}\left[ \left\| \nabla f_{{\mathbf {S}}}(x) - \nabla f_{{\mathbf {S}}}(x^*) \right\| _2^2 \right] \le 2 {{\mathcal {L}}}_1 (f(x)-f(x^*)), \quad \forall x\in {\mathbb {R}}^d. \end{aligned}$$

It is easy to see from (23) and (32) that

44 $$\begin{aligned} \left\| \nabla f_{{\mathbf {S}}}(x) - \nabla f_{{\mathbf {S}}}(y) \right\| _2^2= & {} \tfrac{1}{n^2}\Vert ({\varvec{\nabla }}{} \mathbf{F}(x) - {\varvec{\nabla }}{} \mathbf{F}(y))\theta _{{\mathbf {S}}} {\varvec{\Pi }}_{{\mathbf {S}}} e\Vert _2^2 \nonumber \\= & {} \left\| \nabla f_{{\mathbf {S}}, \mathbf{J}}(x) - \nabla f_{{\mathbf {S}},\mathbf{J}}(y) \right\| _2^2 \end{aligned}$$

for all $\mathbf{J}\in {\mathbb {R}}^{d\times n}$ and $x,y\in {\mathbb {R}}^d$, and hence the expected smoothness assumption can equivalently be understood from the point of view of the controlled stochastic reformulation. The above assumption is not particularly restrictive. Indeed, in Theorem 2 we provide formulae for ${{\mathcal {L}}}_1$ for smooth functions *f* and for a class of minibatch samplings ${\mathbf {S}}=\mathbf{I}_S$. These formulae can be seen as proofs that Assumption 3.1 is satisfied for a large class of practically relevant sketches ${\mathbf {S}}$ and functions *f*.

Our second expected smoothness assumption concerns the Jacobian of *F*.

#### Assumption 3.2

(*Expected smoothness of the Jacobian*) There is a constant ${{\mathcal {L}}}_2>0$ such that

45 $$\begin{aligned} {\mathbb {E}}_{{{\mathcal {D}}}}\left[ \left\| ({\varvec{\nabla }}{} \mathbf{F}(x)-{\varvec{\nabla }}{} \mathbf{F}(x^*)) {\varvec{\Pi }}_{{\mathbf {S}}} \right\| _{\mathbf{W}^{-1}}^2 \right] \le 2{{\mathcal {L}}}_2 (f(x) -f(x^*)), \quad \forall x\in {\mathbb {R}}^d, \end{aligned}$$

where the norm is the weighted Frobenius norm defined in (10).

It is easy to see (see Lemma 4, Eq. (60)) that for any matrix $\mathbf{M}\in {\mathbb {R}}^{d\times n}$, we have ${\mathbb {E}}_{{{\mathcal {D}}}}\left[ \Vert \mathbf{M}{\varvec{\Pi }}_{{\mathbf {S}}}\Vert _{\mathbf{W}^{-1}}^2\right] = \Vert \mathbf{M}\Vert _{{\mathbb {E}}_{{{\mathcal {D}}}}\left[ \mathbf{H}_{{\mathbf {S}}}\right] }^2,$ where

46 $$\begin{aligned} \mathbf{H}_{\mathbf {S}}\overset{\text {def}}{=}{\mathbf {S}}({\mathbf {S}}^\top \mathbf{W}{\mathbf {S}})^\dagger {\mathbf {S}}^\top \overset{(12)}{=} {\varvec{\Pi }}_{{\mathbf {S}}} \mathbf{W}^{-1}. \end{aligned}$$

Therefore, (45) can be equivalently written in the form

47 $$\begin{aligned} \left\| {\varvec{\nabla }}{} \mathbf{F}(x)-{\varvec{\nabla }}{} \mathbf{F}(x^*) \right\| _{{\mathbb {E}}_{{{\mathcal {D}}}}\left[ \mathbf{H}_{{\mathbf {S}}}\right] }^2 \le 2{{\mathcal {L}}}_2 (f(x) -f(x^*)), \quad \forall x\in {\mathbb {R}}^d, \end{aligned}$$

which suggests that the above condition indeed measures the variation/smoothness of the Jacobian under a specific weighted Frobenius norm.

### Stochastic contraction number

By the *stochastic contraction number* associated with $\mathbf{W}$ and ${{\mathcal {D}}}$ we mean the constant defined by

48 $$\begin{aligned} \kappa = \kappa ({{\mathcal {D}}},\mathbf{W}) \overset{\text {def}}{=}\lambda _{\min } ({\mathbb {E}}_{{{\mathcal {D}}}}\left[ {\varvec{\Pi }}_{\mathbf {S}}\right] ). \end{aligned}$$

In the next lemma we show that $0 \le \kappa \le 1$ for all distributions ${{\mathcal {D}}}$ for which the expectation (48) exists.

#### Lemma 2

For all distributions $\mathcal {D},$ we have the bounds $0 \le \kappa \le 1.$

#### Proof

It is not difficult to show that $\mathbf{W}^{1/2} \mathbf{H}_{\mathbf {S}}\mathbf{W}^{1/2} \overset{(46)}{=}\mathbf{W}^{1/2}{\varvec{\Pi }}_{{\mathbf {S}}} \mathbf{W}^{-1/2}$ is the orthogonal projection matrix that projects onto $\text{ Range }\left( \mathbf{W}^{1/2} {\mathbf {S}}\right)$. Consequently, $0 \preceq \mathbf{W}^{1/2} \mathbf{H}_{\mathbf {S}}\mathbf{W}^{1/2} \preceq \mathbf{I}$ and, after taking expectation, we get $0 \preceq \mathbf{W}^{1/2}{\mathbb {E}}_{{{\mathcal {D}}}}\left[ \mathbf{H}_{\mathbf {S}}\right] \mathbf{W}^{1/2} \preceq \mathbf{I}.$ Finally, this implies that

49 $$\begin{aligned} 0\le \lambda _{\max }(\mathbf{I}- \mathbf{W}^{1/2}{\mathbb {E}}_{{{\mathcal {D}}}}\left[ \mathbf{H}_{\mathbf {S}}\right] \mathbf{W}^{1/2}) = 1 - \lambda _{\min }( \mathbf{W}^{1/2}{\mathbb {E}}_{{{\mathcal {D}}}}\left[ \mathbf{H}_{\mathbf {S}}\right] \mathbf{W}^{1/2}) \le 1. \end{aligned}$$

$$\square $$

In our convergence theorem we will assume that $\kappa >0$. This can be achieved by choosing a suitable distribution ${{\mathcal {D}}}$ and it holds trivially for all the examples we develop. The condition $\kappa >0$ essentially says that the distribution is sufficiently rich. This contraction number was first proposed in [11] in the context of randomized algorithms for solving linear systems. We refer the reader to that work for details on sufficient assumptions about ${{\mathcal {D}}}$ guaranteeing $\kappa >0$. Below we give an example.

#### Example 6

Let $\mathbf{W}\succ 0$, and let ${{\mathcal {D}}}$ be given by setting ${\mathbf {S}}= e_i$ with probability $p_i>0$. Then

$$\begin{aligned} \kappa \,\overset{(48)}{=}\, \lambda _{\min }\left( \mathbf{W}^{1/2}{\mathbb {E}}_{{{\mathcal {D}}}}\left[ {\varvec{\Pi }}_{{\mathbf {S}}}\right] \mathbf{W}^{-1/2}\right) = \lambda _{\min }\left( \sum _{i=1}^n \frac{p_i}{e_i^\top \mathbf{W}e_i} \mathbf{W}^{1/2} e_i e_i^\top \mathbf{W}^{1/2} \right) . \end{aligned}$$

Since the vectors $\mathbf{W}^{1/2}e_i$ span ${\mathbb {R}}^n$ and $p_i>0$ for all *i*, the matrix is positive definite and hence $\kappa >0$. In particular, when $\mathbf{W}=\mathbf{I}$, then the expected projection matrix is equal to $\mathrm{Diag}(p_1,\ldots ,p_n)$ and $\kappa = \min _i p_i>0$. If instead of unit basis vectors $\{e_i\}$ we use vectors that span ${\mathbb {R}}^n$, using similar arguments we can also conclude that $\kappa >0$.

### Convergence theorem

Our main convergence result, which we shall present shortly, holds for $\mu$-strongly convex functions. However, it turns out our results hold for the somewhat larger family of functions that are quasi-strongly convex.

#### Assumption 3.3

(*Quasi-strong convexity*) Function *f* for some $\mu >0$ satisfies

50 $$\begin{aligned} f(x^*) \ge f(x) + \left\langle \nabla f(x), x^*-x\right\rangle + \frac{\mu }{2} \left\| x^*-x \right\| _{2}^2, \quad \forall x \in {\mathbb {R}}^d, \end{aligned}$$

where $$x^* = \arg \min _{x\in {\mathbb {R}}^d} f(x).$$

We are now ready to present the main result of this section.

#### Theorem 1

(Convergence of JacSketch for General Sketches) Let $\mathbf{W}\succ 0$. Let *f* satisfy Assumption 3.3. Let Assumption 2.1 be satisfied (i.e., ${\mathbf {S}}$ is an unbiased sketch and $\theta _{{\mathbf {S}}}$ is the associated bias-correcting random variable). Let the expected smoothness assumptions be satisfied: Assumptions 3.1 and 3.2. Assume that $\kappa >0$. Let the sketch residual be defined as in (37), i.e.,

51 $$\begin{aligned} \rho = \rho (\theta _{{\mathbf {S}}},{{\mathcal {D}}}, \mathbf{W}) \overset{(37)}{=} \lambda _{\max }\left( \mathbf{W}^{1/2}\left( {\mathbb {E}}_{{{\mathcal {D}}}}\left[ \theta _{{\mathbf {S}}}^2 {\varvec{\Pi }}_{{\mathbf {S}}} ee^\top {\varvec{\Pi }}_{{\mathbf {S}}}\right] -ee^\top \right) \mathbf{W}^{1/2}\right) \ge 0.\quad \end{aligned}$$

Choose any $x^0\in {\mathbb {R}}^d$ and $\mathbf{J}^0\in {\mathbb {R}}^{d\times n}$. Let $\{x^k,\mathbf{J}^k\}_{k\ge 0}$ be the random iterates produced by JacSketch (Algorithm 1). Consider the Lyapunov function

52 $$\begin{aligned} \varPsi ^k \overset{\text {def}}{=}\left\| x^k-x^* \right\| _2^2 + \frac{\alpha }{2 {{\mathcal {L}}}_2} \left\| \mathbf{J}^{k} -{\varvec{\nabla }}{} \mathbf{F}(x^*) \right\| _{\mathbf{W}^{-1}}^2. \end{aligned}$$

If the stepsize satisfies

53 $$\begin{aligned} 0 \le \alpha \le \min \left\{ \frac{1}{4 {{\mathcal {L}}}_1 }, \, \frac{\kappa }{4 {{\mathcal {L}}}_2 \rho /n^2 +\mu }\right\} , \end{aligned}$$

then

54 $$\begin{aligned} {\mathbb {E}}\left[ \varPsi ^{k}\right] \le (1-\mu \alpha )^k \cdot \varPsi ^0, \end{aligned}$$

If we choose $\alpha$ to be equal to the upper bound in (53), then

55 $$\begin{aligned} k\ge \max \left\{ \frac{4{{\mathcal {L}}}_1}{\mu }, \; \frac{1}{\kappa } + \frac{4\rho {{\mathcal {L}}}_2}{\kappa \mu n^2} \right\} \log \left( \frac{1}{\epsilon } \right) \Rightarrow {\mathbb {E}}\left[ \varPsi ^{k}\right] \le \epsilon \varPsi ^0. \end{aligned}$$

Recall that the iteration complexity expression from (55) is listed in row 1 of Table 1.

The Lyapunov function we use is simply the sum of the squared distance between $x^k$ to the optimal $x^*$ and the distance of our Jacobian estimate $\mathbf{J}^k$ to the optimal Jacobian ${\varvec{\nabla }}{} \mathbf{F}(x^*).$ Hence, the theorem says that both the iterates $\{x^k\}$ and the Jacobian estimates $\{\mathbf{J}^k\}$ converge.

### Projection lemmas and the stochastic contraction number $\kappa$

In this section we collect some basic results on projections. Recall from (12) that ${\varvec{\Pi }}_{\mathbf {S}}= {\mathbf {S}}({\mathbf {S}}^\top \mathbf{W}{\mathbf {S}})^{\dagger } {\mathbf {S}}^\top \mathbf{W}$ and from (46) that $\mathbf{H}_{\mathbf {S}}= {\mathbf {S}}({\mathbf {S}}^\top \mathbf{W}{\mathbf {S}})^{\dagger } {\mathbf {S}}^\top$.

#### Lemma 3

56 $$\begin{aligned} {\varvec{\Pi }}_{\mathbf {S}}\mathbf{W}^{-1}(\mathbf{I}-{\varvec{\Pi }}_{\mathbf {S}})^\top = 0. \end{aligned}$$

Furthermore,

57 $$\begin{aligned}&{\mathbb {E}}_{{{\mathcal {D}}}}\left[ {\varvec{\Pi }}_{\mathbf {S}}\mathbf{W}^{-1}{\varvec{\Pi }}_{\mathbf {S}}^\top \right] = {\mathbb {E}}_{{{\mathcal {D}}}}\left[ \mathbf{H}_{\mathbf {S}}\right] \quad \text {and}\quad \nonumber \\&\quad {\mathbb {E}}_{{{\mathcal {D}}}}\left[ (\mathbf{I}-{\varvec{\Pi }}_{\mathbf {S}}) \mathbf{W}^{-1} (\mathbf{I}-{\varvec{\Pi }}_{\mathbf {S}})^\top \right] = \mathbf{W}^{-1} - {\mathbb {E}}_{{{\mathcal {D}}}}\left[ \mathbf{H}_{\mathbf {S}}\right] . \end{aligned}$$

#### Proof

Using the pseudoinverse property $\mathbf{A}^\dagger \mathbf{A}\mathbf{A}^\dagger = \mathbf{A}^\dagger$ we have that

58 $$\begin{aligned} {\varvec{\Pi }}_{\mathbf {S}}\mathbf{W}^{-1}{\varvec{\Pi }}_{\mathbf {S}}^\top \overset{(12)}{=} {\mathbf {S}}({\mathbf {S}}^\top \mathbf{W}{\mathbf {S}})^{\dagger } {\mathbf {S}}^\top \mathbf{W}{\mathbf {S}}({\mathbf {S}}^\top \mathbf{W}{\mathbf {S}})^{\dagger } {\mathbf {S}}^\top \overset{(46)}{=} {\varvec{\Pi }}_{\mathbf {S}}\mathbf{W}^{-1} = \mathbf{H}_{\mathbf {S}}, \end{aligned}$$

and as a consequence (56) holds. Moreover,

59 $$\begin{aligned} (\mathbf{I}-{\varvec{\Pi }}_{\mathbf {S}}) \mathbf{W}^{-1} (\mathbf{I}-{\varvec{\Pi }}_{\mathbf {S}})^\top \overset{(56)}{=} \mathbf{W}^{-1} (\mathbf{I}-{\varvec{\Pi }}_{\mathbf {S}})^\top \overset{(46)}{=} \mathbf{W}^{-1} - \mathbf{H}_{\mathbf {S}}. \end{aligned}$$

Finally, taking expectation over (58) and (59) gives (57). $\square$

#### Lemma 4

For any matrices $\mathbf{M}, \mathbf{N}\in {\mathbb {R}}^{d\times n}$ we have the identities

$$\begin{aligned} \left\| \mathbf{M}( \mathbf{I}-{\varvec{\Pi }}_{\mathbf {S}}) + \mathbf{N}{\varvec{\Pi }}_{\mathbf {S}} \right\| _{\mathbf{W}^{-1}}^2 = \left\| \mathbf{M}(\mathbf{I}-{\varvec{\Pi }}_{\mathbf {S}}) \right\| _{\mathbf{W}^{-1}}^2 + \left\| \mathbf{N}{\varvec{\Pi }}_{\mathbf {S}} \right\| _{\mathbf{W}^{-1}}^2 \end{aligned}$$

and

60 $$\begin{aligned} {\mathbb {E}}_{{{\mathcal {D}}}}\left[ \left\| \mathbf{N}{\varvec{\Pi }}_{\mathbf {S}} \right\| _{\mathbf{W}^{-1}}^2\right] = \left\| \mathbf{N} \right\| _{{\mathbb {E}}_{{{\mathcal {D}}}}\left[ \mathbf{H}_{\mathbf {S}}\right] }^2. \end{aligned}$$

Furthermore,

61 $$\begin{aligned} {\mathbb {E}}_{{{\mathcal {D}}}}\left[ \left\| \mathbf{M}( \mathbf{I}-{\varvec{\Pi }}_{\mathbf {S}}) + \mathbf{N}{\varvec{\Pi }}_{\mathbf {S}} \right\| _{\mathbf{W}^{-1}}^2\right] \le (1-\kappa ) \Vert \mathbf{M}\Vert _{\mathbf{W}^{-1}}^2 + \Vert \mathbf{N}\Vert _{{\mathbb {E}}_{{{\mathcal {D}}}}\left[ \mathbf{H}_{\mathbf {S}}\right] }^2. \end{aligned}$$

#### Proof

First, note thatBy taking expectations in ${{\mathcal {D}}}$, we getwhere in the last step we used the estimate

$$\begin{aligned} \mathbf{W}^{-1} - {\mathbb {E}}_{{{\mathcal {D}}}}\left[ \mathbf{H}_{\mathbf {S}}\right]= & {} \mathbf{W}^{-1/2} (\mathbf{I}- \mathbf{W}^{1/2}{\mathbb {E}}_{{{\mathcal {D}}}}\left[ \mathbf{H}_{\mathbf {S}}\right] \mathbf{W}^{1/2}) \mathbf{W}^{-1/2}\\\preceq & {} \lambda _{\max } (\mathbf{I}- \mathbf{W}^{1/2}{\mathbb {E}}_{{{\mathcal {D}}}}\left[ \mathbf{H}_{\mathbf {S}}\right] \mathbf{W}^{1/2}) \mathbf{W}^{-1} \quad \overset{(49)}{=} \quad (1-\kappa ) \, \mathbf{W}^{-1}. \end{aligned}$$

$$\square $$

### Key lemmas

We first establish two lemmas. The first lemma provides an upper bound on the quality of new Jacobian estimate in terms of the quality of the current estimate and function suboptimality. If the second term on the right hand side was not there, the lemma would be postulating a contraction on the quality of the Jacobian estimate.

#### Lemma 5

Let Assumption 3.2 be satisfied. Then iterates of Algorithm 1 satisfy

62 $$\begin{aligned} {\mathbb {E}}_{{{\mathcal {D}}}}\left[ \left\| \mathbf{J}^{k+1} -{\varvec{\nabla }}{} \mathbf{F}(x^*) \right\| _{\mathbf{W}^{-1}}^2\right]\le & {} (1-\kappa ) \left\| \mathbf{J}^{k}-{\varvec{\nabla }}{} \mathbf{F}(x^*) \right\| _{\mathbf{W}^{-1}}^2 \nonumber \\&+\,2{{\mathcal {L}}}_2(f(x^k) -f(x^*)), \end{aligned}$$

where $\kappa$ is defined in (48).

#### Proof

Subtracting ${\varvec{\nabla }}{} \mathbf{F}(x^*)$ from both sides of (39) gives

63 $$\begin{aligned} \mathbf{J}^{k+1} -{\varvec{\nabla }}{} \mathbf{F}(x^*) \, \overset{(39)}{=} \, \underbrace{(\mathbf{J}^{k}-{\varvec{\nabla }}{} \mathbf{F}(x^*))}_{\mathbf{M}}(\mathbf{I}- {\varvec{\Pi }}_{{\mathbf {S}}_k}) + \underbrace{ ({\varvec{\nabla }}{} \mathbf{F}(x^k) -{\varvec{\nabla }}{} \mathbf{F}(x^*))}_{\mathbf{N}}{\varvec{\Pi }}_{{\mathbf {S}}_k}. \end{aligned}$$

Taking norms on both sides, then expectation with respect to ${\mathbf {S}}_k$ and then using Lemma 4, we get$\square$

We now bound the second moment of $g^k$. The lemma implies that as $x^k$ approaches $x^*$ and $\mathbf{J}^k$ approaches ${\varvec{\nabla }}{} \mathbf{F}(x^*)$, the variance of $g^k$ approaches zero. This is a key property of JacSketch which elevates it into the ranks of variance-reduced methods.

#### Lemma 6

Let ${\mathbf {S}}$ be an unbiased sketch. Let Assumption 3.1 be satisfied (i.e., assume that inequality (43) holds for some ${{\mathcal {L}}}_1>0$). Then the second moment of the estimated gradient is bounded by

64 $$\begin{aligned} {\mathbb {E}}_{{{\mathcal {D}}}}\left[ \left\| g^k \right\| _2^2 \right] \le 4 {{\mathcal {L}}}_1 (f(x^k) -f(x^*)) + 2 \frac{\rho }{n^2} \left\| \mathbf{J}^{k} -{\varvec{\nabla }}{} \mathbf{F}(x^*) \right\| _{\mathbf{W}^{-1}}^2, \end{aligned}$$

where $\rho$ is defined in (51).

#### Proof

Adding and subtracting $\tfrac{\theta _{{\mathbf {S}}_k}}{n} {\varvec{\nabla }}{} \mathbf{F}(x^*) {\varvec{\Pi }}_{{\mathbf {S}}_k} e$ in  (13) gives

$$\begin{aligned} g^k = \underbrace{\frac{1}{n}\mathbf{J}^{k}e- \frac{\theta _{{\mathbf {S}}_k}}{n} (\mathbf{J}^{k} -{\varvec{\nabla }}{} \mathbf{F}(x^*)) {\varvec{\Pi }}_{{\mathbf {S}}_k} e}_{b} + \underbrace{\frac{\theta _{{\mathbf {S}}_k}}{n} ({\varvec{\nabla }}{} \mathbf{F}(x^k)-{\varvec{\nabla }}{} \mathbf{F}(x^*)){\varvec{\Pi }}_{{\mathbf {S}}_k} e}_{a}.\qquad \end{aligned}$$

Taking norms on both sides and using the bound $\Vert a+b\Vert _2^2\le 2\Vert a\Vert _2^2 + 2\Vert b\Vert _2^2$ gives

65 $$\begin{aligned} \left\| g^{k} \right\| _2^2\le & {} \underbrace{\frac{2}{n^2} \left\| ({\varvec{\nabla }}{} \mathbf{F}(x^k)-{\varvec{\nabla }}{} \mathbf{F}(x^*)){\varvec{\Pi }}_{{\mathbf {S}}_k} \theta _{{\mathbf {S}}_k} e \right\| _2^2}_{a^k} \nonumber \\&+ \underbrace{\frac{2}{n^2} \left\| \theta _{{\mathbf {S}}_k} (\mathbf{J}^{k} -{\varvec{\nabla }}{} \mathbf{F}(x^*)) {\varvec{\Pi }}_{{\mathbf {S}}_k} e-\mathbf{J}^{k} e \right\| _2^2}_{b^k}. \end{aligned}$$

In view of Assumption 3.1 (combine (43) and (44)), we have

66 $$\begin{aligned} {\mathbb {E}}_{{{\mathcal {D}}}}\left[ a^k\right] \le 4 {{\mathcal {L}}}_1 (f(x^k)-f(x^*)), \end{aligned}$$

where the expectation is taken with respect to ${\mathbf {S}}_k$. Let us now bound ${\mathbb {E}}_{{{\mathcal {D}}}}\left[ b^k\right]$. Using the fact that ${\varvec{\nabla }}{} \mathbf{F}(x^*)e= 0$, we can write

$$\begin{aligned} {\mathbb {E}}_{{{\mathcal {D}}}}\left[ b^k\right]= & {} \frac{2}{n^2} {\mathbb {E}}_{{{\mathcal {D}}}}\left[ \left\| (\mathbf{J}^{k} -{\varvec{\nabla }}{} \mathbf{F}(x^*)) \theta _{{\mathbf {S}}_k} {\varvec{\Pi }}_{{\mathbf {S}}_k} e-(\mathbf{J}^{k}-{\varvec{\nabla }}{} \mathbf{F}(x^*)) e \right\| _2^2\right] \\= & {} \frac{2}{n^2} {\mathbb {E}}_{{{\mathcal {D}}}}\left[ \left\| (\mathbf{J}^{k} -{\varvec{\nabla }}{} \mathbf{F}(x^*)) (\theta _{{\mathbf {S}}_k} {\varvec{\Pi }}_{{\mathbf {S}}_k} - \mathbf{I})e \right\| _2^2\right] \\= & {} \frac{2}{n^2}{\mathbb {E}}_{{{\mathcal {D}}}}\left[ e^\top (\theta _{{\mathbf {S}}_k} {\varvec{\Pi }}_{{\mathbf {S}}_k} - \mathbf{I})^\top (\mathbf{J}^{k} -{\varvec{\nabla }}{} \mathbf{F}(x^*))^\top (\mathbf{J}^{k} -{\varvec{\nabla }}{} \mathbf{F}(x^*)) (\theta _{{\mathbf {S}}_k} {\varvec{\Pi }}_{{\mathbf {S}}_k} - \mathbf{I})e\right] \\= & {} \frac{2}{n^2} {\mathbb {E}}_{{{\mathcal {D}}}}\left[ \text{ Tr }\left( e^\top (\theta _{{\mathbf {S}}_k} {\varvec{\Pi }}_{{\mathbf {S}}_k} - \mathbf{I})^\top (\mathbf{J}^{k} -{\varvec{\nabla }}{} \mathbf{F}(x^*))^\top (\mathbf{J}^{k} -{\varvec{\nabla }}{} \mathbf{F}(x^*)) (\theta _{{\mathbf {S}}_k} {\varvec{\Pi }}_{{\mathbf {S}}_k} - \mathbf{I})e\right) \right] \\= & {} \frac{2}{n^2}{\mathbb {E}}_{{{\mathcal {D}}}}\left[ \text{ Tr }\left( e^\top (\theta _{{\mathbf {S}}_k} {\varvec{\Pi }}_{{\mathbf {S}}_k} - \mathbf{I})^\top \mathbf{W}^{1/2} \mathbf{W}^{-1/2} (\mathbf{J}^{k} -{\varvec{\nabla }}{} \mathbf{F}(x^*))^\top \right. \right. \\&\quad \left. \left. (\mathbf{J}^{k} -{\varvec{\nabla }}{} \mathbf{F}(x^*)) \mathbf{W}^{-1/2} \mathbf{W}^{1/2} (\theta _{{\mathbf {S}}_k} {\varvec{\Pi }}_{{\mathbf {S}}_k} - \mathbf{I})e\right) \right] \\= & {} \frac{2}{n^2} {\mathbb {E}}_{{{\mathcal {D}}}}\left[ \text{ Tr }\left( \mathbf{W}^{-1/2} (\mathbf{J}^{k} -{\varvec{\nabla }}{} \mathbf{F}(x^*))^\top (\mathbf{J}^{k} -{\varvec{\nabla }}{} \mathbf{F}(x^*)) \mathbf{W}^{-1/2} \mathbf{W}^{1/2} (\theta _{{\mathbf {S}}_k} {\varvec{\Pi }}_{{\mathbf {S}}_k} - \mathbf{I})ee^\top \right. \right. \\&\quad \left. \left. (\theta _{{\mathbf {S}}_k} {\varvec{\Pi }}_{{\mathbf {S}}_k} - \mathbf{I})^\top \mathbf{W}^{1/2}\right) \right] \\= & {} \frac{2}{n^2} \text{ Tr }\left( \mathbf{W}^{-1/2} (\mathbf{J}^{k} -{\varvec{\nabla }}{} \mathbf{F}(x^*))^\top (\mathbf{J}^{k} -{\varvec{\nabla }}{} \mathbf{F}(x^*)) \mathbf{W}^{-1/2} {\mathbb {E}}_{{{\mathcal {D}}}}\left[ \mathbf{W}^{1/2} (\theta _{{\mathbf {S}}_k} {\varvec{\Pi }}_{{\mathbf {S}}_k} - \mathbf{I})ee^\top \right. \right. \\&\quad \left. \left. (\theta _{{\mathbf {S}}_k} {\varvec{\Pi }}_{{\mathbf {S}}_k} - \mathbf{I})^\top \mathbf{W}^{1/2}\right] \right) . \end{aligned}$$

If we now let $v=\mathbf{W}^{1/2} (\theta _{{\mathbf {S}}_k} {\varvec{\Pi }}_{{\mathbf {S}}_k} - \mathbf{I})e$ and $\mathbf{M}=(\mathbf{J}^{k} -{\varvec{\nabla }}{} \mathbf{F}(x^*)) \mathbf{W}^{-1/2}$, then we can continue:67where in the last step we have used the assumption that $\theta _{{\mathbf {S}}_k}$ is bias-correcting:

68 $$\begin{aligned} \lambda _{\max }\left( {\mathbb {E}}_{{{\mathcal {D}}}}\left[ vv^\top \right] \right)&\overset{(21)}{=}&\lambda _{\max } \left( \mathbf{W}^{1/2} {\mathbb {E}}_{{{\mathcal {D}}}}\left[ \theta _{{\mathbf {S}}_k}^2{\varvec{\Pi }}_{{\mathbf {S}}_k} ee^\top {\varvec{\Pi }}_{{\mathbf {S}}_k}^\top \right] \mathbf{W}^{1/2} - \mathbf{W}^{1/2} ee^\top \mathbf{W}^{1/2} \right) \nonumber \\&\overset{(51)}{=}&\rho . \end{aligned}$$

It now only remains to substitute (66) and (67) into (65) to arrive at (64). $\square$

### Proof of Theorem 1

With the help of the above lemmas, we now proceed to the proof of the theorem. In view of (50), we have

69 $$\begin{aligned} \left\langle \nabla f(y), y- x^*\right\rangle\ge & {} f(y) -f(x^*) +\frac{\mu }{2} \left\| y - x^* \right\| _2^2. \end{aligned}$$

By using the relationship $x^{k+1} = x^k -\alpha g^k$, the fact that $g^k$ is an unbiased estimate of the gradient $\nabla f(x^k)$, and using one-point strong convexity (69), we get

70 $$\begin{aligned}&{\mathbb {E}}_{{{\mathcal {D}}}}\left[ \left\| x^{k+1} -x^* \right\| _2^2 \right] \,\overset{(2)}{=} \, {\mathbb {E}}_{{{\mathcal {D}}}}\left[ \left\| x^k -x^* - \alpha g^{k} \right\| _2^2\right] \nonumber \\&\quad \overset{(33)}{=} \, \left\| x^k -x^* \right\| _2^2 -2\alpha \left\langle \nabla f(x^k), x^k -x^*\right\rangle + \alpha ^2{\mathbb {E}}_{{{\mathcal {D}}}}\left[ \left\| g^{k} \right\| _2^2\right] \nonumber \\&\quad \overset{(69)}{\le } \,(1-\alpha \mu )\left\| x^k -x^* \right\| _2^2 +\alpha ^2{\mathbb {E}}_{{{\mathcal {D}}}}\left[ \left\| g^{k} \right\| _2^2\right] \nonumber \\&\qquad -2\alpha (f(x^k)-f(x^*)). \end{aligned}$$

Next, applying Lemma 6 leads to the estimate71Let $\sigma = 1 / (2 {{\mathcal {L}}}_2)$. Adding $\sigma \alpha {\mathbb {E}}_{{{\mathcal {D}}}}\left[ \left\| \mathbf{J}^{k+1} -{\varvec{\nabla }}{} \mathbf{F}(x^*) \right\| _{\mathbf{W}^{-1}}^2\right]$ to both sides of the above inequality and substituting in the definition of $\varPsi ^k$ from (52), it follows that

72 $$\begin{aligned}&{\mathbb {E}}_{{{\mathcal {D}}}}\left[ \varPsi ^{k+1}\right] \overset{(71)}{\le } (1-\alpha \mu )\left\| x^k -x^* \right\| _2^2 +2\alpha \left( 2\alpha {{\mathcal {L}}}_1 -1\right) (f(x^k)-f(x^*)) \nonumber \\&\qquad \qquad \qquad \qquad +\, 2\alpha ^2 \frac{\rho }{n^2} \left\| \mathbf{J}^{k} -{\varvec{\nabla }}{} \mathbf{F}(x^*) \right\| _{\mathbf{W}^{-1}}^2 + \sigma \alpha {\mathbb {E}}_{{{\mathcal {D}}}}\left[ \left\| \mathbf{J}^{k+1} -{\varvec{\nabla }}{} \mathbf{F}(x^*) \right\| _{\mathbf{W}^{-1}}^2\right] \nonumber \\&\qquad \overset{(\text {Lemma}~5)}{\le } (1-\alpha \mu )\left\| x^k -x^* \right\| _2^2 +2\alpha \underbrace{\left( {{\mathcal {L}}}_2 \sigma + 2\alpha {{\mathcal {L}}}_1 -1\right) }_{\text {I}} (f(x^k)-f(x^*)) \nonumber \\&\qquad \qquad \qquad +\,\sigma \alpha \underbrace{\left( 1-\kappa +2 \frac{\alpha \rho }{\sigma n^2}\right) }_{\text {II}}\left\| \mathbf{J}^{k} -{\varvec{\nabla }}{} \mathbf{F}(x^*) \right\| _{\mathbf{W}^{-1}}^2. \end{aligned}$$

We now choose $\alpha$ so that $\text {I} \le 0$ and $\text {II} \le 1-\alpha \mu$, which can be written as

73 $$\begin{aligned} \alpha \le \frac{1-{{\mathcal {L}}}_2 \sigma }{2{{\mathcal {L}}}_1} \quad \text {and} \quad \alpha \le \frac{\kappa }{2\rho /(\sigma n^2) + \mu }. \end{aligned}$$

If $\alpha$ satisfies the above two inequalities, then (72) takes on the simplified form ${\mathbb {E}}_{{{\mathcal {D}}}}\left[ \varPsi ^{k+1}\right] \le (1-\alpha \mu ) \varPsi ^k.$ By taking expectation again and using the tower rule, we get ${\mathbb {E}}\left[ \varPsi ^{k}\right] \le (1-\alpha \mu )^k \varPsi ^0$. Note that as long as $k\ge \frac{1}{\alpha \mu } \log \frac{1}{\epsilon }$, we have ${\mathbb {E}}\left[ \varPsi ^k\right] \le \epsilon \varPsi ^0$. Recalling that $\sigma =1/(2 {{\mathcal {L}}}_2)$, and choosing $\alpha$ to be the minimum of the two upper bounds (73) gives the upper bound on (53), which in turn leads to (55). $\square$

## Minibatch sketches

In this section we focus on special cases of Algorithm 1 where one computes $\nabla f_i(x^k)$ for $i\in S^k$, where $S^k$ is a random subset (mini-batch) of [*n*] chosen in each iteration according to some fixed probability law. As we have seen in the introduction, this is achieved by choosing ${\mathbf {S}}_k = \mathbf{I}_{S_k}$.

We say that ${\mathbf {S}}$ is a *minibatch sketch* if ${\mathbf {S}}= \mathbf{I}_{S}$ for some random set (sampling) *S*, where $\mathbf{I}_S \in {\mathbb {R}}^{n\times |S|}$ is a column submatrix of the $n\times n$ identity matrix $\mathbf{I}$ associated with columns indexed by the set *S*. That is, the distribution ${{\mathcal {D}}}$ from which the sketches ${\mathbf {S}}$ are sampled is defined by

$$\begin{aligned} {\mathbb {P}}\left[ {\mathbf {S}}= \mathbf{I}_C\right] = p_{C}, \quad C\subseteq [n], \end{aligned}$$

where $\sum _{C \subseteq [n]} p_C = 1$ and $p_C\ge 0$ for all *C*.

### Samplings

We now formalize the notion of a random set, which we will refer to by the name sampling. A *sampling* is a random set-valued mapping with values being the subsets of [*n*]. A sampling *S* is uniquely characterized by the probabilities $p_C\overset{\text {def}}{=}{\mathbb {P}}\left[ S = C\right]$ associated with every subset *C* of [*n*].

#### Definition 1

(*Types of samplings*) We say that sampling *S* is non-vacuous if ${\mathbb {P}}\left[ S=\emptyset \right] = 0$ (i.e., $p_{\emptyset } = 0$). Let $p_i\overset{\text {def}}{=}{\mathbb {P}}\left[ i \in S\right] = \sum _{C : i\in C} p_C$. We say that *S* is proper if $p_i>0$ for all *i*. We say that *S* is uniform if $p_i=p_j$ for all *i*, *j*. We say that *S* is $\tau$—uniform if it is uniform and $|S|=\tau$ with probability 1. In particular, the unique sampling which assigns equal probabilities to all subsets of [*n*] of cardinality $\tau$ and zero probabilities to all other subsets is called the $\tau$—nice sampling.

We refer the reader to [22, 25] for a background reading on samplings and their properties.

#### Definition 2

(*Support)* The support of a sampling *S* is the set of subsets of [*n*] which are chosen by *S* with positive probability: $\mathrm{supp}(S) \overset{\text {def}}{=}\{C \;:\; p_C > 0\}$. We say that *S* has uniform support if

$$\begin{aligned} c_1 \overset{\text {def}}{=}| \{ C \in \mathrm{supp}(S) \;:\; i\in C \} | = | \{ C \in \mathrm{supp}(S) \;:\; j\in C \} | \end{aligned}$$

for all $i,j\in [n]$. In such a case we say that the support is $c_1$—uniform.

To illustrate the above concepts, we now list a few examples with $n=4$.

#### Example 7

The sampling defined by setting $p_{\{1,2\}} = p_{\{3,4\}} = 0.5$ is non-vacuous, proper, 2—uniform ($p_i=0.5$ for all *i* and $|S|=2$ with probability 1), and has 1—uniform support. If we change the probabilities to $p_{\{1,2\}}=0.4$ and $p_{\{3,4\}}=0.6$, the sampling is no longer uniform (since $p_1=0.4\ne 0.6=p_3$), but it still has 1—uniform support, is proper and non-vacuous. Hence, a sampling with uniform support need not be uniform. On the other hand, a uniform sampling need not have uniform support. As an example, consider sampling *S* defined via $p_{\{1\}} = 0.4$, $p_{\{2,3\}} = p_{\{3,4\}} = p_{\{2,4\}} = 0.2$. It is uniform (since $p_i=0.4$ for all *i*). However, while element 1 appears in a single set of its support, elements 2, 3 and 4 each appear in two sets. So, this sampling does not have uniform support.

#### Example 8

A uniform sampling need not be $\tau$—uniform for any $\tau$. For example, the sampling defined by setting $p_{\{1,2,3,4\}} = 0.5$, $p_{\{1,2\}} = 0.25$ and $p_{\{3,4\}} = 0.25$ is uniform (since $p_i=0.75$ for all *i*), but as it assigns positive probabilities to sets of at least two different cardinalities, it is not $\tau$—uniform for any $\tau$.

#### Example 9

Further, the sampling defined by setting $p_{\{1,2\}} = 1/6$, $p_{\{1,3\}} = 1/6$, $p_{\{1,4\}}=1/6$, $p_{\{2,3\}}=1/6$, $p_{\{2,4\}}=1/6$, $p_{\{3,4\}}=1/6$ is non-vacuous, 2—uniform ($p_i=1/2$ for all *i* and $|S|=2$ with probability 1), and has 3—uniform support. The sampling defined by setting $p_{\{1,2\}} = 1/3$, $p_{\{2,3\}} = 1/3$, $p_{\{3,1\}} = 1/3$ is non-vacuous, proper, 2—uniform ($p_i=2/3$ for all *i* and $|S|=2$ with probability 1) and has 2—uniform support.

Note that a sampling with uniform support is necessarily proper as long as $c_1>0$. However, it need not be non-vacuous. For instance, the sampling *S* defined by setting $p_{\emptyset }=1$ has 0—uniform support and is vacuous. From now on, we only consider samplings with the following properties.

#### Assumption 4.1

*S* is non-vacuous and has $c_1$—uniform support with $c_1\ge 1$.

Note that if *S* is a non-vacuous sampling with 1—uniform support, then its support is necessary a partition of [*n*]. We shall pay specific attention to such samplings in Sect. 5 as for them we can develop a stronger analysis than that provided by Theorem 1.

### Minibatch sketches and projections

In the next result we describe some basic properties of the projection matrix ${\varvec{\Pi }}_{{\mathbf {S}}} = {\mathbf {S}}({\mathbf {S}}^{\top } \mathbf{W}{\mathbf {S}})^\dagger {\mathbf {S}}^\top \mathbf{W}$ associated with a minibatch sketch ${\mathbf {S}}$.

#### Lemma 7

Let $\mathbf{W}= \mathrm{Diag}(w_1,\ldots ,w_n)$. Let *S* be any sampling, ${\mathbf {S}}=\mathbf{I}_S$ be the associated minibatch sketch, and let $\mathbf{P}$ be the probability matrix^11^ associated with sampling *S*: $\mathbf{P}_{ij} = {\mathbb {P}}\left[ i \in S \; \& \; j\in S\right]$. Then

(i) ${\varvec{\Pi }}_{{\mathbf {S}}} = \mathbf{I}_S \mathbf{I}_S^\top$. This is a diagonal matrix with the *i*th diagonal element equal to 1 if $i\in S$, and 0 if $i\notin S$.
(ii) $${\varvec{\Pi }}_{{\mathbf {S}}} e= e_S \overset{\text {def}}{=}\sum _{i\in S} e_i.$$
(iii) $${\mathbb {E}}_{{{\mathcal {D}}}}\left[ {\varvec{\Pi }}_{{\mathbf {S}}} ee^\top {\varvec{\Pi }}_{{\mathbf {S}}}\right] = \sum _{C \subseteq [n]} p_C e_C e_C^\top = \mathbf{P}$$
(iv) $${\mathbb {E}}_{{{\mathcal {D}}}}\left[ {\varvec{\Pi }}_{{\mathbf {S}}}\right] = \mathrm{Diag}(\mathbf{P})$$
(v) The stochastic contraction number defined in (48) is given by $\kappa = \min _{i} p_i$
(vi) Let *S* satisfy Assumption 4.1. Then the random variable
  74 $$\begin{aligned} \theta _{{\mathbf {S}}} \overset{\text {def}}{=}\frac{1}{c_1 p_S}, \end{aligned}$$
  defined on $\mathrm{supp}(S)$, is bias-correcting. That is, ${\mathbb {E}}_{{{\mathcal {D}}}}\left[ {\varvec{\Pi }}_{{\mathbf {S}}} \theta _{{\mathbf {S}}} e\right] = e.$

#### Proof

(i) This follows by noting that $\mathbf{I}_S^\top \mathbf{W}\mathbf{I}_S$ is the $|S|\times |S|$ diagonal matrix with diagonal entries corresponding to $w_i$ for $i\in S$, which in turn can be used to show that $(\mathbf{I}_S^\top \mathbf{W}\mathbf{I}_S)^{-1} \mathbf{I}_S^\top \mathbf{W}= \mathbf{I}_S^\top$.
(ii) This follows from (i) by noting that $\mathbf{I}_S^\top e$ is the vector of all ones in ${\mathbb {R}}^{|S|}$.
(iii) Using (ii), we have ${\varvec{\Pi }}_{{\mathbf {S}}} ee^\top {\varvec{\Pi }}_{{\mathbf {S}}} = e_S e_S^\top$. By linearity of expectation, $\left( {\mathbb {E}}_{{{\mathcal {D}}}}\left[ e_S e_S^\top \right] \right) _{ij} ={\mathbb {E}}_{{{\mathcal {D}}}}\left[ (e_S e_S^\top )_{ij}\right] = {\mathbb {E}}_{{{\mathcal {D}}}}\left[ 1_{i,j \in S}\right] = {\mathbb {P}}\left[ i\in S \; \& \; j\in S\right] = \mathbf{P}_{ij}$, where $1_{i,j\in S} = 1$ if $i,j\in S$ and $1_{i,j\in S} = 0$ otherwise.
(iv) This follows from (i) by taking expectations of the diagonal elements of ${\varvec{\Pi }}_{{\mathbf {S}}}$.
(v) Follows from (iv).
(vi) Indeed,
  75 $$\begin{aligned} {\mathbb {E}}_{{{\mathcal {D}}}}\left[ \theta _{{\mathbf {S}}} {\varvec{\Pi }}_{\mathbf {S}}e\right] \overset{\text {(ii)}}{=} \sum _{C \in \mathrm{supp}(S)} p_C\theta _C e_C \overset{(74)}{=} \frac{1}{c_1}\sum _{C \in \mathrm{supp}(S)} e_C = e, \end{aligned}$$
  where the last equation follows from the assumption that the support of *S* is $c_1$—uniform.$\square$

The following simple observation will be useful in the computation of the constant ${{\mathcal {L}}}_1$. The proof is straightforward and involves a double counting argument.

#### Lemma 8

Let *S* be a sampling satisfying Assumption 4.1. Moreover, assume that *S* is a $\tau$—uniform sampling. Then $\frac{|\mathrm{supp}(S)|}{c_1} = \frac{n}{\tau }$. Consequently, $\kappa = p_1 = p_2 = \cdots = p_n = \frac{\tau }{n} = \frac{c_1}{|\mathrm{supp}(S)|}$, where $\kappa$ is the stochastic contraction number associated with the minibatch sketch ${\mathbf {S}}=\mathbf{I}_S$.

### JacSketch for minibatch sampling = minibatch SAGA

As we have mentioned in Sect. 1.4 already, JacSketch admits a particularly simple form for minibatch sketches, and corresponds to known and new variants of SAGA. Assume that *S* satisfies Assumption 4.1 and let $\mathbf{W}=\mathrm{Diag}(w_1,\ldots ,w_n)$. In view of Lemma 7(vi), this means that the random variable $\theta _{{\mathbf {S}}} = \frac{1}{c_1 p_S }$ is bias-correcting, and due to Lemma 7(ii), we have ${\varvec{\Pi }}_{{\mathbf {S}}_k} e= e_{S_k}= \sum _{i\in S_k} e_i$. Therefore,76By Lemma 7(i), ${\varvec{\Pi }}_{{\mathbf {S}}_k} = \mathbf{I}_{S_k} \mathbf{I}_{S_k}^\top$. In view of (11), the Jacobian estimate gets updated as follows

77 $$\begin{aligned} \mathbf{J}^{k+1}_{:i} = {\left\{ \begin{array}{ll} \mathbf{J}^k_{:i} &{} \quad i\notin S_k,\\ \nabla f_i(x^k)&{} \quad i\in S_k. \end{array}\right. } \end{aligned}$$

The resulting minibatch SAGA method is formalized as Algorithm 2.
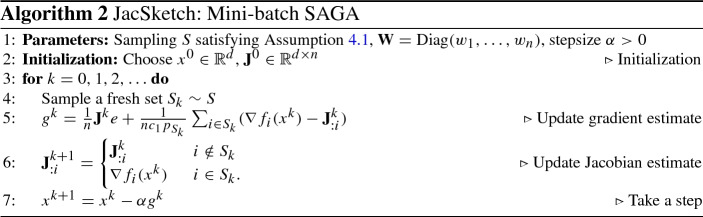


Below we specialize the formula for $g^k$ to a few interesting special cases.

#### Example 10

(Standard SAGA) Standard uniform SAGA is obtained by setting $S_k = \{i\}$ with probability 1/*n* for each $i\in [n]$. Since the support of this sampling is 1—uniform, we set $c_1=1$. This leads to the gradient estimate

78 $$\begin{aligned} g^k = \frac{1}{n} \mathbf{J}^k e+ \nabla f_i(x^k)- \mathbf{J}^k_{:i}. \end{aligned}$$

#### Example 11

(Non-uniform SAGA) However, we can use non-uniform probabilities instead. Let $S_k = \{i\}$ with probability $p_i>0$ for each $i\in [n]$. Since the support of this sampling is 1—uniform, we have $c_1=1$. So, the gradient estimate has the form

79 $$\begin{aligned} g^k = \frac{1}{n} \mathbf{J}^k e+ \frac{1}{np_i} (\nabla f_i(x^k)- \mathbf{J}^k_{:i}). \end{aligned}$$

#### Example 12

(Uniform minibatch SAGA, version 1) Let $C_1,\ldots ,C_q$ be nonempty subsets of forming a partition [*n*]. Let $S_k = C_j$ with probability $p_{C_j}>0$. The support of this sampling is 1—uniform, and hence we can choose $c_1=1$. This leads to the gradient estimate

$$\begin{aligned} g^k = \frac{1}{n} \mathbf{J}^k e+ \frac{1}{np_{C_j}} \sum _{i\in C_j}(\nabla f_i(x^k)- \mathbf{J}^k_{:i}). \end{aligned}$$

#### Example 13

(Uniform minibatch SAGA, version 2) Let $S_k$ be chosen uniformly at random from all subsets of [*n*] of cardinality $\tau \ge 2$. That is, ${\mathbf {S}}_k$ is the $\tau$-nice sampling, and the probabilities are equal to $p_{S_k} = 1/{n \atopwithdelims ()\tau }$. This sampling has $c_1$—uniform support with $c_1 = {n-1 \atopwithdelims ()\tau -1} = \frac{\tau }{n} {n \atopwithdelims ()\tau }$. Thus, $n c_1 p_{S_k}= \tau$, and we have

80 $$\begin{aligned} g^k = \frac{1}{n} \mathbf{J}^k e+ \frac{1}{\tau } \sum _{i\in S_k}(\nabla f_i(x^k)- \mathbf{J}^k_{:i}). \end{aligned}$$

#### Example 14

(Gradient descent) Consider the same situation as in Example 13, but with $\tau =n$. That is, we choose $S_k = [n]$ with probability 1, and $c_1=1$. Then

$$\begin{aligned} g^k = \frac{1}{n} \mathbf{J}^k e+ \frac{1}{n} \sum _{i=1}^n(\nabla f_i(x^k)- \mathbf{J}^k_{:i}) = \nabla f(x^k). \end{aligned}$$

### Expected smoothness constants ${{\mathcal {L}}}_1$ and ${{\mathcal {L}}}_2$

Here we compute the expected smoothness constants ${{\mathcal {L}}}_1$ and ${{\mathcal {L}}}_2$ in the case of ${\mathbf {S}}$ being a minibatch sketch ${\mathbf {S}}=\mathbf{I}_S$, and assuming that *f* is convex and smooth. We first formalize the notion of smoothness we will use.

#### Assumption 4.2

For $$\emptyset \ne C\subseteq [n]$$ define

81 $$\begin{aligned} f_C(x) \overset{\text {def}}{=}\frac{1}{|C|}\sum _{i\in C} f_i(x). \end{aligned}$$

For each $\emptyset \ne C\subseteq [n]$ and all $x\in {\mathbb {R}}^d$, the function $f_C$ is $L_C$—smooth and convex. That is, there exists $L_C\ge 0$ such that the following inequality holds

82 $$\begin{aligned}&\Vert \nabla f_C(x) - \nabla f_C(x^*)\Vert _2^2 \nonumber \\&\quad \le 2L_C \left( f_C(x) - f_C(x^*) - \langle \nabla f_C(x^*), x-x^*\rangle \right) , \quad \forall x\in {\mathbb {R}}^d. \end{aligned}$$

Let $$L_i = L_{\{i\}}$$ for $$i\in [n]$$.

The above assumption is somewhat non-standard. Note that, however, if we instead assume that each $f_i$ is convex and $L_i$-smooth, then the above assumption holds for $L_C = \frac{1}{|C|}\sum _{i\in C} L_i$. In some cases, however, we may have better estimates of the constants $L_C$ than those provided by the averages of the $L_i$ values. The value of these constants will have a direct influence on ${{\mathcal {L}}}_1$ and ${{\mathcal {L}}}_2$, which is why we work with this more refined assumption instead.

#### Lemma 9

(Smoothness of the Jacobian) Assume that $f_i$ is convex and $L_i$—smooth for all $i\in [n]$. Define $L_{\max } \overset{\text {def}}{=}\max _{i} L_i$ and $\mathbf{D}_L \overset{\text {def}}{=}\mathrm{Diag}(L_1, \ldots , L_n) \in {\mathbb {R}}^{n\times n}.$ Then

83 $$\begin{aligned} \left\| {\varvec{\nabla }}{} \mathbf{F}(x) - {\varvec{\nabla }}{} \mathbf{F}(x^*) \right\| _{\mathbf{D}_L^{-1}}^2 \le 2n(f(x) - f(x^*)), \quad \forall x\in {\mathbb {R}}^d. \end{aligned}$$

#### Proof

Indeed,

$$\begin{aligned}&\left\| {\varvec{\nabla }}{} \mathbf{F}(x) - {\varvec{\nabla }}{} \mathbf{F}(x^*) \right\| _{\mathbf{D}_L^{-1}}^2 \\&\overset{(10)}{=} \left\| ({\varvec{\nabla }}{} \mathbf{F}(x) - {\varvec{\nabla }}{} \mathbf{F}(x^*)) \mathbf{D}^{-1/2}_L \right\| ^2 \quad \overset{(10)}{=} \quad \sum _{i=1}^n \frac{1}{L_{i}}\left\| \nabla f_i(x) - \nabla f_i(x^*) \right\| _2^2 \\&\quad \le 2\sum _{i=1}^n (f_i(x) - f_i(x^*) -\left\langle \nabla f_i(x^*), x-x^*\right\rangle ) \quad \overset{(1)}{=} \quad 2n(f(x) - f(x^*)), \end{aligned}$$

where in the last step we used the fact that $\sum _{i=1}^n \nabla f_i(x^*) = n \nabla f(x^*) = 0.$ $\square$

#### Theorem 2

(Expected smoothness) Let ${\mathbf {S}}=\mathbf{I}_S$ be a minibatch sketch where *S* is a sampling satisfying Assumption 4.1 (in particular, the support of *S* is $c_1$—uniform). Consider the bias-correcting random variable $\theta _{\mathbf {S}}$ given in (74). Further, let *f* satisfy Assumption 4.2. Then the expected smoothness assumptions (Assumptions 3.1 and 3.2) are satisfied with constants ${{\mathcal {L}}}_1$ and ${{\mathcal {L}}}_2$ given by^12^

84 $$\begin{aligned} {{\mathcal {L}}}_1 = \frac{ 1}{n c_1^2} \max _i \left\{ \sum _{C \in \mathrm{supp}(S) \;:\; i\in C} \frac{|C| L_C}{p_C}\right\} , \quad {{\mathcal {L}}}_2 = n \ \max _i \left\{ \frac{p_i L_i}{w_i}\right\} , \end{aligned}$$

where $L_i = L_{\{i\}}$. If moreover, *S* is $\tau$—nice sampling, then^13^

85 $$\begin{aligned} {{\mathcal {L}}}_1 = L^{{{\mathcal {G}}}}_{\max }\overset{\text {def}}{=}\max _i \left\{ \frac{1}{c_1} \sum _{C \in \mathrm{supp}(S) \;:\; i\in C} L_C \right\} , \quad {{\mathcal {L}}}_2 = \tau \ \max _i \left\{ \frac{L_i}{w_i}\right\} . \end{aligned}$$

#### Proof

Let $\mathbf{R}= {\varvec{\nabla }}{} \mathbf{F}(x) -{\varvec{\nabla }}{} \mathbf{F}(x^*)$ and $A={\mathbb {E}}_{{{\mathcal {D}}}}\left[ \left\| \nabla f_{{\mathbf {S}}}(x) - \nabla f_{{\mathbf {S}}}(x^*) \right\| _2^2\right]$. Then

$$\begin{aligned}&A \overset{(44)}{=} {\mathbb {E}}_{{{\mathcal {D}}}}\left[ \frac{\theta _{{\mathbf {S}}}^2}{n^2}\left\| \mathbf{R}{\varvec{\Pi }}_{{\mathbf {S}}} e \right\| _2^2\right] \overset{(74)}{=} \sum _{C \in \mathrm{supp}(S)} \frac{p_C}{c_1^2 p_C^2 n^2 } \left\| \mathbf{R}{\varvec{\Pi }}_{\mathbf{I}_C} e \right\| _2^2 \\&\quad = \sum _{C \in \mathrm{supp}(S)} \frac{1}{c_1^2 p_C n^2 } \text{ Tr }\left( e^\top {\varvec{\Pi }}_{\mathbf{I}_C}^\top \mathbf{R}^\top \mathbf{R}{\varvec{\Pi }}_{\mathbf{I}_C} e\right) \\&\quad = \sum _{C \in \mathrm{supp}(S)} \frac{1}{c_1^2 p_C n^2 } \text{ Tr }\left( \mathbf{R}^\top \mathbf{R}{\varvec{\Pi }}_{\mathbf{I}_C} ee^\top {\varvec{\Pi }}_{\mathbf{I}_C}^\top \right) \\&\quad \overset{\text {Lem}~7\text {(iii)}}{=} \sum _{C \in \mathrm{supp}(S)} \frac{1}{c_1^2 p_C n^2 } \text{ Tr }\left( \mathbf{R}^\top \mathbf{R}e_C e_C^\top \right) \\&\quad = \sum _{C \in \mathrm{supp}(S)} \frac{1}{c_1^2 p_C n^2 } \left\| ({\varvec{\nabla }}{} \mathbf{F}(x) -{\varvec{\nabla }}{} \mathbf{F}(x^*))e_C \right\| _2^2 \\&\quad = \sum _{C \in \mathrm{supp}(S)} \frac{|C|^2}{c_1^2 p_C n^2 } \left\| \nabla f_{C}(x)-\nabla f_{C}(x^*) \right\| _2^2. \end{aligned}$$

Using (82) and (81), we can continue:

86 $$\begin{aligned}&A \overset{(82)}{\le } \sum _{C \in \mathrm{supp}(S)} \frac{2L_C |C|^2}{c_1^2 p_C n^2 } (f_{C}(x) - f_{C}(x^*) -\left\langle \nabla f_{C}(x^*), x-x^*\right\rangle )\nonumber \\&\quad \overset{(81)}{=} \frac{2}{c_1^2 n^2}\sum _{C \in \mathrm{supp}(S)} \frac{L_C |C|^2}{ p_C } \frac{1}{|C|}\sum _{i\in C} (f_{i}(x) - f_{i}(x^*) -\left\langle \nabla f_{i}(x^*), x-x^*\right\rangle )\nonumber \\&\quad = \frac{2}{c_1^2 n^2}\sum _{C \in \mathrm{supp}(S)} \sum _{i\in C} (f_{i}(x) - f_{i}(x^*) -\left\langle \nabla f_{i}(x^*), x-x^*\right\rangle )\frac{L_C |C|}{ p_C }\nonumber \\&\quad = \frac{2}{c_1^2 n^2} \sum _{i=1}^n \sum _{C \in \mathrm{supp}(S)\;:\; i\in C} (f_{i}(x) - f_{i}(x^*) -\left\langle \nabla f_{i}(x^*), x-x^*\right\rangle )\frac{L_C |C|}{ p_C }\nonumber \\&\quad = \frac{2}{c_1^2 n^2} \sum _{i=1}^n (f_{i}(x) - f_{i}(x^*) -\left\langle \nabla f_{i}(x^*), x-x^*\right\rangle ) \sum _{C \in \mathrm{supp}(S)\;:\; i\in C} \frac{L_C |C|}{ p_C }\nonumber \\&\quad \le \frac{2}{c_1^2 n} \max _i \left\{ \sum _{C \in \mathrm{supp}(S)\;:\; i\in C} \frac{L_C |C|}{p_C} \right\} \frac{1}{n}\sum _{i=1}^n (f_{i}(x) - f_{i}(x^*) \nonumber \\&\qquad -\left\langle \nabla f_{i}(x^*), x-x^*\right\rangle ), \end{aligned}$$

where in this last inequality we have used convexity of $f_i$ for $i\in [n]$. Since

$$\begin{aligned}&\frac{1}{n}\sum _{i=1}^n \left( f_{i}(x) - f_{i}(x^*) -\left\langle \nabla f_{i}(x^*), x-x^*\right\rangle \right) \\&\quad = f(x) - f(x^*) -\left\langle \nabla f(x^*), x-x^*\right\rangle = f(x) - f(x^*), \end{aligned}$$

the formula for ${{\mathcal {L}}}_1$ now follows by comparing (86) to (43). In order to establish the formula for ${{\mathcal {L}}}_2$, we estimate

87 $$\begin{aligned}&{\mathbb {E}}_{{{\mathcal {D}}}}\left[ \left\| \mathbf{R}{\varvec{\Pi }}_{\mathbf {S}} \right\| _{\mathbf{W}^{-1}}^2\right] \overset{(10)}{=} {\mathbb {E}}_{{{\mathcal {D}}}}\left[ \left\| \mathbf{R}{\varvec{\Pi }}_{\mathbf {S}}\mathbf{W}^{-1/2} \right\| _{\mathbf{I}}^2\right] \overset{(10)}{=} \text{ Tr }\left( \mathbf{R}^\top \mathbf{R}{\mathbb {E}}_{{{\mathcal {D}}}}\left[ {\varvec{\Pi }}_{\mathbf {S}}\mathbf{W}^{-1} {\varvec{\Pi }}_{\mathbf {S}}^\top \right] \right) \nonumber \\&\quad \overset{(57)}{=} \text{ Tr }\left( \mathbf{R}^\top \mathbf{R}{\mathbb {E}}_{{{\mathcal {D}}}}\left[ \mathbf{H}_{\mathbf {S}}\right] \right) \nonumber \\&\quad = \text{ Tr }\left( \mathbf{D}_L^{-1/2} \mathbf{R}^\top \mathbf{R}\mathbf{D}_L^{-1/2} \mathbf{D}_L^{1/2} {\mathbb {E}}_{{{\mathcal {D}}}}\left[ \mathbf{H}_{\mathbf {S}}\right] \mathbf{D}_L^{1/2}\right) \nonumber \\&\quad \le \left\| \mathbf{R} \right\| _{\mathbf{D}_L^{-1}}^2 \lambda _{\max }\left( \mathbf{D}_L^{1/2} {\mathbb {E}}_{{{\mathcal {D}}}}\left[ \mathbf{H}_{\mathbf {S}}\right] \mathbf{D}_L^{1/2}\right) \nonumber \\&\quad \overset{(83)}{\le } 2n \lambda _{\max }\left( \mathbf{D}_L^{1/2} {\mathbb {E}}_{{{\mathcal {D}}}}\left[ \mathbf{H}_{\mathbf {S}}\right] \mathbf{D}_L^{1/2}\right) (f(x^k) -f(x^*)). \end{aligned}$$

From Lemma 7(iv) we have ${\mathbb {E}}_{{{\mathcal {D}}}}\left[ \mathbf{H}_{\mathbf {S}}\right] = {\mathbb {E}}_{{{\mathcal {D}}}}\left[ {\varvec{\Pi }}_{\mathbf {S}}\right] \mathbf{W}^{-1} = \mathbf{P}\mathbf{W}^{-1} = \mathrm{Diag}\left( \frac{p_1}{w_1},\ldots ,\frac{p_n}{w_n}\right)$, and hence $\mathbf{D}_L^{1/2} {\mathbb {E}}_{{{\mathcal {D}}}}\left[ \mathbf{H}_{\mathbf {S}}\right] \mathbf{D}_L^{1/2} = \mathrm{Diag}\left( \frac{p_1 L_1 }{w_1},\ldots , \frac{p_n L_n }{w_n}\right)$. Comparing to the definition of ${{\mathcal {L}}}_2$ in (45) to (87), we conclude that

$$\begin{aligned} {{\mathcal {L}}}_2 = n\lambda _{\max }\left( \mathbf{D}_L^{1/2}\mathbf{P}\mathbf{W}^{-1} \mathbf{D}_L^{1/2}\right) = n \max _{i} \left\{ \frac{p_i L_i}{w_i} \right\} . \end{aligned}$$

The specialized formulas (85) for $\tau$—nice sampling follow as special cases of the general formulas (84) since $$\frac{|C|}{p_C} = \frac{1}{\tau }\left( {\begin{array}{c}n\\ \tau \end{array}}\right) =\frac{n!}{(\tau -1)!(n-\tau )!} = n \left( {\begin{array}{c}n-1\\ \tau -1\end{array}}\right) = n c_1 $$ and $p_i = \tau /n$ for all *i*. $\square$

In the next result we establish some inequalities relating the quantities *L*, $L_{\max }$, $L_C$ and $L^{{{\mathcal {G}}}}_{\max }.$ In particular, the results says that for a certain family of samplings *S* (the same for which we have defined the quantity $L^{{{\mathcal {G}}}}_{\max }$ in (85)), the expected smoothed constant $L^{{{\mathcal {G}}}}_{\max }$ is lower-bounded by the average of $L_C$ over $C\in {{\mathcal {G}}}=\mathrm{supp}(S)$, and upper-bounded by $L_{\max }$.

#### Theorem 3

Let *S* be a $\tau$—uniform sampling ($\tau \ge 1$) with $c_1$—uniform support ($c_1\ge 1$). Let ${{\mathcal {G}}}=\mathrm{supp}(S)$. Then

88 $$\begin{aligned} f(x) = \frac{1}{|{{\mathcal {G}}}|}\sum _{C\in {{\mathcal {G}}}} f_C(x). \end{aligned}$$

Moreover,

89 $$\begin{aligned} L\le \frac{1}{|{{\mathcal {G}}}|} \sum _{C\in {{\mathcal {G}}}} L_C \le L^{{{\mathcal {G}}}}_{\max }\le L_{\max }. \end{aligned}$$

The last inequality holds without the need to assume $\tau$—uniformity.

#### Proof

Using the fact that *S* has $c_1$—uniform support, and utilizing a double-counting argument, we observe that $\sum _{C\in {{\mathcal {G}}}} |C| f_C(x) = c_1 \sum _{i=1}^n f_i(x)$. Multiplying both sides by $\frac{1}{n c_1}$, and since $|C|=\tau$ for all $C\in {{\mathcal {G}}}$, we get $\frac{\tau |{{\mathcal {G}}}|}{c_1 n} \frac{1}{|{{\mathcal {G}}}|}\sum _{C\in {{\mathcal {G}}}} f_C(x) = \frac{1}{n}\sum _{i=1}^n f_i(x) = f(x).$ To obtain (88), it now only remains to use the identity

90 $$\begin{aligned} \frac{\tau |{{\mathcal {G}}}|}{ c_1 n} = 1 \end{aligned}$$

which was shown in Lemma 8. The first inequality in (89) follows from (88) using standard arguments (identical to those that lead to the inequality $L\le \bar{L}$).

Let us now establish the second inequality in (89). Define $L^{{{\mathcal {G}}}}_i\overset{\text {def}}{=}\frac{1}{c_1}\sum _{C\in {{\mathcal {G}}}\;:\; i\in C} L_C$. Again using a double-counting argument we observe that $\tau \sum _{C\in {{\mathcal {G}}}} L_C = c_1 \sum _{i=1}^n L^{{{\mathcal {G}}}}_i.$ Multiplying both sides of this equality by $\frac{|{{\mathcal {G}}}|}{c_1 n}$ and using identity (90), we get $\frac{1}{|{{\mathcal {G}}}|} \sum _{C\in {{\mathcal {G}}}} L_C = \frac{1}{n}\sum _{i=1}^n L^{{{\mathcal {G}}}}_i\le \max _i L^{{{\mathcal {G}}}}_i= L^{{{\mathcal {G}}}}_{\max }.$ We will now establish the last inequality by proving that $L^{{{\mathcal {G}}}}_i\le L_{\max }$ for any *i*:

$$\begin{aligned} L^{{{\mathcal {G}}}}_i= & {} \frac{1}{c_1} \sum _{C\in {{\mathcal {G}}}\;:\; i\in C} L_C \le \frac{1}{c_1} \sum _{C\in {{\mathcal {G}}}\;:\; i\in C} \frac{1}{|C|} \sum _{i\in C} L_i \\\le & {} \frac{1}{c_1} \sum _{C\in {{\mathcal {G}}}\;:\; i\in C} \frac{1}{|C|} \sum _{i\in C} L_{\max } \\= & {} L_{\max } \frac{1}{c_1} \sum _{C\in {{\mathcal {G}}}\;:\; i\in C} \underbrace{\frac{1}{|C|} \sum _{i\in C} 1}_{=1} \le L_{\max } \underbrace{\frac{1}{c_1} \sum _{C\in {{\mathcal {G}}}\;:\; i\in C} 1}_{=1} \le L_{\max }. \end{aligned}$$

Note that we did not need to assume $\tau$—uniformity to prove that $L^{{{\mathcal {G}}}}_{\max }\le L_{\max }$. $\square$

### Estimating the sketch residual $\rho$

In this section we compute the sketch residual $\rho$ for several classes of samplings *S*. Let ${{\mathcal {G}}}=\mathrm{supp}(S)$. We will assume throughout this section that *S* is non-vacuous, has $c_1$—uniform support (with $c_1\ge 1$), and is $\tau$—uniform.

Further, we assume that $\mathbf{W}=\mathrm{Diag}(w_1,\ldots ,w_n)$, and that the bias-correcting random variable $\theta _{\mathbf {S}}$ is chosen as $\theta _{\mathbf {S}}=\tfrac{1}{c_1 p_S} = \tfrac{|{{\mathcal {G}}}|}{c_1}$ (see (75) and Lemma 8). In view of the above, since ${\varvec{\Pi }}_{\mathbf{I}_C}e= e_C$, the sketch residual is given by

91 $$\begin{aligned}&\rho \overset{(51)}{=} \lambda _{\max }\left( \mathbf{W}^{1/2}\left( \frac{|{{\mathcal {G}}}|^2}{c_1^2} {\mathbb {E}}_{{{\mathcal {D}}}}\left[ {\varvec{\Pi }}_{{\mathbf {S}}} ee^\top {\varvec{\Pi }}_{{\mathbf {S}}}\right] -ee^\top \right) \mathbf{W}^{1/2}\right) \nonumber \\&\quad = \lambda _{\max }\left( \mathbf{W}^{1/2}\left( \frac{|{{\mathcal {G}}}|}{c_1^2} \sum _{C \in {{\mathcal {G}}}} e_C e_C^\top -ee^\top \right) \mathbf{W}^{1/2}\right) \nonumber \\&\quad = \lambda _{\max }\left( \left( \frac{|{{\mathcal {G}}}|}{c_1^2} \sum _{C \in {{\mathcal {G}}}} e_C e_C^\top -ee^\top \right) \mathbf{W}\right) , \end{aligned}$$

where the last equality follows by permuting the multiplication of matrices within the $\lambda _{\max }.$

In the following text we calculate upper bounds for $\rho$ for $\tau$—partition and $\tau$—nice samplings. Note that Theorem 1 still holds if we use an upper bound of $\rho$ in place of $\rho$.

#### Theorem 4

If *S* is the $\tau$—partition sampling, then

92 $$\begin{aligned} \rho \le \frac{n}{\tau } \max _{C \in {{\mathcal {G}}}} \sum _{i \in C} w_i. \end{aligned}$$

#### Proof

Using Lemma 8, and since $c_1 =1$, we get $\frac{|{{\mathcal {G}}}|}{c_1^2} = \frac{n}{\tau }$. Consequently,

93 $$\begin{aligned} \rho \overset{(91)}{\le } \frac{n}{\tau }\lambda _{\max }\left( \sum _{C \in {{\mathcal {G}}}} e_C e_C^\top \mathbf{W}\right) = \frac{n}{\tau }\lambda _{\max }\left( \sum _{C \in {{\mathcal {G}}}} e_C w_C^\top \right) , \end{aligned}$$

where $w_C=\sum _{i \in C} w_i e_i$ and we used that $-\mathbf{W}^{1/2} ee^\top \mathbf{W}^{1/2}$ is negative semidefinite. When $\mathbf{W}= \mathbf{I}$, the above bound is tight. By Gershgorin’s theorem, every eigenvalue $\lambda$ of the matrix is bounded by at least one of the inequalities $\lambda \le \sum _{i \in C} w_i$ for $C \in {{\mathcal {G}}}$. Consequently, from (93) we have that $\rho \le \frac{n}{\tau } \max _{C \in {{\mathcal {G}}}} \sum _{i \in C} w_i.$ $\square$

Next we give an useful upper bound on $\rho$ for a large family of uniform samplings (for proof, see “Appendix C”).

#### Theorem 5

Let ${{\mathcal {G}}}$ be a collection of subsets of [*n*] with the property that the number of sets $C\in {{\mathcal {G}}}$ containing distinct elements $i,j\in [n]$ is the same for all *i*, *j*. In particular, define

94 $$\begin{aligned} c_2\overset{\text {def}}{=}|\{C \, : \, \{1,2\} \subseteq C, \, C \in {{\mathcal {G}}}\}|. \end{aligned}$$

Now define a sampling *S* by setting $S=C\in {{\mathcal {G}}}$ with probability $\frac{1}{|{{\mathcal {G}}}|}$. Moreover, assume that the support of *S* is $c_1$—uniform. Consider the minibatch sketch ${\mathbf {S}}=\mathbf{I}_{S}$.

(i) If $$\mathbf{W}=\mathrm{Diag}(w_{1}, \ldots , w_{n})$$, then
  95 $$\begin{aligned} \rho \le \max _{i=1,\ldots , n} \left\{ \left( \frac{|{\mathcal {G}}|}{c_1} -1\right) w_i+\sum _{j\ne i} w_j \left| \frac{| {{\mathcal {G}}}| c_2}{c_1^2}-1\right| \right\} . \end{aligned}$$
(ii) If $\mathbf{W}= \mathbf{I}$, then
  96 $$\begin{aligned} \rho = \max \left\{ \frac{| {{\mathcal {G}}}| }{c_1}\left( 1+ (n-1) \frac{c_2}{c_1}\right) -n ,\frac{| {{\mathcal {G}}}| }{c_1}\left( 1 - \frac{c_2}{c_1}\right) \right\} . \end{aligned}$$

Note that as long as $\tau \ge 2$, the $\tau$—nice sampling *S* satisfies the assumptions of the above theorem. Indeed, ${{\mathcal {G}}}$ is the support of *S* consisting of all subsets of [*n*] of size $\tau$, $|{{\mathcal {G}}}| = {n \atopwithdelims ()\tau }$, $c_1= {n-1 \atopwithdelims ()\tau -1}$, and $c_2={n-2 \atopwithdelims ()\tau -2}$. As a result, bound (95) simplifies to

97 $$\begin{aligned} \rho \le \left( \frac{n}{\tau } -1\right) \max _{i=1,\ldots , n} \left\{ w_i+\frac{1}{n-1}\sum _{j\ne i} w_j \right\} , \end{aligned}$$

and (96) simplifies to

98 $$\begin{aligned} \rho = \frac{n}{\tau }\frac{n-\tau }{n-1}. \end{aligned}$$

### Calculating the iteration complexity for special cases

In this section we consider minibatch SAGA (Algorithm 2) and calculate its iteration complexity in special cases using Theorem 1 by pulling together the formulas for ${{\mathcal {L}}}_1, {{\mathcal {L}}}_2, \kappa$ and $\rho$ established in previous sections. In particular, assume *S* is $\tau$—uniform and has $c_1$—uniform support with $c_1\ge 1$. In this case, formula (85) for ${{\mathcal {L}}}_1,{{\mathcal {L}}}_2$ from Lemma 2 applies and we have ${{\mathcal {L}}}_1 = L^{{{\mathcal {G}}}}_{\max }$ and ${{\mathcal {L}}}_2 = \tau \max _i \left\{ \frac{L_i}{w_i}\right\}$.

Moreover, by Lemma 8, $\kappa =\tfrac{\tau }{n}$. By Theorem 1, if we use the stepsize

99 $$\begin{aligned} \alpha= & {} \min \left\{ \frac{1}{4 {{\mathcal {L}}}_1 }, \, \frac{\kappa }{4 {{\mathcal {L}}}_2 \rho /n^2 +\mu }\right\} \nonumber \\= & {} \frac{1}{4}\min \left\{ \frac{1}{ L^{{{\mathcal {G}}}}_{\max }}, \, \frac{1 }{ \frac{\rho }{n}\max _{j=1,\ldots ,n} \left\{ \frac{L_j}{w_j} \right\} +\frac{\mu }{4} \frac{n}{\tau } }\right\} , \end{aligned}$$

then the iteration complexity is given by

100 $$\begin{aligned}&\max \left\{ \frac{4 {{\mathcal {L}}}_1}{\mu }, \, \frac{1}{\kappa } + \frac{4 \rho {{\mathcal {L}}}_2 }{\kappa \mu n^2} \right\} \log \left( \frac{1}{\epsilon }\right) \nonumber \\&\quad = \max \left\{ \frac{4L^{{{\mathcal {G}}}}_{\max }}{\mu }, \, \frac{n}{\tau } + \frac{4 \rho }{\mu n} \max _{i} \left\{ \frac{L_i}{w_i} \right\} \right\} \log \left( \frac{1}{\epsilon }\right) . \end{aligned}$$

Complexity (100) is listed in line 9 of Table 1. The complexities in lines 3, 5 and 10–13 arise as special cases of (100) for specific choices of *S*:

- In line 3 we have *gradient descent*. This arises for the choice $\mathbf{W}=\mathbf{I}$ and $S=[n]$ with probability 1. In this case, $\tau =n$, $L^{{{\mathcal {G}}}}_{\max }= L$ and $\rho =0$. So, (100) simplifies to $\frac{4L}{\mu } \log \left( \frac{1}{\epsilon }\right)$.
- In line 5 we have *uniform SAGA.* We choose $\mathbf{W}=\mathbf{I}$ and $S=\{i\}$ with probability 1/*n*. We have $\tau =1$ and $L^{{{\mathcal {G}}}}_{\max }= L_{\max }$. In view of Theorem 4, $\rho \le n$. So, (100) simplifies to $\left( n+\frac{4L_{\max }}{\mu }\right) \log \left( \frac{1}{\epsilon }\right)$.
- In line 10 we choose $\mathbf{W}=\mathbf{I}$ and *S* is the $\tau$-nice sampling. In this case, Theorem 5 says that $\rho = \frac{n}{\tau }\frac{n-\tau }{n-1}$ (see (98)). Therefore, (100) reduces to
  101 $$\begin{aligned} \max \left\{ \frac{4L^{{{\mathcal {G}}}}_{\max }}{\mu }, \, \frac{n}{\tau } + \frac{n-\tau }{(n-1)\tau }\frac{4 L_{\max }}{\mu } \right\} \log \left( \frac{1}{\epsilon }\right) . \end{aligned}$$
- In line 11 we choose $\mathbf{W}=\mathrm{Diag}(L_i)$ and *S* is the $\tau$-nice sampling. Theorem 5 says that $\rho \le \tfrac{n-\tau }{\tau }\left( \tfrac{n-2}{n-1}L_{\max } + \tfrac{n}{n-1} \bar{L}\right)$ (see (97)). Therefore, (100) reduces to
  102 $$\begin{aligned} \max \left\{ \frac{4L^{{{\mathcal {G}}}}_{\max }}{\mu }, \, \frac{n}{\tau } + \frac{n-\tau }{\tau n} \frac{4 \left( \tfrac{n-2}{n-1}L_{\max } + \tfrac{n}{n-1}\bar{L}\right) }{\mu } \right\} \log \left( \frac{1}{\epsilon }\right) . \end{aligned}$$
  To simplify the above expression, one may further use the bound $\tfrac{n-2}{n-1}L_{\max } + \tfrac{n}{n-1}\bar{L} \le L_{\max } + \bar{L}$. In Table 1 we have listed the complexity in this simplified form.
- In line 12 of Table 1 we let $\mathbf{W}=\mathbf{I}$ and *S* is the $\tau$-partition sampling. In view of Theorem 4, $\rho \le \tfrac{n}{\tau }\tau = n$ and hence (100) reduces to
  103 $$\begin{aligned} \max \left\{ \frac{4L^{{{\mathcal {G}}}}_{\max }}{\mu }, \, \frac{n}{\tau } + \frac{4 L_{\max }}{\mu } \right\} \log \left( \frac{1}{\epsilon }\right) . \end{aligned}$$
- In line 13 of Table 1 we let $\mathbf{W}=\mathrm{Diag}(L_i)$ and *S* is the $\tau$-partition sampling. In view of Theorem 4, $\rho \le \frac{n}{\tau }\max _{C\in {{\mathcal {G}}}} \sum _{i\in C} L_i$ and hence (100) reduces to
  104 $$\begin{aligned} \max \left\{ \frac{4L^{{{\mathcal {G}}}}_{\max }}{\mu }, \, \frac{n}{\tau } + \frac{4 \max _{C\in {{\mathcal {G}}}} \sum _{i\in C} L_i}{\mu \tau } \right\} \log \left( \frac{1}{\epsilon }\right) . \end{aligned}$$
  Note that the previous bound for $\mathbf{W}=\mathbf{I}$ is better than this bound since $\max _{C \in {{\mathcal {G}}}}\sum _{i \in C} L_i \le \tau L_{\max }.$

### Comparison with previous mini-batch SAGA convergence results

Recently in [14], a method that includes a mini-batch variant of SAGA was proposed. This work is the most closely related to our minibatch SAGA. The methods described in [14] can be cast in our framework. In the language of our paper, in [14] the authors update the Jacobian estimate according to (77), where $S_k$ is sampled according to a uniform probability with $p_i = \tau /n,$ for all $i=1,\ldots , n.$ What [14] do differently is that instead of introducing the bias-corecting random variable $\theta _{{\mathbf {S}}}$ to maintain an unbiased gradient estimate, the gradient estimate is updated using the standard SAGA update (78) and this sampling process is done independently of how $S_k$ is sampled for the Jacobian update. Thus at every iteration a gradient $\nabla f_i(x^k)$ is sampled to compute (78), but is then discarded and not used to update the Jacobian update so as to maintain the independence between $\mathbf{J}^{k}$ and $g^k.$ By introducing the bias-correcting random variable $\theta _{{\mathbf {S}}}$ in our method we avoid the data-hungry strategy used in [14].

The analysis provided in [14] shows that, by choosing the stepsize appropriately, the expectation of a Lyapunov function similar to (52) is less than $\epsilon >0$ after

105 $$\begin{aligned} \frac{1}{2} \left( \frac{n}{\tau } + K +\sqrt{\frac{n^2}{\tau ^2}+K^2}\right) \log \left( \frac{1}{\epsilon }\right) \end{aligned}$$

iterations, where $K \overset{\text {def}}{=}\frac{4 L_{\max }}{ \mu }$. When $\tau =1$ this gives an iteration complexity of $O(n +K)\log \frac{1}{\epsilon },$ which is essentially the same complexity as the standard SAGA method. The main issue with this complexity is that it decreases only very modestly as $\tau$ increases. In particular, on the extreme end when $\tau =n$, since $K \ge 4$, we can approximate $(1 +K)^2 \approx 1+K^2$ and the resulting complexity (105) becomes

$$\begin{aligned} \left( 1+\frac{4L_{\max }}{\mu }\right) \log \left( \frac{1}{\epsilon }\right) . \end{aligned}$$

**Fig. 1:**
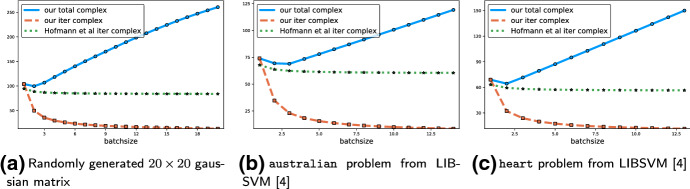
The iteration complexity of minibatch SAGA (80) vs the mini-batch size $\tau$ for two ridge regression problems (132). We used $\lambda = L_{\max }/n$

Yet we know that $\tau =n$ corresponds to gradient descent, and thus the iteration complexity should be $O(\tfrac{ L}{ \mu }\log (1/\epsilon )),$ which is what we recover in the analysis of all our mini-batch variants. In Fig. 1a–c in the experiments in Sect. 6 we illustrate how (105) descreases very modestly as $\tau$ increases.

## A refined analysis with a stochastic Lyapunov function

In this section we perform a refined analysis of JacSketch applied with a minibatch sketch ${\mathbf {S}}=\mathbf{I}_S$ where the sampling *S* is over partitions of [*n*] into sets of size $\tau$.^14^

### Assumption 5.1

Let ${{\mathcal {G}}}$ be a partition of [*n*] into sets of size $\tau$. Assume that the sampling *S* picks sets from the partition ${{\mathcal {G}}}$ uniformly at random. That is, $p_C \overset{\text {def}}{=}{\mathbb {P}}\left[ S=C\right]$ for $C\in {{\mathcal {G}}}= \mathrm{supp}(S)$. A sampling with these properties is called a $\tau$—partition sampling.

In the terminology introduced in Sect. 4.1, a $\tau$—partition sampling is non-vacuous, proper and $\tau$—uniform. Its support is a partition of [*n*], and is 1—uniform. It satisfies Assumption 4.1. Restricting our attention to $\tau$—partition samplings will allow us to perform a more in-depth analysis of JacSketch using a *stochastic Lyapunov function*.

One of the key reasons why we restrict our attention to $\tau$-partition samplings is the fact that

106 $$\begin{aligned} \mathbf{I}_{C_1}^\top \mathbf{I}_{C_2} = {\left\{ \begin{array}{ll} \mathbf{I}\in {\mathbb {R}}^{\tau \times \tau }, &{} \quad C_1 = C_2,\\ 0 \in {\mathbb {R}}^{\tau \times \tau }, &{} \quad C_1 \ne C_2, \end{array}\right. } \end{aligned}$$

for $C_1, C_2\in {{\mathcal {G}}}$. Recall from Lemma 7 that if $\mathbf{W}=\mathbf{I}$, then ${\varvec{\Pi }}_{\mathbf{I}_C} = \mathbf{I}_C \mathbf{I}_C^\top$. Consequently, for $C_1,C_2\in {{\mathcal {G}}}$ we have

107 $$\begin{aligned} C_1\ne C_2 \Rightarrow {\varvec{\Pi }}_{\mathbf{I}_{C_1}} {\varvec{\Pi }}_{\mathbf{I}_{C_2}} =0, \quad C_1 =C_2 \Rightarrow (\mathbf{I}-{\varvec{\Pi }}_{\mathbf{I}_{C_1}}) {\varvec{\Pi }}_{\mathbf{I}_{C_2}} = 0. \end{aligned}$$

This orthogonality property will be fundamental for controlling the convergence of the gradient estimate in Lemma 10.

### Convergence theorem

Recall from (32) that the stochastic gradient of the controlled stochastic reformulation (28) of the original finite-sum problem (1) is given by

108 $$\begin{aligned} \nabla f_{\mathbf{I}_S,\mathbf{J}}(x) = \frac{1}{n} \mathbf{J}e+ \frac{1}{p_S n} ({\varvec{\nabla }}{} \mathbf{F}(x)-\mathbf{J}) {\varvec{\Pi }}_{\mathbf{I}_S}e\end{aligned}$$

provided that we use the minibatch sketch ${\mathbf {S}}= \mathbf{I}_S$ and bias-correcting variable $\theta _{{\mathbf {S}}} = \theta _{\mathbf{I}_S} = 1/p_S$ given by Lemma 7(vi). This object will appear in our Lyapunov function, evaluated at $x=x^*$ and $\mathbf{J}=\mathbf{J}^k$. We are now ready to present the main result of this section.

#### Theorem 6

(Convergence for minibatch sketches with $\tau$-partition samplings) Let

(i) ${\mathbf {S}}$ be a minibatch sketch (i.e., ${\mathbf {S}}=\mathbf{I}_S$),^15^ where *S* is a $\tau$—partition sampling with support ${{\mathcal {G}}}= \mathrm{supp}(S)$.
(ii) $f_C \overset{\text {def}}{=}\tfrac{1}{|C|}\sum _{i\in C} f_i$ be $L_C$—smooth and $\mu$—strongly convex (for $\mu >0$) for all $C \in {{\mathcal {G}}}$.
(iii) $$\mathbf{W}= \mathbf{I}$$, $$\theta _{{\mathbf {S}}} = \frac{1}{p_S}$$.
(iv) $\{x^k, \mathbf{J}^k\}$ be the iterates produced by JacSketch.

Consider the *stochastic Lyapunov function*

109 $$\begin{aligned} \varPsi _S^k \overset{\text {def}}{=}\left\| x^{k} -x^* \right\| _2^2 +2\sigma _{S} \alpha \left\| \frac{1}{n}\mathbf{J}^{k}e- \nabla f_{\mathbf{I}_S,\mathbf{J}^{k}}(x^*) \right\| _2^2, \end{aligned}$$

where $\sigma _S = \frac{n}{4\tau L_{S}}$ is a *stochastic Lyapunov constant*. If we use a stepsize that satisfies

110 $$\begin{aligned} \alpha \le \min _{C \in {{\mathcal {G}}}}\ \frac{p_C }{\mu +\frac{4 L_C\tau }{n} }, \end{aligned}$$

then

111 $$\begin{aligned} {\mathbb {E}}\left[ \varPsi _S^{k} \right] \le (1-\mu \alpha )^k \cdot {\mathbb {E}}\left[ \varPsi _S^0 \right] . \end{aligned}$$

This means that if we choose the stepsize equal to the upper bound (110), then

112 $$\begin{aligned} k\ge \max _{C \in {{\mathcal {G}}}}\left\{ \frac{1}{p_C}+\frac{4 L_C}{\mu }\frac{\tau }{n p_C} \right\} \log \left( \frac{1}{\epsilon } \right) \Rightarrow {\mathbb {E}}\left[ \varPsi _S^k\right] \le \epsilon \cdot {\mathbb {E}}\left[ \varPsi ^0_S\right] . \end{aligned}$$

### Gradient estimate contraction

Here we will show that our gradient estimate contracts in the following sense.

#### Lemma 10

Let *S* be the $\tau$—partition sampling, and $\sigma (S) \overset{\text {def}}{=}\sigma _{S}\ge 0$ be any non-negative random variable. Then

113 $$\begin{aligned} {\mathbb {E}}\left[ \sigma _{S} \left\| \frac{1}{n}\mathbf{J}^{k+1}e- \nabla f_{\mathbf{I}_S, \mathbf{J}^{k+1}}(x^*) \right\| _2^2\right]\le & {} {\mathbb {E}}\left[ \sigma _S ( 1 -p_S) \left\| \frac{1}{n}\mathbf{J}^{k}e- \nabla f_{\mathbf{I}_S, \mathbf{J}^{k}}(x^*) \right\| _2^2\right] \nonumber \\&+ {\mathbb {E}}\left[ \sigma _{S} p_{S} \left\| \nabla f_{\mathbf{I}_S, \mathbf{J}^{k}}(x^{k}) - \nabla f_{\mathbf{I}_S, \mathbf{J}^{k}}(x^*) \right\| _2^2\right] . \nonumber \\ \end{aligned}$$

#### Proof

For simplicity, in this proof we let ${\varvec{\nabla }}{} \mathbf{F}^k = {\varvec{\nabla }}{} \mathbf{F}(x^k)$ and ${\varvec{\nabla }}{} \mathbf{F}^* = {\varvec{\nabla }}{} \mathbf{F}(x^*)$. Rearranging (108), we have

114 $$\begin{aligned}&\frac{1}{n}\mathbf{J}^{k+1}e- \nabla f_{\mathbf{I}_S, \mathbf{J}^{k+1}}(x^*) \overset{(108)}{=} \frac{1}{np_{S}}(\mathbf{J}^{k+1}-{\varvec{\nabla }}{} \mathbf{F}^* ) {\varvec{\Pi }}_{\mathbf{I}_S} e\nonumber \\&\quad \overset{(39)}{=} \frac{1}{np_{S}}\left( \mathbf{J}^{k} -(\mathbf{J}^{k}-{\varvec{\nabla }}{} \mathbf{F}^{k}) {\varvec{\Pi }}_{\mathbf{I}_{S_k}}-{\varvec{\nabla }}{} \mathbf{F}^* \right) {\varvec{\Pi }}_{\mathbf{I}_S} e\nonumber \\&\quad = \frac{1}{np_{S}}(\mathbf{J}^{k}-{\varvec{\nabla }}{} \mathbf{F}^*)(\mathbf{I}- {\varvec{\Pi }}_{\mathbf{I}_{S_k}}){\varvec{\Pi }}_{\mathbf{I}_S}e\nonumber \\&\qquad + \frac{1}{np_{S}}({\varvec{\nabla }}{} \mathbf{F}^{k} -{\varvec{\nabla }}{} \mathbf{F}^*){\varvec{\Pi }}_{\mathbf{I}_{S_k}}{\varvec{\Pi }}_{\mathbf{I}_S}e. \end{aligned}$$

Taking norm squared on both sides gives

115 $$\begin{aligned} \Big \Vert \frac{1}{n}\mathbf{J}^{k+1}e-\nabla f_{\mathbf{I}_S, \mathbf{J}^{k+1}}(x^*)\Big \Vert _2^2= & {} \underbrace{\frac{1}{n^2p_{S}^2}\Big \Vert \overbrace{(\mathbf{J}^{k}-{\varvec{\nabla }}{} \mathbf{F}^*)}^{\mathbf{A}}(\mathbf{I}- {\varvec{\Pi }}_{\mathbf{I}_{S_k}}){\varvec{\Pi }}_{\mathbf{I}_S}e\Big \Vert _2^2}_{\text {I}}\nonumber \\&+\underbrace{\frac{1}{n^2p_{S}^2}\Big \Vert \overbrace{ ({\varvec{\nabla }}{} \mathbf{F}^{k} -{\varvec{\nabla }}{} \mathbf{F}^*)}^{\mathbf{R}}{\varvec{\Pi }}_{\mathbf{I}_{S_k}}{\varvec{\Pi }}_{\mathbf{I}_S}e\Big \Vert _2^2}_{\text {II}}\nonumber \\&+ 2\frac{1}{n^2p_{S}^2}\underbrace{\left\langle (\mathbf{J}^{k}-{\varvec{\nabla }}{} \mathbf{F}^*)(\mathbf{I}- {\varvec{\Pi }}_{\mathbf{I}_{S_k}}){\varvec{\Pi }}_{\mathbf{I}_S}e({\varvec{\nabla }}{} \mathbf{F}^{k} -{\varvec{\nabla }}{} \mathbf{F}^*){\varvec{\Pi }}_{\mathbf{I}_{S_k}}{\varvec{\Pi }}_{\mathbf{I}_S}e\right\rangle }_{\text {III}}.\nonumber \\ \end{aligned}$$

First, it follows from (107) that expression III is zero. We now multiply expressions I and II by $\sigma _{S}$ and bound certain conditional expectations of these terms. Since *S* and $S_k$ are independent samplings, we have

116 $$\begin{aligned}&{\mathbb {E}}\left[ \frac{\sigma _{S}}{n^2 p_{S}^2}\left\| \mathbf{A}(\mathbf{I}- {\varvec{\Pi }}_{\mathbf{I}_{S_k}}){\varvec{\Pi }}_{\mathbf{I}_S}e \right\| _2^2 \;|\; \mathbf{A}\right] = \sum _{C \in {{\mathcal {G}}}}\sum _{C' \in {{\mathcal {G}}}} p_{C} p_{C'} \frac{\sigma _{C}}{n^2 p_{C}^2} \left\| \mathbf{A}(\mathbf{I}- {\varvec{\Pi }}_{\mathbf{I}_{C'}}){\varvec{\Pi }}_{\mathbf{I}_{C}}e \right\| _2^2\nonumber \\&\quad \overset{(107)}{=} \sum _{C \in {{\mathcal {G}}}} \frac{\sigma _{C}}{n^2 p_{C}} \left\| \mathbf{A}{\varvec{\Pi }}_{\mathbf{I}_{C}}e \right\| _2^2 \sum _{C' \in {{\mathcal {G}}}, \, C' \ne C} p_{C'} \nonumber \\&\quad = \sum _{C \in {{\mathcal {G}}}} \frac{\sigma _{C}}{n^2 p_{C}} ( 1 -p_C)\left\| \mathbf{A}{\varvec{\Pi }}_{\mathbf{I}_{C}}e \right\| _2^2\nonumber \\&\quad = \sum _{C \in {{\mathcal {G}}}} p_C \sigma _{C}( 1 -p_C) \frac{1}{n^2 p_{C}^2} \Big \Vert \mathbf{A}{\varvec{\Pi }}_{\mathbf{I}_{C}}e\Big \Vert _2^2\nonumber \\&\quad \overset{(114)}{ =} {\mathbb {E}}\left[ \sigma _S ( 1 -p_S)\Big \Vert \frac{1}{n}\mathbf{J}^{k}e- \nabla f_{\mathbf{I}_S, \mathbf{J}^{k}}(x^*) \Big \Vert _2^2 \;|\; \mathbf{J}^{k}\right] . \end{aligned}$$

Taking conditional expectation over expression II yields

117 $$\begin{aligned}&{\mathbb {E}}\left[ \frac{\sigma _{S}}{n^2p_{S}^2}\Big \Vert \mathbf{R}{\varvec{\Pi }}_{\mathbf{I}_{S_k}}{\varvec{\Pi }}_{\mathbf{I}_S}e\Big \Vert _2^2 \;|\; \mathbf{R}, S_k\right] \nonumber \\&\quad = \sum _{C \in {{\mathcal {G}}}}p_C \frac{\sigma _C}{n^2 p_C^2} \left\| \mathbf{R}{\varvec{\Pi }}_{\mathbf{I}_{S_k}}{\varvec{\Pi }}_{\mathbf{I}_C}e \right\| _2^2 \nonumber \\&\quad \overset{(107)}{=} \frac{\sigma _{S_{k}}}{ n^2 p_{S_{k}}} \left\| \mathbf{R}{\varvec{\Pi }}_{\mathbf{I}_{S_k}}{\varvec{\Pi }}_{\mathbf{I}_{S_k}} e \right\| _2^2 \;= \;\frac{\sigma _{S_{k}}}{ n^2 p_{S_{k}}} \left\| \mathbf{R}{\varvec{\Pi }}_{\mathbf{I}_{S_k}} e \right\| _2^2 \nonumber \\&\quad = \sigma _{S_k} p_{S_k} \left\| \nabla f_{\mathbf{I}_{S_k}, \mathbf{J}^{k}}(x^{k}) - \nabla f_{\mathbf{I}_{S_k}, \mathbf{J}^{k}}(x^*) \right\| _2^2, \end{aligned}$$

where in the last equation we used the identity

118 $$\begin{aligned}&\left\| \nabla f_{\mathbf{I}_C, \mathbf{J}}(x) -\nabla f_{\mathbf{I}_C, \mathbf{J}}(y) \right\| _2^2 \nonumber \\&\quad = \left\| \tfrac{1}{n p_C}({\varvec{\nabla }}{} \mathbf{F}(x) - {\varvec{\nabla }}{} \mathbf{F}(y)) {\varvec{\Pi }}_C e \right\| _2^2, \quad \forall \mathbf{J}\in {\mathbb {R}}^{d \times n}, \forall C \in {{\mathcal {G}}}, \end{aligned}$$

which in turn is a specialization of (44) to the minibatch sketch ${\mathbf {S}}=\mathbf{I}_S$ and the specific choice of the bias-correcting variable $\theta _{\mathbf {S}}= 1/p_S$. It remains to take expectation of (116) and (117), apply the tower property, and combine this with (115). $\square$

### Bounding the second moment of $g^k$

In the next lemma we bound the second moment of our gradient estimate $g^k$.

#### Lemma 11

The second moment of the gradient estimate is bounded by

119 $$\begin{aligned} {\mathbb {E}}\left[ \left\| g^k \right\| _2^2 \;|\; \mathbf{J}^k, x^k\right]\le & {} 2{\mathbb {E}}\left[ \left\| \nabla f_{\mathbf{I}_{S}, \mathbf{J}^k}(x^k)-\nabla f_{\mathbf{I}_{S}, \mathbf{J}^k}(x^*) \right\| _2^2 \;|\; \mathbf{J}^k,x^k\right] \nonumber \\&\quad +\,2{\mathbb {E}}\left[ \Big \Vert \nabla f_{\mathbf{I}_{S}, \mathbf{J}^{k}}(x^*)-\frac{1}{n}\mathbf{J}^{k}e\Big \Vert _2^2 \;|\; \mathbf{J}^k, x^k\right] . \end{aligned}$$

#### Proof

Adding and subtracting $\tfrac{1}{np_{S_k}} {\varvec{\nabla }}{} \mathbf{F}(x^*) {\varvec{\Pi }}_{\mathbf{I}_{S_k}} e$ from (108) gives

$$\begin{aligned} g^k = \frac{1}{n}\mathbf{J}^{k}e- \frac{1}{n p_{S_k}}(\mathbf{J}^{k} - {\varvec{\nabla }}{} \mathbf{F}(x^*)) {\varvec{\Pi }}_{\mathbf{I}_{S_k}} e+ \frac{1}{n p_{S_k}}({\varvec{\nabla }}{} \mathbf{F}(x^k)-{\varvec{\nabla }}{} \mathbf{F}(x^*)){\varvec{\Pi }}_{\mathbf{I}_{S_k}} e. \end{aligned}$$

Taking norm squared on both sides, and using the bound $\Vert a+b\Vert _2^2\le 2\Vert a\Vert _2^2 + 2\Vert b\Vert _2^2$ gives

120 $$\begin{aligned}&\left\| g^{k} \right\| _2^2 \le \frac{2}{n^2 p_{S_k}^2} \left\| ({\varvec{\nabla }}{} \mathbf{F}(x^k)-{\varvec{\nabla }}{} \mathbf{F}(x^*)){\varvec{\Pi }}_{\mathbf{I}_{S_k}} e \right\| _2^2 \nonumber \\&\qquad \qquad + \frac{2}{n^2 } \left\| \tfrac{1}{p_{S_k}}(\mathbf{J}^{k} - {\varvec{\nabla }}{} \mathbf{F}(x^*)) {\varvec{\Pi }}_{\mathbf{I}_{S_k}} e-\mathbf{J}^{k} e \right\| _2^2 \nonumber \\&\qquad \overset{(118)}{=} 2\left\| \nabla f_{\mathbf{I}_{S_k}, \mathbf{J}^k}(x^k)-\nabla f_{\mathbf{I}_{S_k}, \mathbf{J}^k}(x^*) \right\| _2^2 \nonumber \\&\qquad \qquad + \frac{2}{n^2} \underbrace{\left\| \tfrac{1}{p_{S_k}}(\mathbf{J}^{k} - {\varvec{\nabla }}{} \mathbf{F}(x^*)) {\varvec{\Pi }}_{\mathbf{I}_{S_k}} e-\mathbf{J}^{k} e \right\| _2^2}_{A}. \end{aligned}$$

Taking expectation of the *A* term, we get

$$\begin{aligned}&\mathbb {E}\left[ \left\| \underbrace{\tfrac{1}{p_S}(\mathbf{J}^{k} - {\varvec{\nabla }}{} \mathbf{F}(x^*)) {\varvec{\Pi }}_{\mathbf{I}_{S}} e}_{X} -\underbrace{\mathbf{J}^{k} e}_{{\mathbb {E}}\left[ X\right] }\right\| _2^2 \;|\; \mathbf{J}^k,x^k \right] \le {\mathbb {E}}\left[ \left\| \tfrac{1}{p_S}(\mathbf{J}^{k} - {\varvec{\nabla }}{} \mathbf{F}(x^*)) {\varvec{\Pi }}_{\mathbf{I}_S} e \right\| _2^2 \;|\; \mathbf{J}^k, x^k\right] \\&\quad \overset{(114)}{=} n^2 {\mathbb {E}}\left[ \left\| \nabla f_{\mathbf{I}_S, \mathbf{J}^{k}}(x^*) -\frac{1}{n}\mathbf{J}^{k}e \right\| _2^2 \;|\; \mathbf{J}^k, x^k\right] , \end{aligned}$$

where we used the inequality ${\mathbb {E}}\left[ \left\| X-{\mathbb {E}}\left[ X\right] \right\| _2^2\right] \le {\mathbb {E}}\left[ \left\| X \right\| _2^2\right]$. The result follows by combining the above with (120). $\square$

### Smoothness and strong convexity of $f_{\mathbf{I}_C, \mathbf{J}}$

Recalling the setting of Theorem 6, we assume that each $f_C$ is $\mu$—strongly convex and $L_C$—smooth:

$$\begin{aligned}&f_C(y) + \langle \nabla f_C(y), x-y \rangle + \frac{\mu }{2} \left\| x-y \right\| _2^2 \le f_C(x) \\&\quad \le f_C(y) + \langle \nabla f_C(y), x-y \rangle + \frac{L_C}{2} \left\| x-y \right\| _2^2 \end{aligned}$$

for all $C\in {{\mathcal {G}}}$. It is known (see Section 2.1 in [19]) that the above conditions imply the following inequality:

121 $$\begin{aligned} \left\langle \nabla f_C(x)-\nabla f_C(y), x-y\right\rangle\ge & {} \frac{\mu L_C }{\mu +L_C} \left\| x-y \right\| _2^2 \nonumber \\&+\frac{1}{\mu +L_C} \left\| \nabla f_C(x)-\nabla f_C(y) \right\| _2^2, \end{aligned}$$

for all $x,y \in {\mathbb {R}}^d$. A consequence of these assumptions that will be useful to us is that the function $f_{\mathbf{I}_C, \mathbf{J}}$ is $\frac{\tau \mu }{n p_C}$—strongly convex and $\frac{\tau L_C}{n p_C}$—smooth. This can in turn be used to establish the next lemma, which will be used in the proof of Theorem 6:

#### Lemma 12

Under the assumptions of Theorem 6 (in particular, assumptions on *f* and *S*), we have

122 $$\begin{aligned} \left\langle \nabla f(x)-\nabla f(y), x-y\right\rangle\ge & {} \frac{\mu }{2} \left\| x-y \right\| _2^2 \nonumber \\&+{\mathbb {E}}_{{{\mathcal {D}}}}\left[ \frac{n p_S}{2\tau L_S} \left\| \nabla f_{\mathbf{I}_{S}, \mathbf{J}}(x)-\nabla f_{\mathbf{I}_{S}, \mathbf{J}}(y) \right\| _2^2\right] , \qquad \end{aligned}$$

for all $x,y \in {\mathbb {R}}^d$ and $\mathbf{J}\in {\mathbb {R}}^{d \times n}$.

#### Proof

Applying (121) to the function $f_{\mathbf{I}_S, \mathbf{J}}$ gives

$$\begin{aligned} \left\langle \nabla f_{\mathbf{I}_S, \mathbf{J}}(x)-\nabla f_{\mathbf{I}_S, \mathbf{J}}(y), x-y\right\rangle\ge & {} \frac{\tau }{n p_S}\frac{ \mu L_S }{\mu +L_S} \left\| x-y \right\| _2^2\\&+\frac{n p_S}{\tau (\mu +L_S)}\left\| \nabla f_{\mathbf{I}_S, \mathbf{J}}(x)-\nabla f_{\mathbf{I}_S, \mathbf{J}}(y) \right\| _2^2\\\ge & {} \frac{\tau \mu }{2n p_S} \left\| x-y \right\| _2^2 \\&+\frac{n p_S}{2\tau L_S}\left\| \nabla f_{\mathbf{I}_S, \mathbf{J}}(x)-\nabla f_{\mathbf{I}_S, \mathbf{J}}(y) \right\| _2^2. \end{aligned}$$

Taking expectation over both sides over *S*, noting that ${\mathbb {E}}_{{{\mathcal {D}}}}\left[ \frac{1}{p_S}\right] = \sum _{C \in {{\mathcal {G}}}} 1=\frac{n}{\tau }$, and recalling that $\nabla f_{\mathbf{I}_S,\mathbf{J}}(x)$ is an unbiased estimator of $\nabla f(x)$, we get the result. $\square$

### Proof of Theorem 6

Let ${\mathbb {E}}_{k}\left[ \cdot \right]$ denote expectation conditional on $\mathbf{J}^k$ and $x^k$. We can write

123 $$\begin{aligned}&{\mathbb {E}}_{k}\left[ \left\| x^{k+1} -x^* \right\| _2^2 \right] \nonumber \\&\quad \overset{(2)}{=} {\mathbb {E}}_{k}\left[ \left\| x^k -x^* - \alpha g^{k} \right\| _2^2\right] \nonumber \\&\quad \overset{(33)}{=} \left\| x^k -x^* \right\| _2^2 -2\alpha \left\langle \nabla f(x^k), x^k -x^*\right\rangle + \alpha ^2{\mathbb {E}}_{k}\left[ \left\| g^{k} \right\| _2^2\right] \nonumber \\&\quad \overset{(122)}{\le } (1-\mu \alpha )\left\| x^k -x^* \right\| _2^2 -\alpha {\mathbb {E}}_{k}\left[ \frac{n p_S}{\tau L_S} \left\| \nabla f_{\mathbf{I}_S, \mathbf{J}^k}(x^k)-\nabla f_{\mathbf{I}_S, \mathbf{J}^k}(x^*) \right\| _2^2\right] \nonumber \\&\qquad \qquad +\, \alpha ^2{\mathbb {E}}_{k}\left[ \left\| g^{k} \right\| _2^2\right] \nonumber \\&\quad \overset{(119)}{\le } (1-\mu \alpha )\left\| x^k -x^* \right\| _2^2 + 2\alpha ^2{\mathbb {E}}_{k}\left[ \left\| \frac{1}{n}\mathbf{J}^{k}e-\nabla f_{\mathbf{I}_S, \mathbf{J}^{k}}(x^*) \right\| _2^2\right] \nonumber \\&\qquad \qquad +\, 2\alpha {\mathbb {E}}_{k}\left[ \left( \alpha -\frac{n p_S}{2\tau L_S} \right) \left\| \nabla f_{\mathbf{I}_S, \mathbf{J}^k}(x^k)-\nabla f_{\mathbf{I}_S, \mathbf{J}^k}(x^*) \right\| _2^2\right] . \end{aligned}$$

Next, after taking expectation in (123), applying the tower property, and subsequently adding the term $2\alpha {\mathbb {E}}\left[ \sigma _{S} \left\| \frac{1}{n}\mathbf{J}^{k+1}e- \nabla f_{\mathbf{I}_S,\mathbf{J}^{k+1}}(x^*) \right\| _2^2\right]$ to both sides of the resulting inequality, we get

124 $$\begin{aligned}&{\mathbb {E}}\left[ \varPsi _S^{k+1}\right] \le {\mathbb {E}}\left[ (1-\mu \alpha )\left\| x^k -x^* \right\| _2^2\right] \nonumber \\&\qquad +2\alpha {\mathbb {E}}\left[ \left( \alpha -\frac{n p_S}{2\tau L_S} \right) \left\| \nabla f_{\mathbf{I}_S, \mathbf{J}^k}(x^k)-\nabla f_{\mathbf{I}_S, \mathbf{J}^k}(x^*) \right\| _2^2\right] \nonumber \\&\qquad +\, 2\alpha ^2{\mathbb {E}}\left[ \left\| \frac{1}{n}\mathbf{J}^{k}e- \nabla f_{\mathbf{I}_S, \mathbf{J}^{k}}(x^*) \right\| ^2\right] +2\alpha {\mathbb {E}}\left[ \sigma _{S} \left\| \frac{1}{n}\mathbf{J}^{k+1}e- \nabla f_{\mathbf{I}_S,\mathbf{J}^{k+1}}(x^*) \right\| _2^2\right] \nonumber \\&\quad \overset{(113)}{\le } \mathbb {E}\left[ \underbrace{\left( 1-\mu \alpha \right) }_{\text {I}}\left\| x^k -x^* \right\| _2^2\right] \nonumber \\&\qquad +\, 2 \alpha \mathbb {E}\left[ \sigma _S \underbrace{\left( 1 -p_S +\frac{\alpha }{\sigma _S} \right) }_{\text {II}}\left\| \frac{1}{n}\mathbf{J}^{k}e- \nabla f_{\mathbf{I}_S, \mathbf{J}^{k}}(x^*) \right\| _2^2\right] \nonumber \\&\qquad +\, 2\alpha \mathbb {E}\left[ \underbrace{\left( \alpha +\sigma _{S} p_{S}-\frac{n p_S}{2\tau L_S} \right) }_{\text {III}} \left\| \nabla f_{\mathbf{I}_S, \mathbf{J}^k}(x^k)-\nabla f_{\mathbf{I}_S, \mathbf{J}^k}(x^*) \right\| _2^2\right] . \end{aligned}$$

Next, we determine a bound on $\alpha$ so that III $\le 0$. Choosing

125 $$\begin{aligned} \alpha +\sigma _{C} p_{C} -\frac{n p_C}{2\tau L_{C}} \le 0, \quad \forall C\in {{\mathcal {G}}}\Rightarrow \alpha \le \frac{n p_C}{2\tau L_{C}} - \sigma _{C} p_{C}, \quad \forall C\in {{\mathcal {G}}}, \end{aligned}$$

guarantees that III $\le 0$, and thus the last term in term in (124) can be safely dropped. Next, to build a recurrence and conclude the convergence proof, we bound the stepsize $\alpha$ so that II $\le$ I; that is,

126 $$\begin{aligned} 1- p_{C} +\frac{\alpha }{\sigma _{C} } \le 1- \alpha \mu , \quad \forall C\in {{\mathcal {G}}}\Rightarrow \alpha \le \frac{\sigma _{C} p_{C} }{\mu \sigma _{C}+1},\quad \forall C\in {{\mathcal {G}}}. \end{aligned}$$

Consequently,

$$\begin{aligned} {\mathbb {E}}\left[ \varPsi ^{k+1}_S\right]\le & {} {\mathbb {E}}\left[ (1-\mu \alpha )\left\| x^k -x^* \right\| _2^2\right] \\&+2 \alpha {\mathbb {E}}\left[ \sigma _{S}(1-\mu \alpha )\left\| \frac{1}{n}\mathbf{J}^{k}e- \nabla f_{\mathbf{I}_S, \mathbf{J}^{k}}(x^*) \right\| _2^2\right] \\= & {} (1-\mu \alpha ){\mathbb {E}}\left[ \varPsi ^k_S\right] . \end{aligned}$$

Since $\sigma _S = \frac{n}{4\tau L_{S}}$, in view of (125) and (126) the combined bound on $\alpha$ is

$$\begin{aligned} \alpha \le \min \left\{ \frac{np_C}{4 \tau L_C}, \frac{p_C }{\mu +4\frac{\tau }{n} L_{C}} \right\} = \frac{p_C }{\mu +4\frac{\tau }{n} L_{C}}, \quad \forall C \in {{\mathcal {G}}}. \end{aligned}$$

Hence, we have established the recursion (111).

### Calculating the iteration complexity in special cases

In this section we consider the special case of JacSketch analyzed via Theorem 6—minibatch SAGA with $\tau$—partition sampling—and look at further special cases by varying the minibatch size $\tau$ and probabilities. Our aim is to justify the complexities appearing in Table 1. In view of Theorem 6 the iteration complexity is given by

127 $$\begin{aligned} \max _{C\in {{\mathcal {G}}}} \left( \frac{1}{p_C} + \frac{\tau }{n p_C} \frac{4 L_C}{\mu }\right) \log \left( \frac{1}{\epsilon }\right) , \end{aligned}$$

where ${{\mathcal {G}}}=\mathrm{supp}(S)$. Complexity (127) is listed in line 2 of Table 1. The complexities in lines 4, 6, 8 and 14 arise as special cases of (127) for specific choices of $\tau$ and probabilities $p_C$.

- In line 4 we have *gradient descent*. This is obtained by choosing ${{\mathcal {G}}}= \{[n]\}$ (whence $p_{[n]}=1$, $\tau =n$ and $L_{[n]}=L$), which is why (127) simplifies to $\left( 1 + \frac{4 L}{\mu } \right) \log \left( \frac{1}{\epsilon }\right) .$
- In line 6 we consider *uniform SAGA*. That is, we choose $\tau =1$ and $p_i=1/n$ for all *i*. We have ${{\mathcal {G}}}= \{\{1\}, \{2\}, \ldots , \{n\}\}$ and $L_{\{i\}}=L_i$. Therefore, (127) simplifies to $\left( n + \frac{4 L_{\max }}{\mu } \right) \log \left( \frac{1}{\epsilon }\right) .$ This is essentially the same^16^ complexity result given in [6].
- In line 8 we consider *SAGA with importance sampling*. This is the same setup as above, except we choose
  128 $$\begin{aligned} p_i = \frac{\mu n +4 L_i}{\sum _{j=1}^n n\mu +4 L_j} , \end{aligned}$$
  which is the optimal choice minimizing the complexity bound in $p_1, \ldots ,p_n$. With these optimal probabilities, the stepsize bound becomes $\alpha \le \frac{1}{n\mu +4 \bar{L}},$ and by choosing the maximum allowed stepsize the resulting iteration complexity is
  129 $$\begin{aligned} \left( n +\frac{4 \bar{L}}{\mu } \right) \log \left( \frac{1}{\epsilon }\right) . \end{aligned}$$
  Now consider the probabilities $p_i = \frac{L_i}{\sum _{j=1}^n L_j}$ suggested in [30]. Using our bound, these lead to the complexity
  130 $$\begin{aligned} \max _{i=1,\ldots , n}\left\{ \frac{\sum _{j=1}^n L_j}{L_i}+4\frac{ \sum _{j=1}^n L_j}{\mu n } \right\} \log \frac{1}{\epsilon } = \left( \frac{n\bar{L} }{L_{\min }}+\frac{4\bar{L}}{\mu } \right) \log \left( \frac{1}{\epsilon }\right) .\qquad \end{aligned}$$
  Comparing this with (129), we see that this non-uniform sampling offers a significant speed up over uniform sampling if $n\mu \le L_{\min }.$ However, our complexity (129) is always better than both the uniform sampling sampling complexity $(n + L_{\max }/\mu )\log \left( \frac{1}{\epsilon }\right)$ and (130).
- Finally, in line 14 of Table 1 we optimize over probabilities $p_C$ directly; that is we extend the importance sampling described above to any $\tau$. Minimizing the complexity bound over the probabilities, and noting that $|{{\mathcal {G}}}| = \frac{n}{\tau }$, this leads to the rate
  131 $$\begin{aligned} \left( \frac{n}{\tau } +\frac{4\frac{1}{|{{\mathcal {G}}}|}\sum _{C \in {{\mathcal {G}}}}L_C}{\mu } \right) \log \left( \frac{1}{\epsilon }\right) . \end{aligned}$$
  This iteration complexity also applies to the reduced memory variant of SAGA (18). This is because Theorem 6 also holds for sketches ${\mathbf {S}}= e_S$ where *S* is a $\tau$—partition sampling. To see this, note that our analysis in this section relies on the orthogonality property (107) which also holds for ${\mathbf {S}}= e_S$ since (for $\mathbf{W}=\mathbf{I}$) we have:
  $$\begin{aligned} {\varvec{\Pi }}_{e_{C_1}} {\varvec{\Pi }}_{e_{C_2}} = \frac{1}{\tau }e_{C_1}(\underbrace{e_{C_1}^\top e_{C_2}}_{=0})e_{C_2}^\top \frac{1}{\tau }= 0,\quad \text{ for }\quad C_1,C_2 \in {{\mathcal {G}}},\quad C_1 \ne C_2. \end{aligned}$$
  Lemmas 10, 11 and 12 depend on the sketch through $\nabla f_{{\mathbf {S}}, \mathbf{J}}(x^*)$ only, which in turn depends on the sketch through ${\varvec{\Pi }}_{{\mathbf {S}}}e$, and it is easy to see that if either ${\mathbf {S}}= \mathbf{I}_S$ or ${\mathbf {S}}= e_S$, we have ${\varvec{\Pi }}_{{\mathbf {S}}}e = e_S.$

## Experiments

We perform several experiments to validate the theory, and also test the practical relevance of non-uniform SAGA (79) with the optimized probability distribution (128). All of our code for these experiments was written in Julia and can be found on github in https://github.com/gowerrobert/StochOpt.jl.

In our experiments we test either ridge regression

132 $$\begin{aligned} f(x) = \frac{1}{2n}\left\| \mathbf{A}^\top x - y \right\| _2^2 + \frac{\lambda }{2}\left\| x \right\| _2^2, \end{aligned}$$

or logistic regression

133 $$\begin{aligned} f(x) = \frac{1}{n}\sum _{i=1}^n \log \left( 1 +e^{-y_i\left\langle a_i,x\right\rangle }\right) +\frac{\lambda }{2}\left\| x \right\| _2^2, \end{aligned}$$

where $\mathbf{A}= [a_1,\ldots , a_n] \in {\mathbb {R}}^{d \times n},$ $y \in {\mathbb {R}}^n$ is the given data and $\lambda >0$ the regularization parameter.

### New non-uniform sampling using optimal probabilities

First we compare non-uniform SAGA using the new optimized importance probabilities (128) against using the probabilities $p_i = L_i \big /\overline{L}$ as suggested in [30]. When $n\mu$ is significantly smaller than $L_i$ for all *i* then the two sampling are very similar. But when $n\mu$ is relatively large, then the optimized probabilities (128) can be much closer to a uniform distribution as compared to using $p_i = L_i \big /\overline{L}$. We illustrate this by solving a ridge regression problem (132), using generated data such that

134 $$\begin{aligned} \mathbf{A}^\top x = y +\epsilon , \end{aligned}$$

where the elements of $\mathbf{A}$ and *x* are sampled from the standard Gaussian distribution ${{\mathcal {N}}}(0,1)$, and the elements of $\epsilon$ are sampled from ${{\mathcal {N}}}(0,10^{-3})$. It is not hard to see that the smoothness constants $\{L_i\}$ are given by $L_i=\left\| a_i \right\| _2^2+\lambda$ for $i\in [n]$. We scale the columns of $\mathbf{A}$ so that $\left\| a_1 \right\| _2^2 =1$ and $\left\| a_i \right\| _2^2 = \frac{1}{n^2},$ for $i=2,\ldots , n,$ and set the regularization parameter $\lambda = \frac{1}{n^2}.$ Consequently, $L_{\max } = 1+ \frac{1}{n^2}$, $L_{i} = \frac{2}{n^2}$ for $i=1,\ldots , n$, $\overline{L} = \frac{(n+1)^2 -1}{n^3}$ and $\mu = \tfrac{1}{n}\lambda _{\min }(\mathbf{A}\mathbf{A}^\top )+ \frac{1}{n^2}$. In this case the iteration complexity of non-uniform SAGA with the optimal probabilities (129) is given by

135 $$\begin{aligned} \left( n +4\frac{(n+1)^2 -1}{\mu n^3} \right) \log \left( \frac{1}{\epsilon } \right) . \end{aligned}$$

The complexity (130) which results from using the probabilities $p_i = L_i \big /\overline{L}$ is given by

136 $$\begin{aligned} \frac{(n+1)^2 -1}{ n^3} \left( \frac{n^3}{2}+\frac{4}{\mu } \right) \log \left( \frac{1}{\epsilon }\right) . \end{aligned}$$

Now we consider the regime where $n \rightarrow \infty ,$ in which case $\mu \rightarrow {{\mathcal {O}}}(\frac{1}{n^2})$ and consequently (135)$\rightarrow {{\mathcal {O}}}(n)\log \frac{1}{\epsilon }$ and in contrast (136) $\rightarrow {{\mathcal {O}}}(n^2)\log \frac{1}{\epsilon }.$

**Fig. 2:**
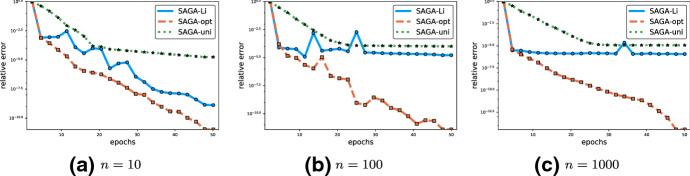
Comparing the performance of SAGA with importance sampling based on the optimized probabilities (128) (SAGA-opt), $p_i = L_i/\overline{L}$ (SAGA-Li) and $p_i = 1/n$ (SAGA-uni) for an artificially constructed ridge regression problem as *n* grows. Markers represent monitored points and not the iterations of the algorithms

We illustrate this in Fig. 1a-c where we set $n = 10$, $n = 100$ and $n = 1000$, respectively, and plot the complexities given in (135) and (136) . To accompany this plot, in Fig. 2a-c we also plot an execution of SAGA-uni (SAGA with uniform probabilities), SAGA-Li (SAGA with $p_i = L_i/\overline{L}$) and SAGA-opt (SAGA with optimized probabilities). In all figures we see that SAGA-opt is the fastest method. We can also see that SAGA-Li stalls in Fig. 2b and c when *n* is larger, performing even worst as compared to SAGA-uni.

### Optimal mini-batch size

Our analysis of the mini-batch SAGA is precise enough as to inform an optimal mini-batch size. For instance, consider $\tau$—nice sampling and the resulting iteration complexity (102). Theorem 3 states that for any $\tau \in [n]$, the terms within the maximum in (102) are bounded by

137 $$\begin{aligned} L_{\max }\ge & {} L^{{{\mathcal {G}}}}_{\max }\ge \quad L \end{aligned}$$

138 $$\begin{aligned} L_{\max } +\frac{\mu n}{4}\ge & {} \displaystyle C(\tau ) \,\,\overset{\text {def}}{=}\,\, \frac{1}{\tau }\frac{n-\tau }{n-1} L_{\max } +\frac{\mu }{4}\frac{n}{\tau }\ge \quad \frac{\mu }{4}. \end{aligned}$$

**Fig. 3:**
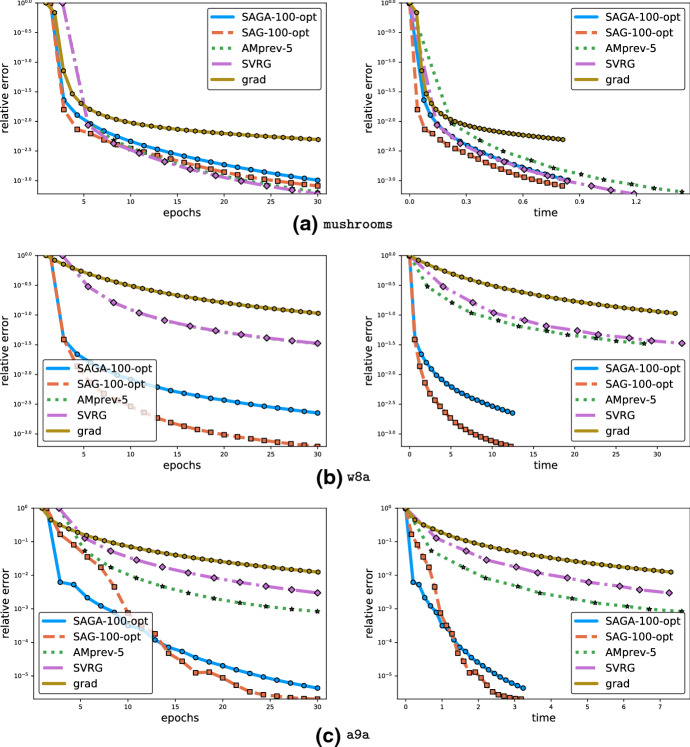
Comparison of the methods on logistic regression problems (133) with data taken from LIBSVM [4]

Moreover, the upper and lower bounds are realized for $\tau =1$ and $\tau =n$, respectively. Consequently, for $\tau$ small, we have $L^{{{\mathcal {G}}}}_{\max }\le C(\tau )$. On the other hand, for $\tau$ large we have $L^{{{\mathcal {G}}}}_{\max }\ge C(\tau ).$ Furthermore, $C(\tau )$ decreases super-linearly in $\tau$ while $L^{{{\mathcal {G}}}}_{\max }$ tends to decrease more modestly. Consequently, the point where $L^{{{\mathcal {G}}}}_{\max }$ overtakes $C(\tau )$ is often the best for the overall complexity of the method. To better appreciate these observations, we plot the evolution of the iteration complexity (102), the total complexity and the iteration complexity as predicted by Hofmann et al. [14] (see (105)) as $\tau$ increases in Fig. 3a–c for three different linear least squares problems. Since each step of mini-batch SAGA computes $\tau$ stochastic gradients, so the total complexity is $\tau$ times the iteration complexity. In each figure we can see that our iteration complexity initially decreases super-linearly, then at some point the complexity is dominated by $L^{{{\mathcal {G}}}}_{\max }$ and the iteration complexity decreases sublinearly. Up to this point we can observe an improvement in overall total complexity. This is in contrast to the iteration complexity given by Hofmann et al. that shows practically no improvement in even the iteration complexity as $\tau$ increases.

Though these experiments indicate only modest improvements in total complexity, and suggests that $\tau =2$ or $\tau =3$ is optimal, we must bear in mind that this corresponds to 10% and 20% of the data for these small dimensional problems. We conjecture that for larger problems, this improvement in total complexity will also be larger.

To use these insights in practice, we need to be able to efficiently determine the $\tau$ which corresponds to the point at which the convergence regimes switches from being dominated by $C(\tau )$ to being dominated by $L^{{{\mathcal {G}}}}_{\max }$. This surmounts to choosing $\tau$ so that $L^{{{\mathcal {G}}}}_{\max }= \frac{1}{\tau }\frac{n-\tau }{n-1} L_{\max } +\frac{\mu }{4}\frac{n}{\tau }.$ Estimating $L_{\max }$ and $\mu$ is often possible, but the cost of computing $L^{{{\mathcal {G}}}}_{\max }$ has a combinatorial dependency on *n* and $\tau .$ Thus to have a practical way of choosing $\tau$, we first need to bound $L^{{{\mathcal {G}}}}_{\max }$. This can be done for losses with linear classifiers using concentration bounds. We leave this for future work.

### Comparative experiments

We now compare the performance of SAGA-opt to several known methods such as SVRG [15], grad (gradient descent with fixed stepsizes) and AMprev (an improved version of SVRG that uses second order information) [28]. For the stepsize of SAGA-opt and SAG-opt, we found the stepsize $\alpha \le \frac{1}{n\mu + 4\bar{L}}$ given by theory to be a bit too conservative. Instead do we away with the 4 and used $\alpha = \frac{1}{n\mu + \bar{L}}$ instead. For the remaining methods we used a grid search over $L_{\max } \times 2^m$ for $m = 21, 19, 17, \ldots , -10,-11$.

To illustrate how biased gradient estimates can perform well in practice, we also test SAG-opt: a method that uses the same Jacobian updates as SAGA-opt, but instead uses the biased gradient estimate $g^k = \frac{1}{n}\mathbf{J}^{k+1} e$. See Sect. 2.5 for more details on biased gradient estimates.

In Fig. 3a–c we compare the methods on three logistic regression problems (133) based on three different data sets taken from LIBSVM [4]. In all these problems the two methods with optimized non-uniform sampling SAG-opt and SAGA-opt were faster in terms of both epochs and time. The next best method was AM-prev, followed by SVRG and grad. It is interesting to see how well SAG-opt performs in practice, despite having biased gradient estimates. This is why we believe it is important to advance the analyse of biased gradient estimates as future work.

## Conclusion

We now provide a brief summary of some of the key contributions of this paper and a few selected pointers to possible future research directions.

We developed and analyzed JacSketch—a novel family of variance reduced methods based on Jacobian sketching—and provided a link between variance reduction for empirical risk minimization and recent results from the field of randomized numerical linear algebra on sketch-and-project type methods for solving linear systems. In particular, it turns out that variance reduction is obtained by taking an SGD step on a stochastic optimization problem whose solution is the unknown Jacobian. As a consequence of our analysis, we resolved the conjecture of [30] in the affirmative by proving a properly designed importance sampling for SAGA leading to the iteration complexity of ${{\mathcal {O}}}(n + \tfrac{\bar{L}}{\mu } ) \log \left( \frac{1}{\epsilon } \right)$. For this purpose we developed a new proof technique using a *stochastic Lyapunov function*. Our complexity result for uniform mini-batch SAGA perfectly interpolates between the best known convergence rates of SAGA and gradient descent, and is sufficiently precise as to inform the choice of the batch size that minimizes the over all complexity of the method. Additionally we design and analyse a *reduced memory* variant of SAGA as a special case.

For future work we see many possible avenues including the following.

*Structured sparse weight matrices* One may wish to explore combinations of a weight matrix and different sketches to design new efficient methods further improving iteration complexity. For this the weighting matrix will have to be highly structured (e.g., block diagonal or very sparse) so that the Jacobian update (39) can be computed efficiently.

*Bias-variance trade-off* One can try to explore the bias-variance trade-off as opposed to merely focus on the extremes only: SAG (minimum variance) and SAGA (no bias). There is also no empirical evidence that unbiased estimators outperform the biased ones.

*Johnson–Lindenstrauss sketches* One can design completely new methods using different sparse sketches, such as the fast Johnson–Lindenstrauss transform [2] or the Achlioptas transform [1]. The resulting method can then be analyzed through Theorem 1. But first these sketches need to be adapted to ensure we get an efficient method. In particular, computing ${\varvec{\nabla }}{} \mathbf{F}(x) {\mathbf {S}}$ is only efficient if ${\mathbf {S}}$ most of the rows of ${\mathbf {S}}$ are zeros.

## Appendix A: Proof of inequality (20)

### Lemma 13

Let *S* be a sampling whose support ${{\mathcal {G}}}=\mathrm{supp}(S)$ is a partition of [*n*]. Moreover, assume all sets of this partition have cardinality $\tau$. Then

$$\begin{aligned} \frac{1}{|{{\mathcal {G}}}|}\sum _{C\in {{\mathcal {G}}}} L_C \le \bar{L} \le \max _{C\in {{\mathcal {G}}}} \frac{1}{\tau } \sum _{i\in C} L_i. \end{aligned}$$

### Proof

By assumption, $|{{\mathcal {G}}}|= \tfrac{n}{\tau }$. The first inequality follows from $\sum _{C \in {{\mathcal {G}}}} L_C \le \sum _{C\in {{\mathcal {G}}}} \frac{1}{\tau } \sum _{i\in C} L_i = \frac{1}{\tau } \sum _{i=1}^n L_i = \frac{n}{\tau } \bar{L}.$ On the other hand,

$$\begin{aligned} \bar{L} = \frac{1}{n} \sum _{i=1}^n L_i = \frac{1}{n} \sum _{C\in {{\mathcal {G}}}} \sum _{i\in C} L_i = \frac{1}{|{{\mathcal {G}}}|} \sum _{C\in {{\mathcal {G}}}} \frac{1}{\tau } \sum _{i\in C} L_i \le \max _{C\in {{\mathcal {G}}}}\frac{1}{\tau } \sum _{i\in C} L_i. \end{aligned}$$

$$\square $$

## Appendix B: Duality of sketch-and-project and constrain-and-approximate

### Lemma 14

Let $\mathbf{J}^k,{\varvec{\nabla }}{} \mathbf{F}\in {\mathbb {R}}^{d \times n}$ and ${\mathbf {S}}\in {\mathbb {R}}^{n \times \tau }.$ The sketch-and-project problem

139 $$\begin{aligned} \mathbf{J}^{k+1}= \arg \min _{\mathbf{J}\in {\mathbb {R}}^{d \times n}} \frac{1}{2}\left\| \mathbf{J}-\mathbf{J}^{k} \right\| _{\mathbf{W}^{-1}}^2 \quad \text{ subject } \text{ to } \quad {\varvec{\nabla }}{} \mathbf{F}{\mathbf {S}}= \mathbf{J}{\mathbf {S}}, \end{aligned}$$

and the constrain-and-approximate problem

140 $$\begin{aligned} \mathbf{J}^{k+1}= \underset{\mathbf{J}\in {\mathbb {R}}^{d \times n}}{\arg } \underset{\mathbf{Y}\in {\mathbb {R}}^{d \times \tau }}{\min } \frac{1}{2}\left\| \mathbf{J}-{\varvec{\nabla }}{} \mathbf{F} \right\| _{\mathbf{W}^{-1}}^2 \quad \text{ subject } \text{ to } \quad \mathbf{J}= \mathbf{J}^{k}+ \mathbf{Y}{\mathbf {S}}^\top \mathbf{W}, \end{aligned}$$

have the same solution, given by:

141 $$\begin{aligned} \mathbf{J}^{k+1} = \mathbf{J}^{k} -(\mathbf{J}^{k}-{\varvec{\nabla }}{} \mathbf{F}) {\mathbf {S}}({\mathbf {S}}^\top \mathbf{W}{\mathbf {S}})^{\dagger } {\mathbf {S}}^\top \mathbf{W}. \end{aligned}$$

### Proof

The proof is given in Theorem 4.1 in [12]. $\square$

## Appendix C: Proof of Theorem 5

First we will establish that

142 $$\begin{aligned} \frac{|{\mathcal {G}}|}{c_1^2}\sum _{C \in {{\mathcal {G}}}} e_C e_C^\top \mathbf{W}=\frac{|{{\mathcal {G}}}|c_2}{c_1^2}\left( \begin{array}{@{}ccccc@{}} \frac{c_1}{c_2} w_1 &{} w_2 &{} \cdots &{} \frac{}{}w_{n-1} &{} w_{n}\\ w_{1} &{} \frac{c_1}{c_2}w_2 &{} \cdots &{} w_{n-1} &{} w_{n} \\ \vdots &{} &{} \ddots &{} &{} \vdots \\ w_{1} &{} \cdots &{} &{} \frac{c_1}{c_2}w_{n-1} &{} w_{n} \\ w_{1} &{} w_{2} &{} \cdots &{} w_{n-1} &{} \frac{c_1}{c_2}w_n \end{array} \right) . \end{aligned}$$

Indeed, for every *i* we have that $e_i^\top \frac{|{\mathcal {G}}|}{c_1^2} \left( \sum _{C \in {{\mathcal {G}}}} e_C e_C^\top \mathbf{W}\right) e_i= w_i \frac{|{\mathcal {G}}|}{c_1^2} \sum _{C \in {{\mathcal {G}}}\, : \, i \in C} 1 = w_i \frac{|{\mathcal {G}}|}{c_1},$ and for every $i \ne j$ we have $e_i^\top \frac{|{\mathcal {G}}|}{c_1^2} \left( \sum _{C \in {{\mathcal {G}}}} e_C e_C^\top \mathbf{W}\right) e_j= w_j \frac{|{\mathcal {G}}|}{c_1^2}\sum _{C \in {{\mathcal {G}}}\, : \, i,j \in C} 1 = w_j \frac{| {{\mathcal {G}}}| c_2}{c_1^2}.$ Using (142), (91) and the Gershgorin circle theorem to bound $\rho$ from above we get $\rho \le \max _i \left\{ \left( \frac{|{\mathcal {G}}|}{c_1} -1\right) w_i+\sum _{i\ne j} w_j \left| \frac{| {{\mathcal {G}}}| c_2}{c_1^2}-1\right| \right\} ,$ as claimed. When $\mathbf{W}=\mathbf{I}$ we can get tighter results by using that $\left( \frac{|{\mathcal {G}}|}{c_1^2}\sum _{C \in {{\mathcal {G}}}} e_C e_C^\top -ee^\top \right)$ is a *circulant matrix* with associated vector $v = \left( \frac{| {{\mathcal {G}}}| }{c_1}-1 ,\frac{| {{\mathcal {G}}}| c_2}{c_1^2}-1, \ldots , \frac{| {{\mathcal {G}}}| c_2}{c_1^2}-1\right) \in {\mathbb {R}}^n.$ There is an elegant formula for calculating eigenvalues $\lambda _j$ of circulant matrices [34] using *v*, given by

143 $$\begin{aligned} \lambda _{j}= & {} v_1+ \sum _{k=1}^{n-1} \omega _j^{k} v_{n-k+1} = \frac{| {{\mathcal {G}}}| }{c_1}-1 \nonumber \\&+\left( \frac{| {{\mathcal {G}}}| c_2}{c_1^2}-1\right) \sum _{k=1}^{n-1} \omega _j^{k}, \quad \text{ for }\quad j=0,\ldots , n-1, \end{aligned}$$

where $\omega _j =e^{\frac{2 \pi {i}j}{n} }$ are the *n*-th roots of unity and ${i}$ is the imaginary number. From (143) we see that there are only two distinct eigenvalues. Namely, for $j =0$ we have

$$\begin{aligned} \lambda _0 \overset{(143)}{=} \frac{| {{\mathcal {G}}}| }{c_1}-1 +\left( \frac{ | {{\mathcal {G}}}| c_2}{c_1^2}-1\right) (n-1) = \frac{| {{\mathcal {G}}}| }{c_1}\left( 1+ (n-1) \frac{c_2}{c_1}\right) -n. \end{aligned}$$

The other eigenvalue is given by any $j \ne 0$ since

$$\begin{aligned} \lambda _j \overset{(143)}{=} \frac{| {{\mathcal {G}}}| }{c_1}-1 -\left( \frac{| {{\mathcal {G}}}| c_2}{c_1^2}-1\right) + \left( \frac{| {{\mathcal {G}}}| c_2}{c_1^2}-1\right) \underbrace{\sum _{k=0}^{n-1} \omega _j^{k}}_{=0} =\frac{| {{\mathcal {G}}}| }{c_1}\left( 1 - \frac{c_2}{c_1}\right) .\square \end{aligned}$$

## Appendix D: Notation glossary

See the Table 2.

**Table 2:** Frequently used notation

*f*(*x*)	$\tfrac{1}{n}\sum _{i=1}^n f_i(x)$ (convex loss function $f:{\mathbb {R}}^d\rightarrow {\mathbb {R}}$)	(1)
$x^*$	Minimizer of *f*	(1)
$\mu$	Strong convexity constant of *f*	Table 1 and Assumption 3.3 and Theorem 6
$\alpha$	Stepsize	(2)
$g^k$	Stochastic estimator of $\nabla f(x^k)$	(2), (13), (16), (33)
[*n*]	$$\{1,2,\ldots ,n\}$$	
*F*(*x*)	$(f_1(x),\ldots , f_n(x))^\top \in {\mathbb {R}}^n$ (function $F:{\mathbb {R}}^d\rightarrow {\mathbb {R}}^n$)	(3)
${\varvec{\nabla }}{} \mathbf{F}(x)$	$[\nabla f_1(x),\ldots , \nabla f_n(x)] \in {\mathbb {R}}^{d\times n}$ (Jacobian of *F* at *x*)	(4)
$e$	$(1,1,\ldots ,1)^\top \in {\mathbb {R}}^n$ (vector of all ones)	(5)
$f^*$/$f^k$	Shorthand for $f(x^*)$ / $f(x^k)$	
$\mathbf{W}$	$n\times n$ symmetric positive definite “weight” matrix	(10), (12)
$\left\| \mathbf{X} \right\| _{\mathbf{W}^{-1}}$	$(\text{ Tr }\left( \mathbf{X}\mathbf{W}^{-1} \mathbf{X}^\top \right) )^{1/2}$ (weighted Frobenius norm)	(10)
${\mathbf {S}}$	A random (sketching) $n\times \tau$ matrix picked from ${{\mathcal {D}}}$	
${\varvec{\Pi }}_{\mathbf {S}}$	${\mathbf {S}}({\mathbf {S}}^\top \mathbf{W}{\mathbf {S}})^{\dagger } {\mathbf {S}}^\top \mathbf{W}$ (stochastic projection matrix)	
$\theta _{{\mathbf {S}}}$	Bias-correcting random variable	(15) and Assumption 2.1
${\mathbb {E}}_{{{\mathcal {D}}}}\left[ \cdot \right]$	${\mathbb {E}}_{{\mathbf {S}}\sim {{\mathcal {D}}}}\left[ \cdot \right]$ (expectation over ${\mathbf {S}}\sim {{\mathcal {D}}}$)	
*S* or $S_k$	Sampling (a random subset of [*n*])	
$\tau$	${\mathbb {E}}\left[ |S|\right]$ (minibatch size)	
*C*	Subset of [*n*]	
$e_C$	$\sum _{i\in C} e_i$ ($e_i$ is the *i*th unit coordinate vector in ${\mathbb {R}}^d$)	
$p_C$/$p_i$	${\mathbb {P}}\left[ S=C\right]$/${\mathbb {P}}\left[ i\in S\right]$	Sections 1.4 and 4
$\mathbf{I}_C$	Column submatrix of $\mathbf{I}$ with columns indexed by *C*	Section 4 and Theorem 6
${{\mathcal {G}}}=\mathrm{supp}(S)$	$\{C \subseteq [n] \;:\; p_C>0\}$ (support of sampling *S*)	Section 4
$f_C$	$\frac{1}{|C|}\sum _{i \in C} f_i$ (subsampled loss function)	Section 4 and Theorems 3 and 6
$L_C$	Smoothness constant of $f_C$	Sections 1.5 and 4.4 and Theorems 3 and 6
$L_i$	Smoothness constant of $f_i$	Sections 1.5 and 4.4
$L_{\max }$	$\max _i L_i$	Sections 1.5 and 4.4 and Theorem 3
*L*	Smoothness constant of $f = \frac{1}{n}\sum _i f_i$	Sections 1.5 and 4.4 and Theorem 3
$\bar{L}$	$\tfrac{1}{n}\sum _{i=1}L_i$	Sections 1.5 and 4.4 and Theorem 3
${{\mathcal {L}}}_1$	Expected smoothness constant of the stochastic gradient	Assumption 3.1 and Theorem 1
${{\mathcal {L}}}_2$	Expected smoothness constant of the Jacobian	Assumption 3.2 and Theorem 1
$L^{{{\mathcal {G}}}}_i$	$\tfrac{1}{c_1}\sum _{C \; :\; C \in {{\mathcal {G}}}, \, i \in C} L_C$	
$L^{{{\mathcal {G}}}}_{\max }$	$\max _{i} L^{{{\mathcal {G}}}}_i$ (= ${{\mathcal {L}}}_1$ for $\tau$—uniform *S* with $c_1$—uniform support)	Sections 1.5 and 4.4 and Theorems 2 and 3
$\kappa$	Stochastic contraction number	Section 3.2 and Lemma 2 and Theorem 1
$\rho$	Sketch residual	(37) and Theorem 1 and Lemma 6
$\varPsi ^k$ / $\varPsi _S^k$	Lyapunov function/stochastic Lyapunov function	(52)/(109)
$$c_1 $$	$$|\{C \, : \, C \in \mathrm{supp}(S), \, 1 \in C\}|$$	Definition 2
$c_2$	$|\{C \, : \, C \in \mathrm{supp}(S), \, 1\in C; 2\in C\}|$	(94)

## Footnotes

1 For the purposes of this narrative it suffices to assume that stochastic gradients can be sampled at cost ${{\mathcal {O}}}(d)$.

2 We will not bother about the distribution from which it is picked at the moment. It suffices to say that virtually all distributions are supported by our theory. However, if we wish to obtain a practical method, some distributions will make much more sense than others.

3 The term “quasi-gradient methods” was popular in the 1980s [21], and refers to algorithms for solving certain stochastic optimization problems which rely on stochastic estimates of function values and their derivatives. In this paper we give the term a different meaning by drawing a direct link with quasi-Newton methods.

4 For some prior results on importance sampling for minibatches, in the context of QUARTZ, see [5].

5 A formal definition can be found in Assumption 4.2.

6 In this paper, a sampling is a random set-valued mapping with the sets being subsets of [*n*].

7 We prove inequality (20) in the Appendix; see Lemma 13.

8 SVRG is also built on a linear covariate model [15].

9 Excluding such trivial cases as when ${\mathbf {S}}$ is an invertible matrix and $\theta _{{\mathbf {S}}} =1$ with probability one, in which case $\mathbf{B}=0$.

10 A similar relation to (43) holds for the stochastic optimization reformulation of linear systems studied by Richtárik and Takáč [26]. Therein, this relation holds as an identity with ${{\mathcal {L}}}_1=1$ (see Lemma 3.3 in [26]). However, the function $f_{{\mathbf {S}}}$ considered there is entirely different and, moreover, $f(x^*)=0$ and $\nabla f_{{\mathbf {S}}}(x^*)=0$ for all ${\mathbf {S}}$.

11 The notion of a probability matrix associated with a sampling was first introduced in [25] in the context of parallel coordinate descent methods, and further studied in [22].

12 Recall that $p_i = {\mathbb {P}}\left[ i\in S\right]$ for $i\in [n]$, $p_C = {\mathbb {P}}\left[ S=C\right]$ for $C\subseteq [n]$ and $\mathbf{W}= \mathrm{Diag}(w_1,\ldots , w_n)\succ 0$.

13 Note that $c_1 =|\{C\in \mathrm{supp}(S) \;:\; 1\in C\}|$, and hence ${{\mathcal {L}}}_1$ has the form of a maximum over averages.

14 This is only possible when *n* is a multiple of $\tau$.

15 We can alternatively set ${\mathbf {S}}= e_S$ and the same results will hold.

16 With the difference being that in [6] the iteration complexity is $2\left( n+L_{\max }\big /\mu \right) \log \left( \frac{1}{\epsilon }\right) ,$ thus a small constant change.
